# High-quality phenotypic and genotypic dataset of barley genebank core collection to unlock untapped genetic diversity

**DOI:** 10.1093/gigascience/giae121

**Published:** 2025-02-11

**Authors:** Zhihui Yuan, Maximilian Rembe, Martin Mascher, Nils Stein, Axel Himmelbach, Murukarthick Jayakodi, Andreas Börner, Klaus Oldach, Ahmed Jahoor, Jens Due Jensen, Julia Rudloff, Viktoria-Elisabeth Dohrendorf, Luisa Pauline Kuhfus, Emmanuelle Dyrszka, Matthieu Conte, Frederik Hinz, Salim Trouchaud, Jochen C Reif, Samira El Hanafi

**Affiliations:** Leibniz Institute of Plant Genetics and Crop Plant Research (IPK), OT Gatersleben, 06466 Seeland, Germany; Leibniz Institute of Plant Genetics and Crop Plant Research (IPK), OT Gatersleben, 06466 Seeland, Germany; KWS SAAT SE & Co. KGaA, 37574 Einbeck, Germany; Leibniz Institute of Plant Genetics and Crop Plant Research (IPK), OT Gatersleben, 06466 Seeland, Germany; German Centre for Integrative Biodiversity Research (iDiv) Halle-Jena-Leipzig, 04103 Leipzig, Germany; Leibniz Institute of Plant Genetics and Crop Plant Research (IPK), OT Gatersleben, 06466 Seeland, Germany; Crop Plant Genetics, Institute of Agricultural and Nutritional Sciences, Martin-Luther-University of Halle-Wittenberg, 06120 Halle (Saale), Germany; Leibniz Institute of Plant Genetics and Crop Plant Research (IPK), OT Gatersleben, 06466 Seeland, Germany; Leibniz Institute of Plant Genetics and Crop Plant Research (IPK), OT Gatersleben, 06466 Seeland, Germany; Leibniz Institute of Plant Genetics and Crop Plant Research (IPK), OT Gatersleben, 06466 Seeland, Germany; KWS LOCHOW GmbH, 29303 Bergen, Germany; Nordic Seed Germany GmbH, 31688 Nienstädt, Germany; Nordic Seed Germany GmbH, 31688 Nienstädt, Germany; Limagrain GmbH, 31226 Peine-Rosenthal, Germany; Nordsaat Saatzucht GmbH, Zuchtstation Gudow, D-23899 Gudow, Germany; Syngenta France SAS, 31790, Saint-Sauveur, France; Syngenta France SAS, 31790, Saint-Sauveur, France; Syngenta France SAS, 31790, Saint-Sauveur, France; Saatzucht Bauer GmbH & CO.KG, 93083 Obertraubling, Germany; Secobra Saatzucht GmbH, 85368 Moosburg an der Isar, Germany; Leibniz Institute of Plant Genetics and Crop Plant Research (IPK), OT Gatersleben, 06466 Seeland, Germany; Leibniz Institute of Plant Genetics and Crop Plant Research (IPK), OT Gatersleben, 06466 Seeland, Germany

**Keywords:** Barley, plant genetic resources, elite, whole genome resequencing, disease resistance, agronomic traits

## Abstract

**Background:**

Genebanks around the globe serve as valuable repositories of genetic diversity, offering not only access to a broad spectrum of plant material but also critical resources for enhancing crop resilience, advancing scientific research, and supporting global food security. To this end, traditional genebanks are evolving into biodigital resource centers where the integration of phenotypic and genotypic data for accessions can drive more informed decision-making, optimize resource allocation, and unlock new opportunities for plant breeding and research. However, the curation and availability of interoperable phenotypic and genotypic data for genebank accessions is still in its infancy and represents an obstacle to rapid scientific discoveries in this field. Therefore, effectively promoting FAIR (i.e., findable, accessible, interoperable, and reusable) access to these data is vital for maximizing the potential of genebanks and driving progress in agricultural innovation.

**Findings:**

Here we provide whole genome sequencing data of 812 barley (*Hordeum vulgare* L.) plant genetic resources and 298 European elite materials released between 1949 and 2021, as well as the phenotypic data for 4 disease resistance traits and 3 agronomic traits. The robustness of the investigated traits and the interoperability of genomic and phenotypic data were assessed in the current publication, aiming to make this panel publicly available as a resource for future genetic research in barley.

**Conclusions:**

The data showed broad phenotypic variability and high association mapping potential, offering a key resource for identifying genebank donors with untapped genes to advance barley breeding while safeguarding genetic diversity.

## Data Description

### Context

Successful plant breeding programs rely on balanced efforts between short-term goals to develop competitive cultivars and the maintenance of a broad genetic pool to guarantee long-term progress. In practice, the development of new varieties has been predominantly derived by recycling existing elite lines, leading to important genetic improvement and the reduction in the genetic diversity of elite germplasm. This could impede the breeding of potential new varieties capable of addressing and responding to constraints related to climate change, agronomical threads, and meeting the escalating social demands [1]. To overcome these limitations, leveraging genetic diversity harbored within plant genetic resources (PGRs) has been frequently suggested [2]. PGRs provide a valuable reservoir of untapped genetic potential that can be utilized to develop varieties with improved yield [3] and end-use quality, as well as enhanced resistance to both biotic and abiotic stresses, such as diseases [4], pests [5], waterlogging [6], salinity [7], and drought [6, 8].

As the most cost-effective *ex situ* conservation strategy, genebanks worldwide are committed to maintaining PGRs, which hold a diverse gene pool encompassing all the alleles of various genes, including those from wild species, landraces, and breeding stocks. However, although enormous efforts have been made to conserve germplasm [9], it is estimated that less than 1% of the resources preserved in genebanks have been used in crop improvement [10]. The great challenge for breeders and scientists lies in finding useful barley PGRs among entire genebank collections that comprise thousands of accessions with complex patterns of genetic diversity [11]. Therefore, core collections were proposed as a strategy to streamline operational processes and mitigate costs, thereby facilitating more precise and effective research and breeding initiatives. Over the past decades, this approach has become even more attractive thanks to recent technological advancements, which have markedly reduced the costs of genotyping and led to dramatic improvements in read length, sequencing chemistry, instrumentation, and throughput [12]. As a result, generating large-scale sequencing and genotyping datasets for entire genebank collections is now feasible. This has greatly expanded the scope of genotyping efforts and underpinned the effective selection of core collections that maximize genetic diversity [13]. These advancements provide powerful tools to efficiently harness PGRs, enabling the identification of valuable and favorable genes. This has streamlined their incorporation into crop improvement efforts, ultimately speeding up the development of new and improved varieties. Coupled with extensive and high-quality phenotypic data, the systematic use of whole genome sequencing data could provide valuable insights into genetic diversity and potential breeding opportunities. Our recent findings using genome-wide association analyses highlighted the value of these data in selecting donors with potentially novel favorable genes [14].

Moreover, the strategic deployment of core collections becomes even more compelling when combined with modern elite material [15, 16], which serves as a reference panel to define favorable alleles/genes that are absent in the elite panel. This integrated approach is essential for enhancing polygenic traits and, hence, achieving informed prebreeding decisions. To put this into practice, we selected a barley core collection [17] from the German Federal *ex situ* Genebank for Agriculture and Horticultural Crops at the Leibniz Institute of Plant Genetics and Crop Plant Research (IPK) and combined it with a set of European elite material. This population was designed to (i) phenotype the whole population in multienvironmental trials for 3 agronomical traits: plant height (PLH), heading date (HD), and lodging (LOD) and 4 disease traits: *Puccinia hordei* (PUC), *Blumeria graminis hordei* (BLU), *Ramularia collo-cygni* (RAM), and *Rhynchosporium commune* (RHY); (ii) evaluate the interoperability quality for the phenotypic and genomic datasets using 5-fold cross-validation; and (iii) conduct the mantel test to check the detection power in association mapping analyses.

The data presented here can be further extended with additional PGRs and/or elite materials. They can also be integrated with alternative strategies to improve the utilization of germplasm collections by selecting untapped PGR donors, such as the development of novel association mapping methods. This will enable breeders to make more accurate predictions of trait performance, thereby enhancing the efficiency of selection processes. Furthermore, with the development of publicly accessible resources, scientists will be able to focus more on research and innovation while reducing the burden of extensive phenotyping. The insights derived from our data may significantly accelerate advancements in genomic research and breeding programs, driving improvement and fostering future collaboration and resource sharing.

## Methods

### Barley material and field trials

To capture a broad spectrum of geographic origins and wide genetic diversity, we selected 812 PGRs, which include 288 spring type (PGR_Spring) and 524 winter type (PGR_Winter), originating from 57 countries spanning 5 continents. Based on their performance during seed regeneration, these PGRs were thoughtfully selected from a previously described barley core 1000 collection [17], as a representative subset of the entire 21,405 barley accessions available at the IPK genebank [18], based on their performance during seed regeneration. Additionally, we incorporated 298 elite lines, including 10 local checks, which consist of 128 spring type (Elite_Spring) and 170 winter type (Elite_Winter). These elites were exclusively selected from the European registered varieties and were available through the seed market, showcasing the breeding process over time from 1949 to 2021. The study initially included 87 additional genotypes that were later excluded from certain analyses due to incomplete phenotypic or genotypic data. To maintain the integrity of the dataset and facilitate accurate adjustments for experimental design effects, we retained all relevant data, including instances of missing information.

Field trials were conducted over 3 consecutive years (2020, 2021, and 2022) across 8 locations in Germany: KWS-L/Prosselsheim (49°51′15.6″N, 10°06′04.1″E; 10.9°C average annual temperature; 565.3 mm average annual rainfall), Nordic Seed/Nienstädt (52°17′35.52″N, 9°08′57.156″E; 10.7°C average annual temperature; 638.4 mm average annual rainfall), Saatzucht Bauer/Riekofen (48°54′55.98″N, 12°21′21.744″E; 9.9°C average annual temperature; 690.3 mm average annual rainfall), Limagrain/Peine-Rosenthal (52°18′09.828″N, 10°10′28.488″E; 10.9°C average annual temperature; 607.5 mm average annual rainfall), Nordsaat/Gudow (53°33′28.0″N, 10°47′50.5″E; 10.4°C average annual temperature; 581.1 mm average annual rainfall), Syngenta/Bad Salzuflen (52°04′21.576″N, 8°41′55.86″E; 10.5°C average annual temperature; 692.8 mm average annual rainfall), Secobra-LEM/Lemgo (52°00′41.6″N, 8°52′22.7″E; 10.7°C average annual temperature; 714.3 mm average annual rainfall), and Secobra-FK/Moosburg (48°28′46.8″N, 11°54′32.6″E; 10.7°C average annual temperature; 743.1 mm average annual rainfall). The trials were sown following a generalized alpha lattice design, which organizes genotypes into incomplete blocks to minimize spatial variation. Two-row observation plots (1 m^2^) with 2 replications were used, and 10 checks were included across years and locations for consistency. Each unique combination of year and location was considered a distinct environment.

### Phenotyping

The whole population was phenotyped for 3 agronomy traits for their importance in barley adaptability, yield potential, and harvestability: heading date measured in days from January 1 for winter type and from the sowing date onward for the spring type, plant height measured from the soil surface to the tip of spike in centimeters (excluding awns), and lodging rated on a 1–9 scale (with a higher score indicating severe lodging). Additionally, 4 disease traits, including *P. hordei, B. graminis hordei, R. collo-cygni*, and *R. commune*, were evaluated under natural infection conditions. The disease severities were scored using an ordinal scale from 1 (fully resistant) to 9 (fully susceptible) following the guidelines of the German Federal Plant Variety Office [19].

### Phenotypic data analyses

A linear mixed model using restricted maximum likelihood (REML) method [20] was used for data analyses across environments for spring and winter barley separately. Phenotypic data were corrected for outliers following the method of Tukey and Anscombe [21]. The residuals were extracted and then normalized to flag the outliers according to a predefined significance threshold of *P* < 0.01 (Supplementary Table S2). Variance components and best linear unbiased estimations (BLUEs) of each genotype were computed from the outlier-corrected data, following model (1):


(1) \begin{eqnarray*}
{y_{\textit{ijkm}}} = \mu + {E_m} + {g_i} + {g_i} \times {E_m} + {E_m}:{r_j}\;:{b_k} + {e_{\textit{ijkm}}},
\end{eqnarray*}

where *y_ijkm_* denoted the vector of phenotypic values for the *i*th genotype (*g*) tested in the *k*th block (*b*) nested in the *j*th replication (*r*) in *m*th environment (*E*), *μ* was the common mean, and *e* denoted the error term of the model. We assumed that all random effects followed an independent normal distribution with different variance components. In the model (1), all terms except *µ* and *g_i_* were considered random for deriving the BLUEs across environments, whereas all terms except *µ* were modeled as random to estimate the variance component for deriving heritability, following model (2):


(2) \begin{eqnarray*}
{H^2} = \frac{{\sigma _g^2}}{{\sigma _g^2 + \frac{{\sigma _{g \times E}^2}}{{\overline {{n_E}} }} + \frac{{\sigma _e^2}}{{\overline {{n_R}} }}}},
\end{eqnarray*}

where $\sigma _g^2$ denoted the genotypic variance, $\sigma _{g \times E}^2$ denoted the interaction between genotype and environment, $\sigma _e^2$ denoted the residual variance, $\overline {{n_R}} $ denoted the average number of replications per genotype, and $\overline {{n_E}} $ denoted the average number of environments in which the genotypes were evaluated. ASReml-R [22] was employed for all mixed linear models that were applied in the phenotypic analysis.

### Whole genome shotgun sequencing

Whole genome sequencing (WGS) of the 1,110 genotypes (812 PGRs and 298 elite lines) was performed at IPK Gatersleben. High molecular weight (HMW) DNA was extracted from the leaves (8 g) of greenhouse-grown (21°C/18°C day/night temperature) 7-day-old seedlings following a previously established protocol [23]. The Illumina Nextera libraries were prepared and sequenced using the Illumina NovaSeq 6000 platform [24]. Raw sequencing reads were trimmed using cutadapt [version 3.3; 25] and aligned to the MorexV3 reference genome [26] using Minimap2 [version 2.20; 27]. The resultant alignment records were sorted with Novosort (V3.09.01; http://www.novocraft.com). Finally, a total of 149,380,812 single nucleotide polymorphisms (SNPs) for the 1,110 genotypes were initially outputted by BCFtools [version 1.9; 28].

### Quality control for SNP data

The resulting raw genotypic data were used to extract the corresponding datasets of the 4 subgroups. Only biallelic SNPs with a minor allele frequency >0.05 and missing rate <0.1 were retained by PLINK [version 1.9; 29] for each of the 4 subgroups. These meticulous steps yielded datasets comprising 17,759,260 SNPs for Elite_Spring, 26,903,811 for Elite_Winter, 54,934,336 for PGR_Spring, and 46,434,685 for PGR_Winter.

The resulting filtered genotypic data were used as input to phase and impute missing values using Beagle [version 5.2; 30], leveraging linkage disequilibrium to infer missing data accurately. Subsequently, an *r*^2^ cutoff of 0.2 was set to prune markers by PLINK (version 1.9) with a sliding window size of 50 kb and a step size of 10 kb. The final number of SNPs available differed in the 4 subgroups due to the aforementioned process: 710,855 of Elite_Spring, 945,074 of Elite_Winter, 2,321,327 of PGR_Spring, and 1,775,972 of PGR_Winter. For each tested SNP, homozygous for the most frequent allele, heterozygous, and homozygous for the alternative allele were coded as 0, 1, and 2 by PLINK (version 1.9), respectively.

### Population structure

Subsequently, the aforementioned post–quality control markers were used to investigate the population structure within and across spring and winter barley accessions using principal coordinate analysis (PCoA) based on pairwise Rogers’s distance [31]. PCoA was performed using the R package ape [version v5.7–1; 32]. Additionally, the population structure was tested using ADMIXTURE [version 1.3.0; 33]. The optimal number of population components was determined based on cross-validation function (–cv).

Moreover, linkage disequilibrium (LD) analyses of the 4 subgroups was carried out separately by determining the pairwise squared allele-frequency correlations (*r*^2^) between markers [34] and then combined to estimate LD decay across the entire genome. A decay curve was fitted for each subgroup using nonlinear regression of pairwise *r*^2^ against the distance (Mb) between the markers. LD within a specific physical distance of 2 Mb was calculated and visualized using PopLDdecay [version 3.40; 34].

### Genomic–phenotypic data interoperability

To evaluate the interoperability for the phenotypic and genomic datasets, we calculated the accuracy of the genomic best linear unbiased prediction (GBLUP) [35]. First, the mixed-model equations for genomic prediction were computed using REML in the rrBLUP R package [v4.6.1; 36]. Prediction accuracies were then estimated through 5-fold cross-validation. In this process, both phenotypic and genomic datasets were randomly subdivided into 5 groups. The first 4 groups served together as the training set, whereas the fifth group corresponded to the prediction set. The random sampling was repeated 100 times, giving a total of 500 cross-validation runs. Genomic prediction ability was thereafter defined as the correlation between BLUEs across environments for a trait and the corresponding predicted values.

### Mantel correlation

Following the imputation process, we used PLINK (version 1.9) to construct a genetic relationship matrix. To further explore the association between phenotypic variation and population structure, the correlation between the genetic relationship matrix and the absolute trait differences (Euclidean distance matrix) in each subgroup was tested using a Mantel test [37] implemented in the R package vegan [v2.6–4; 36] and visualized by linkET R package [v0.0.7.4; 38]; 999 permutations were used to evaluate the significance of the test.

## Data Validation and Quality Control

### High heritability estimation highlights the robustness of the phenotypic data

**Figure 1:**
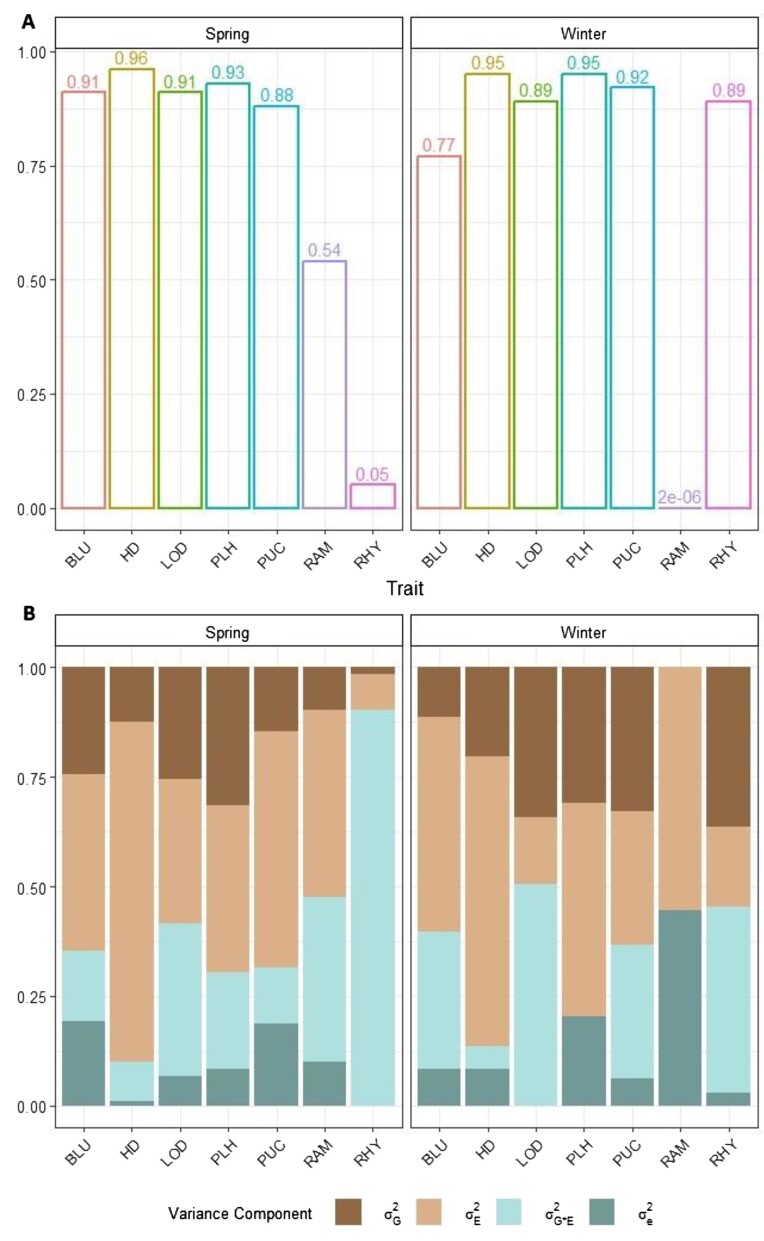
Heritability (A) and percentages of the different variance components (B) for the 7 traits considered in this study. BLU: *Blumeria graminis hordei*; HD: heading date; LOD: lodging; PLH: plant height; PUC: *Puccinia hordei*; RAM: *Ramularia collo-cygni*; RHY: *Rhynchosporium commune*; σ^2^_G_: genotypic variance; σ^2^_G*E_: variance due to genotype × environment interaction; σ^2^_E_: variance due to environment; σ^2^_e_: residual.

The quality and reliability of the phenotypic data were rigorously assessed by estimating the heritability of the evaluated traits. After outlier correction, the heritability estimates for most traits were generally high, exceeding 0.5 (Fig. 1A). Notable exceptions included RHY in the spring population (h^2^ = 0.05) and RAM (h^2^ = 2E-06) in the winter population. Variance components analysis revealed that environment ($\sigma _e^2$) accounts for the largest proportion of the total variance, while genotype and genotype × environment interaction were less pronounced, with the exception of LOD and RHY in both the spring and winter population, as well as PUC in the winter population (Fig. 1B). This suggests that factors such as temperature fluctuations, varying levels of precipitation, and humidity across different climate zones may have influenced the observed phenotypic performance. These environmental conditions likely influenced growth patterns and trait expression, leading to larger phenotypic variability in traits with high heritability and restricted variability in traits with low heritability.

**Figure 2:**
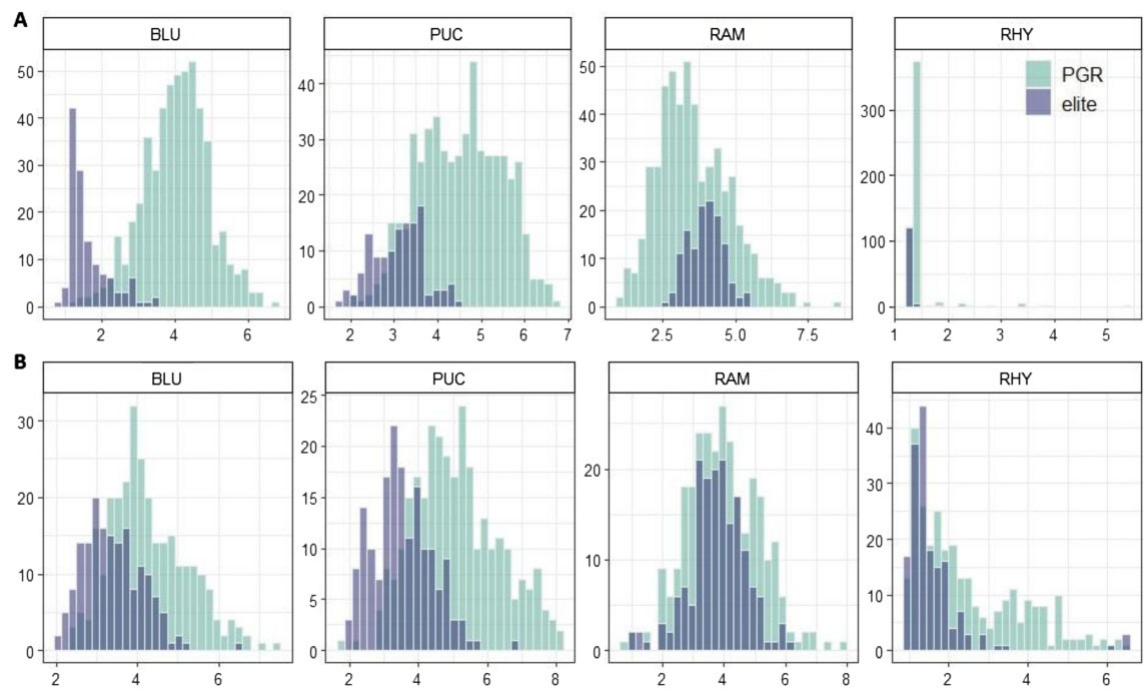
Histogram showing the phenotypic distribution for 4 disease traits for the spring (A) and winter (B) population. BLU: *Blumeria graminis hordei*; PUC: *Puccinia hordei*; RAM: *Ramularia collo-cygni*; RHY: *Rhynchosporium commune*.

**Figure 3:**
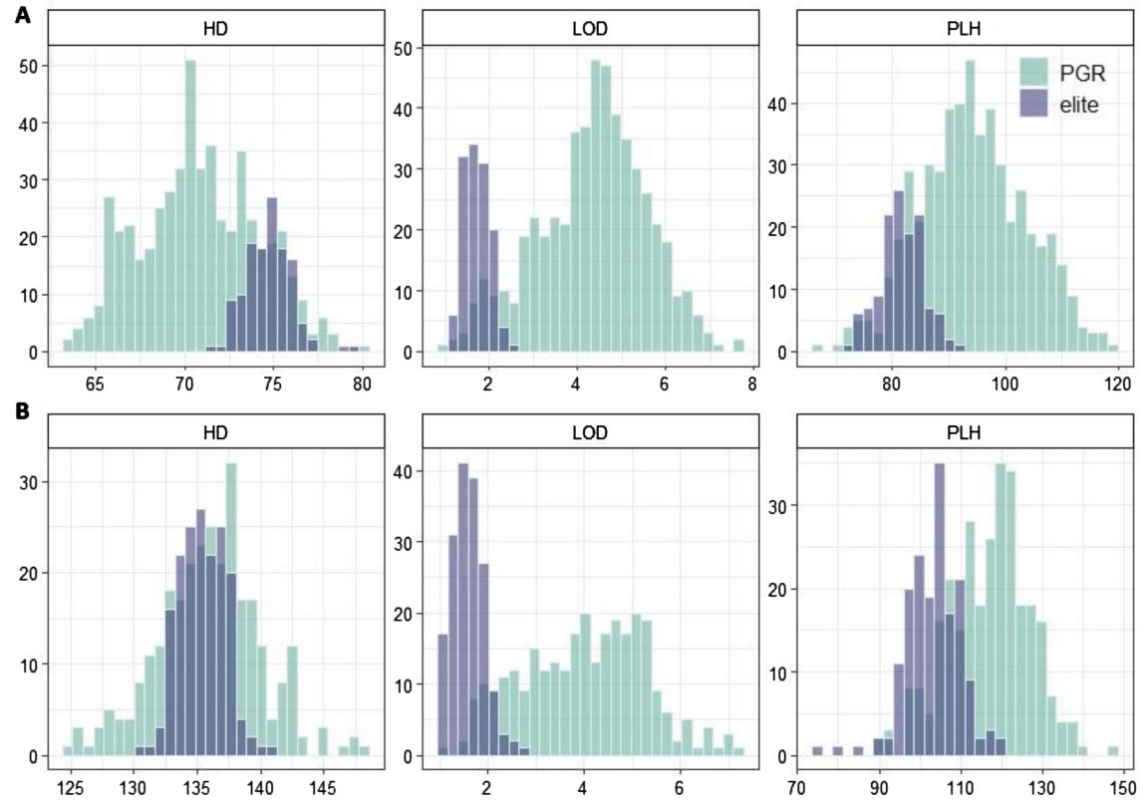
Histogram showing the phenotypic distribution for 3 agronomic traits for the spring (A) and winter (B) population. HD: heading date; LOD: lodging; PLH: plant height.

The resulting BLUEs showed a normal distribution for most disease traits (Fig. 2). However, RHY showed left skew in both spring and winter population, while BLU and PUC displayed left skew in the elite population for both spring and winter type. The left skew of RHY suggests low disease pressure across 3 years, hence resulting in a small proportion of susceptible genotypes. The left skew of elite population of BLU and PUC also suggests that PGRs tend to be more susceptible than the elite materials for the 2 diseases. For agronomic traits (Fig. 3), the PGR population showed a normal distribution, while elite lines showed a normal distribution in HD and PLH only in the winter population.

**Figure 4:**
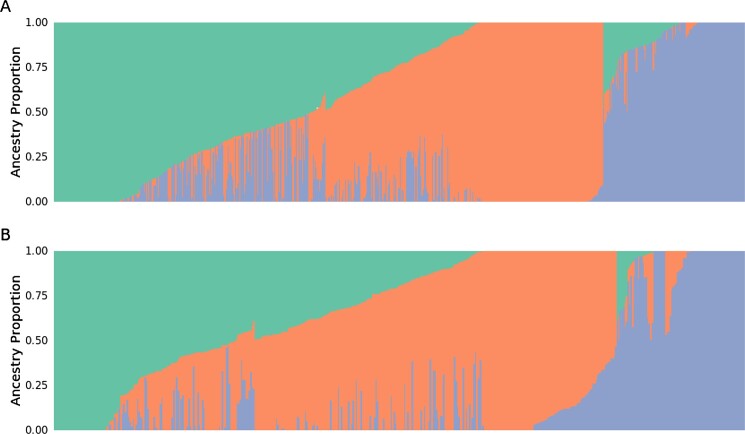
Admixture analysis of the spring (A) and winter (B) populations with the K = 3 admixture model. Each individual is represented as a vertical bar with color corresponding to the proportions of 3 ancestral components (K).

Furthermore, several significant correlations were observed between the evaluated traits (Fig. 4). For pairing of agronomic and disease traits, it was observed that HD was negatively correlated with all the disease traits, except for BLU in the winter barley population. Those observations suggest a strategic plant response given that delayed heading allows plants to evade disease infection through spatial or temporal adjustments. Moreover, LOD was positively correlated with all the disease traits, except for RAM in the spring barley population. PLH was positively correlated with BLU and PUC while negatively correlated with RAM and RHY.

### Whole genome sequencing data show high genetic diversity and high marker densities

WGS data of the 1,110 genotypes showed an average coverage of 4.7×, with a range spanning from 0.5× to 22.6× across all samples with a mapping rate from 94% to 99%, providing a solid foundation for downstream genetic analyses and ensuring a comprehensive representation of the genomic information across the diverse set of genotypes.

Building on this comprehensive genomic dataset, we performed PCoA to assess the genetic diversity among the spring and winter barley population, as reported in our companion study [14]. The first 2 coordinates together explained 11.66% and 11.25% of the spring and winter population, respectively. As anticipated, the inclusion of PGRs significantly broadened the genetic diversity compared to the elite materials. Notably, the elite spring population formed a tight, cohesive cluster, indicating less genetic diversity, while the elite winter population exhibited a more dispersed pattern, reflecting greater genetic variability.

To further complement the population structure analyses, the optimal number of genetic components (K = 3) was determined based on cross-validation results. The admixture analysis revealed distinct population structures within the spring and winter populations (Fig. 4), with individuals showing varying proportions of the 3 inferred components. These results highlight the contrasting levels of genetic diversity and population structure within each spring and winter barley genotype.

For the intrachromosomal decay of LD (*r*^2^), PGR was faster in both the spring and winter population as compared to elite materials. The slower LD decay in the elite population may be due to genetic bottlenecks and/or high selection pressures that produce specific linkage between alleles that control specific phenotypes.

### High genomic prediction accuracies support the interoperability of genomic and phenotypic data

Systematic errors can occur during field trials, which will systematically disrupt the connectivity between genotype and phenotype data and, in turn, decrease the value of the data for subsequent integrated analyses. To assess potential data imbalances, we used the cross-validated accuracy of genomic prediction as a quality measure for genomic–phenotypic data interoperability.

**Figure 5:**
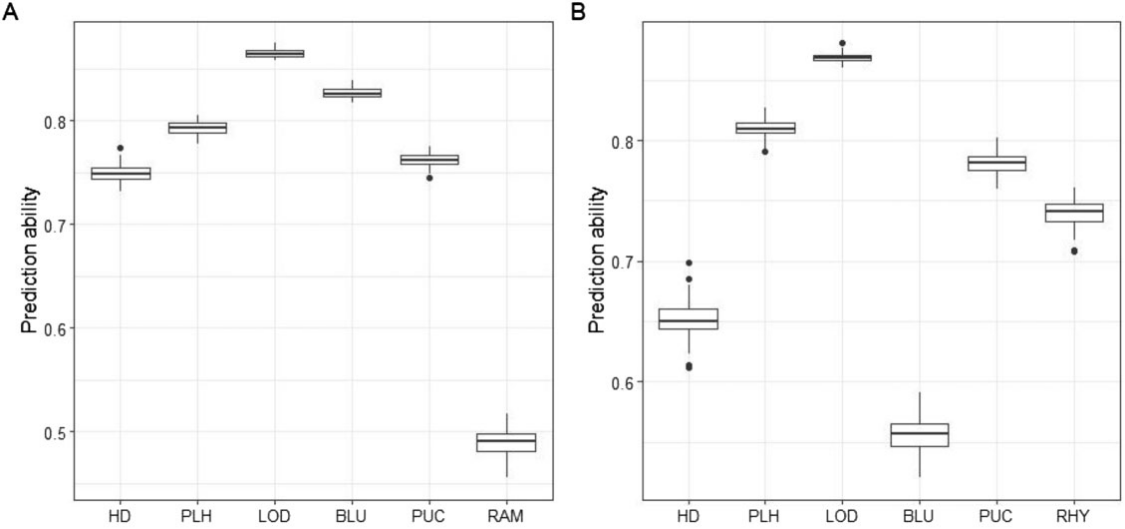
Fivefold cross-validation abilities of the genomic best linear unbiased prediction for heading date (HD; days), plant height (PLH; cm), lodging (LOD), *Blumeria graminis hordei* (BLU), *Puccinia hordei* (PUC), *Rhynchosporium commune* (RHY), and *Ramularia collo-cygni* (RAM), obtained in the spring (A) and winter (B) populations.

Integrating phenotypic data with WGS data resulted in 652 spring and 458 winter barley genotypes. Overall, the genomic–phenotypic data interoperability was in general high (Fig. 5), with a maximum prediction accuracy observed for lodging in both spring and winter populations. Disease-resistant traits showed moderate to high prediction abilities, suggesting that genomic data can be reliably used, thereby potentially accelerate breeding efforts for resistant varieties. In parallel, this robust result ensures reliable data quality, enabling comprehensive analyses to explore genotype–phenotype relationships and lay a solid foundation for future studies aimed at finding marker-trait associations and understanding the genetic mechanisms underlying key traits in barley.

### Mantel test results indicate a high detection power in association mapping

**Figure 6:**
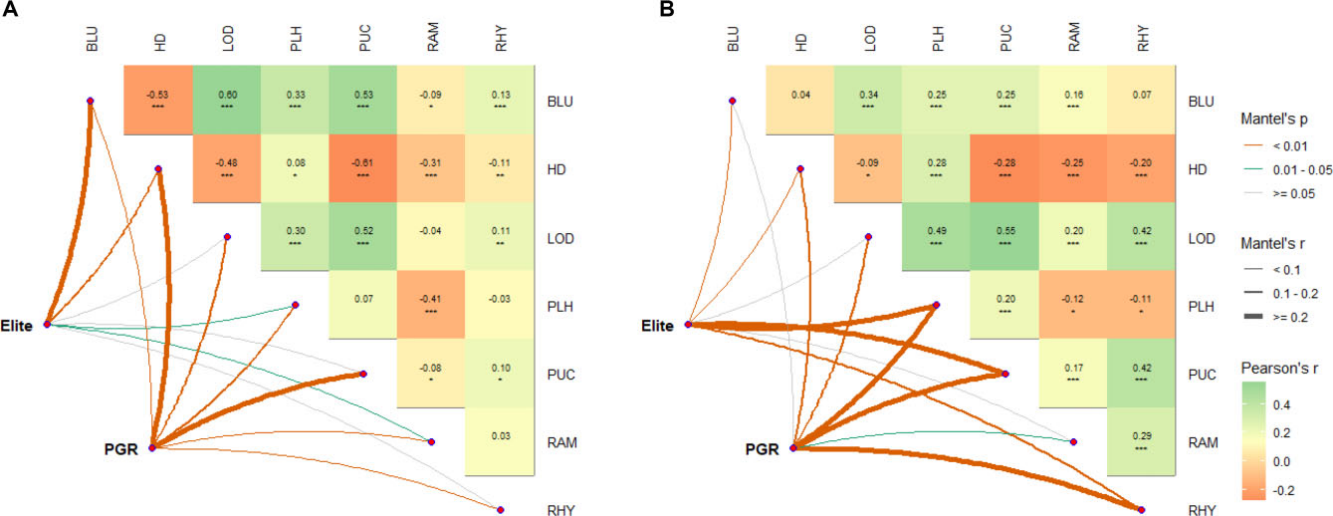
Pairwise correlations for the recorded traits and the Mantel tests between tested traits versus elite materials and plant genetic resources (PGR) for spring barley (A) and winter barley (B). The lines represent significant relationships, where the width of the line represents the Mantel *r* statistic value and the different colors of the lines represent different degrees of significance. The Pearson correlation coefficient between different traits is shown in the heatmap matrix. BLU: *Blumeria graminis hordei*; HD: heading date; LOD: lodging; PLH: plant height; PUC: *Puccinia hordei*; RAM: *Ramularia collo-cygni*; RHY: *Rhynchosporium commune*. *P < 0.05. **P < 0.01. *** *P* < 0.001.

Accurate mapping requires addressing the complexities inherent in genetic relatedness among individuals. In such way, especially when dealing with panels comprising both elite lines and PGRs, the intricate patterns of genetic relationship can pose significant challenges. Specially, when phenotype variation is influenced by genetic relatedness, it becomes crucial to differentiate between genuine associations and those resulting from shared genetic backgrounds. This complexity underscores the importance of robust methods to effectively uncover meaningful correlations and enhance the reliability of association mapping. Therefore, by minimizing genotype–phenotype covariance, we can reduce the risk of spurious associations [39]. The Mantel test is a widely used approach to examine the association between 2 matrices. The results revealed a moderate to low correlation between genetic distance and Euclidean phenotypic distance matrix, indicating a lack of strong association between phenotypic variation and genome-wide genetic differences (Fig. 6; Mantel’s *r* ranged from −0.02 to 0.29 in spring barley and from 0 to 0.32 in winter barley), which in turn is expected to increase the detection power in association mapping.

## Additional Files

**Supplementary Table S1**. List of 1,110 genotypes in this dataset.

**Supplementary Table S2**. The number and proportion of outliers identified for each trait.

giae121_Supplemental_File

giae121_Original_Submission

giae121_Revision_1

giae121_Response_to_Reviewer_Comments_Original_Submission

giae121_Reviewer_1_Report_Original_Submission Bo Song -- 11/3/2024

giae121_Reviewer_2_Report_Original_Submission Ana Casas -- 11/12/2024

## Abbreviations

BLU: *Blumeria graminis hordei*; BLUE: best linear unbiased estimations; HD: heading date; IPK: Institute of Plant Genetics and Crop Plant Research; LD: linkage disequilibrium; LOD: lodging; PCoA: principal coordinate analysis; PGR: plant genetic resources; PLH: plant height; PUC: *Puccinia hordei*; RAM: *Ramularia collo-cygni*; RHY: *Rhynchosporium commune*; SNP: single nucleotide polymorphism; WGS: whole genome sequencing.

## Data Availability

*Phenotypic records:* The raw phenotypic data described here as well as the ready-to-use phenotypic values (BLUEs) and the R script to import and curate the raw phenotypic data to compute heritability and BLUEs are available in the e!DAL-PGP Repository [40] and can be directly accessed here [41]. *Raw sequencing reads:* FASTQ files containing raw reads for 1,110 genotypes were submitted by [24] and deposited at the European Nucleotide Archive [42] under BioProjects PRJEB53924 (Illumina resequencing data). Sequenced genotypes are findable through their “SAMEA” IDs. The integrated Elite and PGR “SAMEA” BioSample IDs connected with plant material passports, passport data sources, SSD, and IPK genebank DOIs are listed in Supplementary Table S1. *SNP markers:* Variant calling results based on read mapping against the reference sequence of MorexV3 were stored as Variant Call Format (VCF). All the VCF files are located at the European Nucleotide Archive under the project number PRJEB80159. The script for filtering VCF files, imputation, admixture process, mantel test, and cross-validation is accessible at https://github.com/yzh1023/data-publication.git [43]. Other data further supporting this work are openly available in the *GigaScience* repository, GigaDB [44].
