# Supplementary material for: High-quality phenotypic and genotypic dataset of barley genebank core collection to unlock untapped genetic diversity
Source: Gigascience. 2025 Feb 11;14:giae121. doi: 10.1093/gigascience/giae121 (PMC11811526; doi:10.1093/gigascience/giae121)
Supplement: giae121_Original_Submission [file giae121_original_submission.pdf]

GigaScience

From genebank to field: enhancing barley breeding with precision phenotyping and genotyping

--Manuscript Draft--

**Manuscript Number:**	GIGA-D-24-00417
**Full Title:**	From genebank to field: enhancing barley breeding with precision phenotyping and genotyping
**Article Type:**	Data Note
**Funding Information:**	Bundesministerium für Ernährung und Landwirtschaft (031B0190A) — Not applicable<br>Bundesministerium für Ernährung und Landwirtschaft (031B0884A) — Not applicable

Abstract:

Background

Genebanks around the globe serve as valuable repositories of genetic diversity, offering not only access to a broad spectrum of plant material but also critical resources for enhancing crop resilience, advancing scientific research, and supporting global food security. To this end, traditional genebanks are evolving into bio-digital resource centres where the integration of phenotypic and genotypic data for accessions can drive more informed decision-making, optimize resource allocation, and unlock new opportunities for plant breeding and research. However, the curation and availability of interoperable phenotypic and genotypic data for genebank accessions is still in its infancy and represents an obstacle to rapid scientific discoveries in this field. Therefore, effectively promoting FAIR, i.e. findable, accessible, interoperable, and reusable, access to these data is vital for maximizing the potential of genebanks and driving progress in agricultural innovation.

Findings

Here we provide whole genome sequencing data of 812 barley (Hordeum vulgare L.) plant genetic resources (PGRs) and 298 European elite materials released between 1949 and 2021, as well as the phenotypic data for four disease resistance traits and three agronomic traits. The robustness of the investigated traits and the interoperability of genomic and phenotypic data were assessed in the current publication, aiming to make this panel publicly available as a resource for future genetic research in barley.

Conclusions

This high-quality phenotypic data, combined with the genotypic data, provides a valuable and unique training dataset for the development of novel genotype-based predictions and mapping methods for donor selection.

**Corresponding Author:**	Samira El Hanafi, PhD<br>Leibniz-Institut für Pflanzengenetik und Kulturpflanzenforschung Gatersleben: Leibniz-Institut fur Pflanzengenetik und Kulturpflanzenforschung (IPK)<br>Gatersleben, GERMANY
**Corresponding Author Secondary Information:**	
**Corresponding Author's Institution:**	Leibniz-Institut für Pflanzengenetik und Kulturpflanzenforschung Gatersleben: Leibniz-Institut fur Pflanzengenetik und Kulturpflanzenforschung (IPK)
**Corresponding Author's Secondary Institution:**	
**First Author:**	Zhihui Yuan
**First Author Secondary Information:**	

Order of Authors:	Zhihui Yuan
	Maximilian Rembe
	Martin Mascher
	Nils Stein
	Axel Himmelbach
	Murukarthick Jayakodi
	Andreas Börner
	Klaus Oldach
	Ahmed Jahoor
	Jens Due Jensen
	Julia Rudloff
	Viktoria-Elisabeth Dohrendorf
	Luisa Pauline Kuhfus
	Emmanuelle Dyrszka
	Matthieu Conte
	Frederik Hinz
	Salim Trouchaud
	Jochen C. Reif
	Samira El Hanafi
Order of Authors Secondary Information:	
Additional Information:	
Question	Response
Are you submitting this manuscript to a special series or article collection?	No
**Experimental design and statistics**<br><br>Full details of the experimental design and statistical methods used should be given in the Methods section, as detailed in our Minimum Standards Reporting Checklist. Information essential to interpreting the data presented should be made available in the figure legends.<br><br>Have you included all the information requested in your manuscript?	Yes
**Resources**<br><br>A description of all resources used, including antibodies, cell lines, animals	Yes

and software tools, with enough information to allow them to be uniquely identified, should be included in the Methods section. Authors are strongly encouraged to cite Research Resource Identifiers (RRIDs) for antibodies, model organisms and tools, where possible.<br><br>Have you included the information requested as detailed in our Minimum Standards Reporting Checklist?	
**Availability of data and materials**<br><br>All datasets and code on which the conclusions of the paper rely must be either included in your submission or deposited in publicly available repositories (where available and ethically appropriate), referencing such data using a unique identifier in the references and in the "Availability of Data and Materials" section of your manuscript.<br><br>Have you have met the above requirement as detailed in our Minimum Standards Reporting Checklist?	Yes

Manuscript

Click here to access/download;Manuscript;From genebank to field-Yuan.et.al-20240926.docx

From genebank to field: enhancing barley breeding with precision phenotyping and genotyping

Zhihui Yuan[1], Maximilian Rembe[1,2], Martin Mascher[1,3], Nils Stein[1,4], Axel Himmelbach[1], Murukarthick Jayakodi[1], Andreas Börner[1], Klaus Oldach[5], Ahmed Jahoor[6], Jens Due Jensen[6], Julia Rudloff[7], Viktoria-Elisabeth Dohrendorf[8], Luisa Pauline Kuhfus[9], Emmanuelle Dyrszka[9], Matthieu Conte[9], Frederik Hinz[10], Salim Trouchaud[11], Jochen C. Reif[1], Samira El Hanafi[1]

[1]Leibniz Institute of Plant Genetics and Crop Plant Research (IPK) Gatersleben, Seeland, Germany

[2]KWS SAAT SE & Co. KGaA, Grimsehlstr. 31, 37574 Einbeck, Germany

[3]German Centre for Integrative Biodiversity Research (iDiv) Halle-Jena-Leipzig, Leipzig, Germany

[4]Crop Plant Genetics, Institute of Agricultural and Nutritional Sciences, Martin-Luther-University of Halle-Wittenberg, Halle (Saale), Germany

[5]KWS LOCHOW GmbH, Ferdinand-von-Lochow-Str. 5, 29303 Bergen, Germany

[6]Nordic Seed Germany GmbH, Kirchhorster Str. 16 31688 Nienstädt, Germany

[7]Limagrain GmbH, Salderstr. 4, 31226 Peine-Rosenthal, Germany

[8]Nordsaat Saatzucht GmbH, Zuchtstation Gudow, Hofweg 8, D-23899 Gudow, Germany

[9]Syngenta France SAS, 12 Chemin de l'hobit, B.P. 27, 31790, Saint-Sauveur, France

[10]Saatzucht Bauer GmbH & CO.KG, Landshuter Straße 3a, 93083 Obertraubling, Germany

[11]Secobra Saatzucht GmbH, Feldkirchen 3, 85368 Moosburg an der Isar, Germany

corresponding author: Samira El Hanafi (hanafi@ipk-gatersleben.de)

Abstract

Background: Genebanks around the globe serve as valuable repositories of genetic diversity, offering not only access to a broad spectrum of plant material but also critical resources for enhancing crop resilience, advancing scientific research, and supporting global food security. To this end, traditional genebanks are evolving into bio-digital resource centres where the integration of phenotypic and genotypic data for accessions can drive more informed decision-

making, optimize resource allocation, and unlock new opportunities for plant breeding and research. However, the curation and availability of interoperable phenotypic and genotypic data for genebank accessions is still in its infancy and represents an obstacle to rapid scientific discoveries in this field. Therefore, effectively promoting FAIR, i.e. findable, accessible, interoperable, and reusable, access to these data is vital for maximizing the potential of genebanks and driving progress in agricultural innovation.

Findings: Here we provide whole genome sequencing data of 812 barley (*Hordeum vulgare* L.) plant genetic resources (PGRs) and 298 European elite materials released between 1949 and 2021, as well as the phenotypic data for four disease resistance traits and three agronomic traits. The robustness of the investigated traits and the interoperability of genomic and phenotypic data were assessed in the current publication, aiming to make this panel publicly available as a resource for future genetic research in barley.

Conclusions: This high-quality phenotypic data, combined with the genotypic data, provides a valuable and unique training dataset for the development of novel genotype-based predictions and mapping methods for donor selection.

Keywords

Barley; plant genetic resources; elite; whole genome resequencing; disease resistance; agronomic traits

Data Description

Context

Successful plant breeding programs rely on balanced efforts between short-term goals to develop competitive cultivars and the maintenance of a broad genetic pool to guarantee long-term progress. In practice, the development of new varieties has been predominantly derived by recycling existing elite lines, leading to important genetic improvement but also reduction in the genetic diversity of elite germplasm. This could impede breeding of potential new varieties capable of addressing and responding constraints related to climate change, agronomical threads, and meeting the escalating social demands [1]. To overcome these limitations,

leveraging genetic diversity harboured within plant genetic resources (PGR) has been frequently suggested [2]. PGRs provide a valuable reservoir of untapped genetic potential that can be utilized to develop varieties with improved yield [3] and end-use quality, and enhanced resistance to both biotic and abiotic stresses, such as diseases [4], pests [5], waterlogging [6], salinity [7], and drought [6,8].

As the most cost-effective *ex situ* conservation strategy, genebanks worldwide are committed to maintaining PGRs, which hold a diverse gene pool encompassing all the alleles of various genes, including those from wild species, landraces, and breeding stocks. However, although enormous efforts that have been made in conserving germplasm [9], it is estimated that less than 1% of these resources preserved in genebanks have been used in crop improvement [10]. The great challenge for breeders and scientists lies in finding useful barley PGRs among entire genebank collections that are comprised of thousands of accessions with complex patterns of genetic diversity [11]. Therefore, core collections were proposed as a strategy to streamline operational processes and mitigate costs, thereby facilitating more precise and effective research and breeding initiatives. Over the past decades, this approach has become even more attractive thanks to recent technological advancements, which have markedly reduced the costs of genotyping, and led to dramatic improvements in read length, sequencing chemistry, instrumentation, and throughput [12]. As a result, generating large-scale sequencing and genotyping datasets for entire genebank collections is now feasible. This has greatly expanded the scope of genotyping efforts and underpinned the effective selection of core collections that maximize genetic diversity [13]. These advancements provide powerful tools to efficiently harness PGRs, enabling the identification of valuable and favourable genes. This has streamlined their incorporation into crop improvement efforts, ultimately speeding up the development of new and improved varieties.

Moreover, the strategic deployment of core collections becomes even more compelling when combined with modern elite material [14,15], which served as a reference panel to define favourable alleles/genes that are absent in the elite panel. This integrated approach is essential for enhancing polygenic traits and, hence, achieving informed pre-breeding decisions. To put

this into practice, we have selected a barley core collection [16] from the German Federal *ex situ* Genebank for Agriculture and Horticultural Crops at the Leibniz Institute of Plant Genetics and Crop Plant Research (IPK) and combined it with a set of European elite material. The whole population was phenotyped in multi-environmental trials for three agronomically important traits: plant height (PLH), heading date (HD), lodging (LOD), and four disease traits: *Puccinia hordei* (PUC), *Blumeria graminis hordei* (BLU), *Ramularia collo-cygni* (RAM), and *Rhynchosporium commune* (RHY). Whole genome sequencing (WGS) was also carried out for the entire population. Coupled with extensive and high-quality phenotypic data, the systematic use of whole genome sequencing data could provide valuable insights into genetic diversity and potential breeding opportunities. Our recent findings using genome-wide association analyses highlighted the value of these data in selecting donors with potentially novel favourable genes [13].

The data presented here can be further extended with additional PGRs and/or elite materials by using the same environment parameters, and can be integrated with alternative approaches to enhance the use of germplasm collections, such as novel genotype-based predictions and the development of new association mapping methods. With the developing public access resources to enable next generations of scientists spend less time on generating and curing data, the insights derived from our data may significantly accelerate advancements in genomic research and breeding programs, driving improvement and fostering future collaboration and resource sharing.

Methods

Barley material and field trials

To capture a broad spectrum of geographic origins and wide genetic diversity, we selected 812 PGRs which include 288 spring type (PGR_Spring) and 524 winter type (PGR_Winter), originated from 57 countries spanning 5 continents. Based on their performance during seed regeneration, these PGRs were thoughtfully selected from a previously described barley core 1000 collection [16], as a representative subset of the entire 21,405 barley accessions available at the IPK genebank [17], based on their performance during seed regeneration. Additionally,

we incorporated 298 elite lines, including ten local checks, which consist of 128 spring type (Elite_Spring) and 170 winter type (Elite_Winter). These elites were exclusively selected from the European registered varieties and were available through seed market, showcasing the breeding process over time 1949 to 2021. The study initially included 87 additional genotypes which were later excluded from certain analyses due to incomplete phenotypic or genotypic data. To maintain the integrity of the dataset and facilitate accurate adjustments for experimental design effects, we retained all relevant data, including instances of missing information.

Field trials were conducted over three consecutive years (2020, 2021, and 2022) across eight locations in Germany: KWS-L/Prosselsheim (49°51'15.6"N, 10°06'04.1"E); Nordic Seed/Nienstädt (52°17'35.52"N, 9°08'57.156"E); Saatzucht Bauer/Riekofen (48°54'55.98"N, 12°21'21.744"E); Limagrain/Peine-Rosenthal (52°18'09.828"N, 10°10'28.488"E); Nordsaat/Gudow (53°33'28.0"N, 10°47'50.5"E); Syngenta/Bad Salzuflen (52°04'21.576"N, 8°41'55.86"E); Secobra-LEM/Lemgo (52°00'41.6"N, 8°52'22.7"E); Secobra-FK/Moosburg (48°28'46.8"N, 11°54'32.6"E). The trials were sown following a generalized alpha lattice design in two-row observation plots (1 m^2). Ten checks were used across years and locations. Each unique combination of year and location was considered as distinct environment.

Phenotyping

The whole population was phenotyped for three agronomy traits: heading date (HD) measured in days from January 1st for winter type and from the sowing date onward for the spring type; plant height (PLH) measured from the soil surface to the tip of spike in cm (excluding awns); and lodging (LOD) rated on a 1-9 scale (with a higher score indicating severe lodging). Additionally, four disease traits including *Puccinia hordei* (PUC), *Blumeria graminis hordei* (BLU), *Ramularia collo-cygni* (RAM), and *Rhychnosporium commune* (RHY) were evaluated under natural infection conditions. The disease severities were scored using an ordinal scale from 1 (fully resistant) to 9 (fully susceptible) following the guidelines of the German Federal Plant Variety Office [18].

Phenotypic data analyses

A linear mixed model using restricted maximum likelihood (REML) method [19] was used for data analyses across environments for spring and winter barley separately. Phenotypic data was corrected for outliers following the method of Tukey and Anscombe [20]. The residuals were extracted then normalized to flag the outliers according to a predefined significance threshold of p-value < 0.01. Variance components and best linear unbiased estimations (BLUEs) of each genotype were computed from the oulier-corrected data following model (1):

$$y_{ijkm} = \mu + E_m + g_i + g_i \times E_m + E_m{:}r_j : b_k + e_{ijkm}, \quad (1)$$

where y_{ijkm} denoted the vector of phenotypic values for i^{th} genotype (g) tested in k^{th} bock (b) nested in j^{th} replication (r) in m^{th} environment (E), μ was the common mean, and e denoted the error term of the model. We assumed that all random effects followed an independent normal distribution with different variance components. In the model (1), all terms except μ and g_i were considered random for deriving the BLUEs across environments, whereas all terms except μ were modelled as random to estimate variance component for deriving heritability following model (2):

$$heritability = \frac{\sigma_g^2}{\sigma_g^2 + \frac{\sigma_{g\times E}^2}{\overline{n_E}} + \frac{\sigma_e^2}{\overline{n_R}}}, \quad (2)$$

where σ_g^2 denoted the genotypic variance, $\sigma_{g\times E}^2$ denoted the interaction between genotype and environment, σ_e^2 denoted the residual variance, and $\overline{n_R}$ denoted the average number of replications per genotype, $\overline{n_E}$ denotes the average number of environments in which the genotypes were evaluated. ASReml-R [21] was employed for all mixed linear models that were applied in the phenotypic analysis.

Whole genome shotgun sequencing

Whole genome sequencing (WGS) of the 1,110 genotypes (812 PGRs and 298 elite lines) was performed at IPK Gatersleben. High molecular weight (HMW) DNA was extracted from the leaves (8g) of greenhouse-grown (21°C/18°C day/night temperature) 7-days old seedlings following previously established protocol [22]. The Illumina Nextera libraries were prepared and sequenced using the Illumina NovaSeq 6000 platform [23]. Raw sequencing reads were trimmed using cutadapt [24] (version 3.3) and aligned to MorexV3 reference genome [25] using

Minimap2 [26] (version 2.20). The resultant alignment records were sorted with Novosort (V3.09.01; http://www.novocraft.com). Finally, a total of 149,380,812 single-nucleotide polymorphisms (SNPs) for the 1,110 genotypes were initially outputted by BCFtools [27] (version 1.9).

Quality control for SNP data

The resulting raw genotypic data was used to extract the corresponding datasets of the four sub-groups. Only bi-allelic SNPs with minor allele frequency > 0.05 and missing rate < 0.1 were retained by PLINK [28] (version 1.9) on the four sub-groups basis. These meticulous steps yielded datasets comprising 17,759,260 SNPs for Elite_Spring, 26,903,811 for Elite_Winter, 54,934,336 for PGR_Spring, and 46,434,685 for PGR_Winter.

The resulting filtered genotypic data was used as input to impute missing values using Beagle [29] (version 5.2). Subsequently, an r^2 cutoff of 0.2 was set to prune markers by PLINK (version 1.9) with a sliding window size of 50 kb, and a step size of 10 kb. The final number of SNPs available differed in the four sub-groups due to the aforementioned process: 710,855 of Elite_Spring, 945,074 of Elite_Winter, 2,321,327 of PGR_Spring, and 1,775,972 of PGR_Winter. For each tested SNP, homozygous for the most frequent allele, heterozygous, and homozygous for the alternative allele were coded as 0, 1, and 2 by PLINK (version 1.9), respectively.

Population structure

Subsequently, the aforementioned post-quality-control markers were used to investigate the population structure within and across spring and winter barley accessions using principal coordinate analysis (PCoA) based on pairwise Rogers' distance [30]. PCoA was performed using the R package ape [31] (version v5.7-1).

Additionally, linkage disequilibrium (LD) analyses of the four sub-groups was carried out separately by determining the pairwise squared allele-frequency correlations (r^2) between markers [32] and then combined to estimate LD decay across the entire genome. A decay curve was fitted for each sub-group using nonlinear regression of pairwise r^2 against the distance (Mb)

between the markers. LD within a specific physical distance of 2 Mb was calculated and visualized using PopLDdecay [33] (version 3.40).

Mantel correlation

Following the imputation process, we used PLINK (version 1.9) to construct a genetic relationship matrix. To further explore the association between phenotypic variation and population structure, the correlation between genetic relationship matrix and the absolute trait differences (Euclidean distance matrix) in each sub-group was tested using a Mantel test [34] implemented in the vegan [35] R package (v2.6-4), and visualized by linkET [36] R package (v0.0.7.4), 999 permutations were used to evaluate the significance of the test.

Data Validation and quality control

High heritability estimation highlights the robustness of the phenotypic data

The quality and reliability of the phenotypic data was rigorously assessed by estimating the heritability of the evaluated traits. After outlier correction, the heritability estimates for most traits were generally high, exceeding 0.5 (Fig. 1A). Notable exceptions included RHY in the spring population (h^2 = 0.05), and RAM (h^2 = 2E-06) in the winter population. Variance components analysis revealed that the environments accounts for the largest proportion of the total variation, while genotype and genotype × environments interaction were less pronounced, with the exception of LOD and RHY in both spring and winter population, as well as PUC in winter population (Fig. 1B).

The resulting BLUEs showed normal distribution for most disease traits (Fig. 2). However, RHY showed left skew in both spring and winter population, while BLU and PUC displayed left skew in elite population for both spring and winter type. The left skew of RHY suggests low disease pressure across three years and, hence, resulting in a small proportion of susceptible genotypes. And the left skew of elite population of BLU and PUC suggests that PGRs tend to be more susceptible than the elite materials for the two diseases. For agronomic traits (Fig. 3), PGR population showed normal distribution, while elite lines showed normal distribution in HD and PLH only in winter population.

Furthermore, several significant correlations were observed between the evaluated traits (Fig. 4). For pairing of agronomic and disease traits, it was observed that HD was negatively correlated with all the disease traits, except for BLU in winter barley population. Those observations suggest a strategic plant response given that delayed heading allows plants to evade disease infection through spatial or temporal adjustments. Moreover, LOD was positively correlated with all the disease traits, except for RAM in spring barley population. PLH was positively correlated with BLU and PUC while negatively correlated with RAM and RHY.

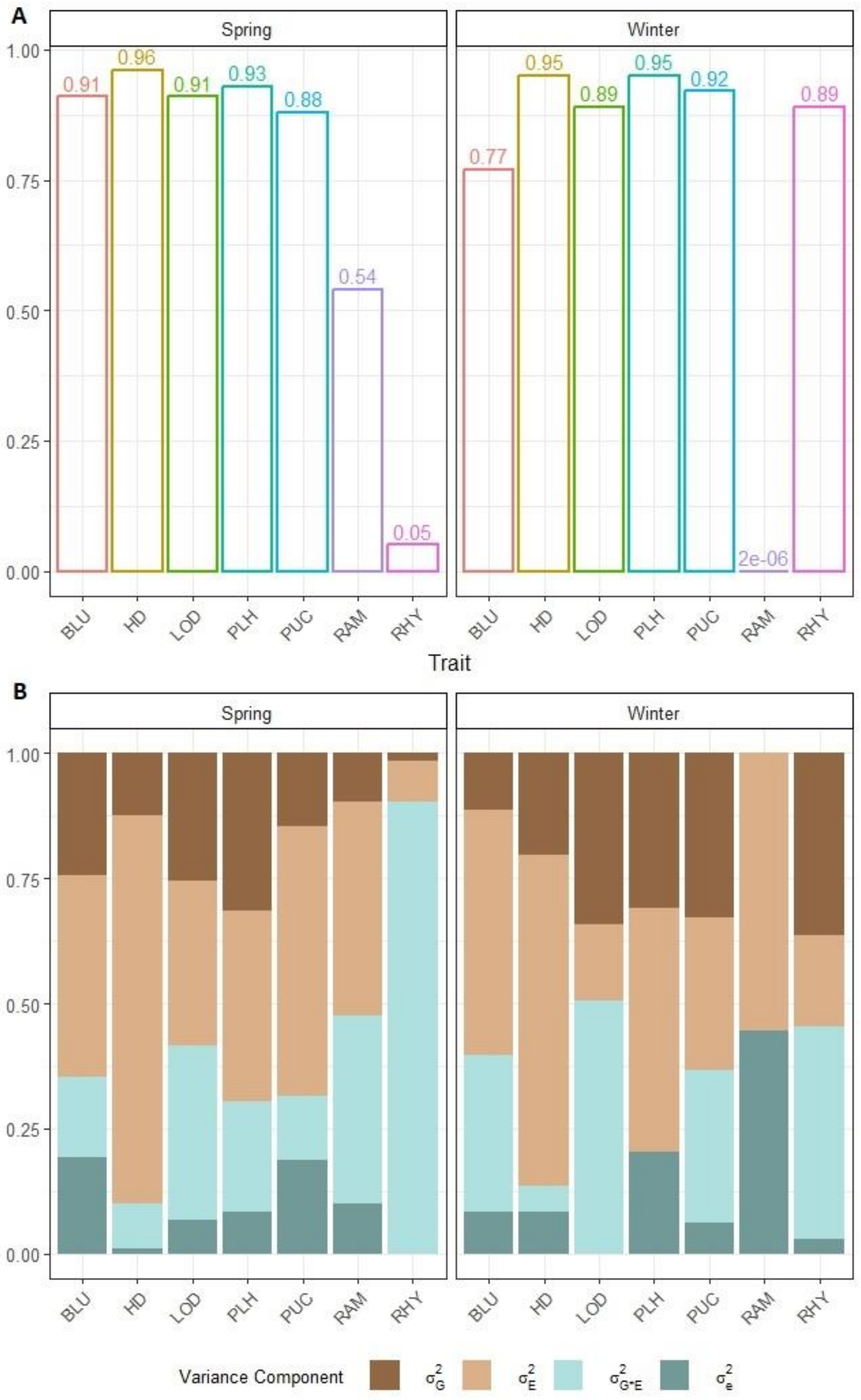

Figure 1: Heritability (A) and percentages of the different variance components (B) for the seven traits considered in this study. BLU: *Blumeria graminis hordei*; PUC: *Puccinia hordei*; RHY: *Rhynchosporium commune*; RAM: *Ramularia collo-cygni*; HD: heading date; PLH: plant height; LOD: lodging; σ^2_G: genotypic variance; σ^2_{G*E}: variance due to genotype by environment interaction; σ^2_E: variance due to environment; σ^2_e: residual.

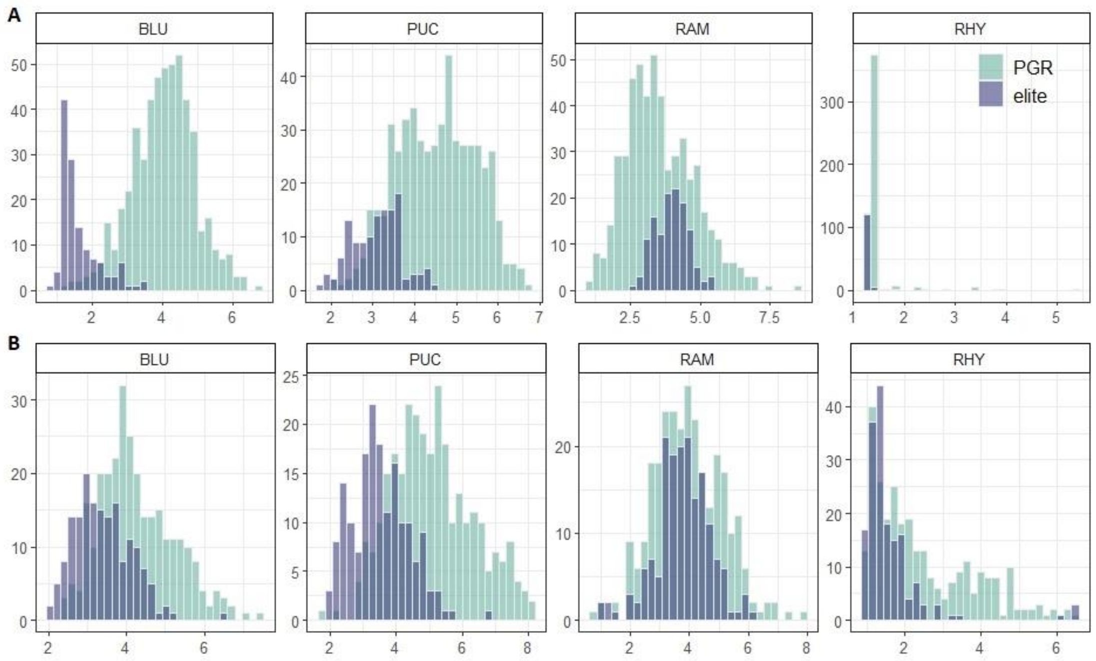

Figure 2: Histogram showing the phenotypic distribution for four diseases traits for spring (A) and winter (B) population. BLU: *Blumeria graminis hordei*; PUC: *Puccinia hordei*; RHY: *Rhynchosporium commune*; RAM: *Ramularia collo-cygni*.

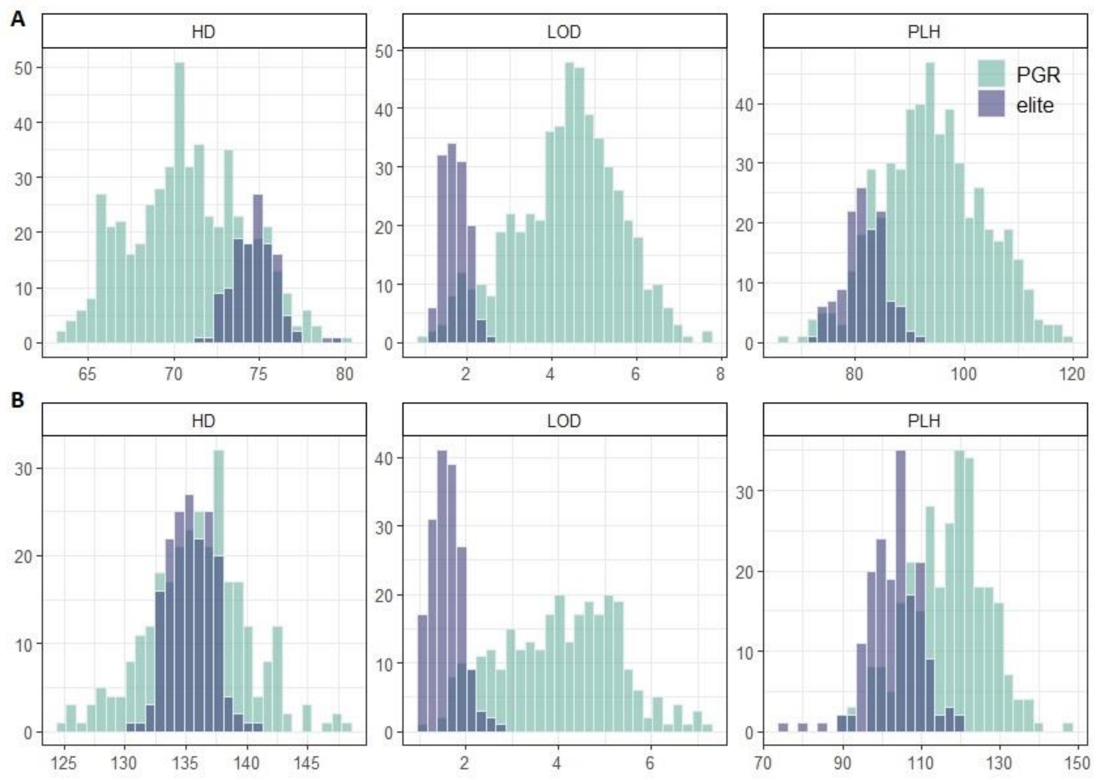

Figure 3: Histogram showing the phenotypic distribution for three agronomic traits for spring (A) and winter (B) population. HD: heading date; PLH: plant height; LOD: lodging.

Whole genome sequencing data showed high genetic diversity and high marker densities

Whole genome sequencing (WGS) data of the 1,110 genotypes showed an average coverage of 4.7x with a range spanning from 0.5x to 22.6x across all samples with mapping rate from 94% to 99%, providing a solid foundation for downstream genetic analyses and ensuring comprehensive representation of the genomic information across the diverse set of genotypes.

Building on this comprehensive genomic dataset, we performed PCoA to assess the genetic diversity among the spring and winter barley population as reported in our companion study [37]. The first two coordinates explained together 11.66% and 11.25% of the spring and winter population, respectively. As anticipated, the inclusion of PGRs significantly broadened the genetic diversity compared to the elite materials. Notably, the elite spring population formed a tight, cohesive cluster indicating less genetic diversity, while the elite winter population exhibited a more dispersed pattern reflecting greater genetic variability.

For the intra-chromosomal decay of LD (r^2), PGR is faster in both spring and winter population as compared to elite materials. The slower LD decay in elite population may be due to genetic bottlenecks and/or high selection pressures that produce specific linkage between alleles that control specific phenotypes.

Mantel test results indicate a high detection power in association mapping

Accurate mapping requires addressing the complexities inherent in genetic relatedness among individuals. In such way, especially when dealing with panels comprising both elite lines and PGRs, the intricate patterns of genetic relationship can pose significant challenges. Specially, when phenotype variation is influenced by genetic relatedness, it becomes crucial to differentiate between genuine associations and those resulting from shared genetic backgrounds. This complexity underscores the importance of robust methods, to effectively uncover meaningful correlations and enhance the reliability of association mapping. Therefore, by minimizing genotype-phenotype covariance, we can reduce the risk of spurious associations [38]. Mantel test is a widely used approach to examine the association between two matrices. The results revealed a moderate to low correlation between genetic distance and Euclidean phenotypic distance matrix, indicating a lack of strong association between phenotypic variation and genome-wide genetic differences (Fig. 4; Mantel's r in spring barley ranged from -0.02 to

0.29, and from 0 to 0.32 in winter barley), which in turn expected to increase the detection power in association mapping.

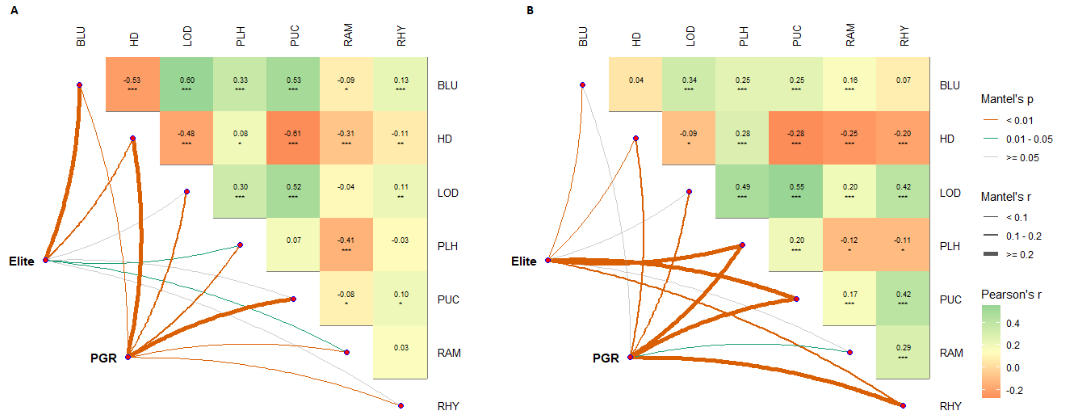

Figure 4: Pairwise correlations for the recorded traits, and the Mantel tests between tested traits vs. elite materials and plant genetic resources (PGR) for spring barley (A) and winter barley (B). The lines represent significant relationships, where the width of the line represents the Mantel r statistic value and the different colours of the lines represent different degrees of significance. The Pearson correlation coefficient between different traits is shown in the heat map matrix. BLU: *Blumeria graminis hordei*; PUC: *Puccinia hordei*; RHY: *Rhynchosporium commune*; RAM: *Ramularia collo-cygni*; HD: heading date; PLH: plant height; LOD: lodging; *** p < 0.001, ** p < 0.01, * p < 0.05.

Data Availability

Phenotypic records: The raw phenotypic data described here as well as the ready-to-use phenotypic values (BLUEs), and the R script to import and curate the raw phenotypic data to compute heritability and BLUEs are available in the e!DAL-PGP Repository [39] and can be directly accessed here [40].

Raw sequencing reads: FASTQ files containing raw reads for 1,110 genotypes were submitted by [23], and deposited at the European Nucleotide Archive [41] under BioProjects PRJEB53924 (Illumina resequencing data). Sequenced genotypes are findable through their 'SAMEA' IDs. The integrated Elite and PGR 'SAMEA' BioSample IDs connected with plant material passports, passport data sources, SSD- and IPK genebank DOIs was listed in Supplementary Table S1.

SNP markers: variant calling results based on read mapping against the reference sequence of MorexV3 were stored as Variant Call Format (VCF). All the VCF files are located at the European Nucleotide Archive under the project number PRJEB80159.

Abbreviations

BLU: *Blumeria graminis hordei*; BLUE: best linear unbiased estimations; HD: heading date; IPK: Institute of Plant Genetics and Crop Plant Research; LD: linkage disequilibrium; LOD: lodging; PCoA: principal coordinate analysis; PGR: plant genetic resources; PLH: plant height; PUC: *Puccinia hordei*; RAM: *Ramularia collo-cygni*; RHY: *Rhynchosporium commune*; SNP: single-nucleotide polymorphisms; WGS: whole genome sequencing.

Author's Contributions

KO, AJ, JDJ, JR, VD, LPK, ED, MC, FH, and ST: cultivation and provision of phenotypic data of all spring and winter barleys over 3 years in one to two locations; ZY: genotypic data analyses and curation; MR and SEH: phenotypic data analysis; MM, MJ, AH, and NS: generated and processed the genomic data; NS, MM, AB, SEH, and JCR: edited and revised the manuscript; AB: developed the core 1000 population; JCR, NS, SEH, and ZY: designed the study; ZY and SEH: wrote the paper. All authors read and approved the final manuscript.

Funding

This research work is funded by German Ministry of Food and Agriculture under the project Structural genome variation, haplotype diversity and the barley pan-genome - Exploring structural genome diversity for barley breeding (SHAPE) phase 1 and 2 (BMBF FKZ 031B0190A; 031B0884A).

Competing Interests

No conflict of interest declared.

Acknowledgement

We are grateful for the technical assistance of Mary Ziems and Annette Marlow for providing seeds of plant material. Susanne König, and Ines Walde for technical assistance during

sequencing data production, as well as Anne Fiebig for support with data management and submission to repositories.

References

1. Ellegren H, Galtier N. Determinants of genetic diversity. *Nat Rev Genet.* 2016;17:422–433.

2. Halewood M, Chiurugwi T, Sackville Hamilton R *et al.* Plant genetic resources for food and agriculture: opportunities and challenges emerging from the science and information technology revolution. *New Phytologist.* 2018;217:1407–1419.

3. Dillon SL, Shapter FM, Henry RJ *et al.* Domestication to Crop Improvement: Genetic Resources for *Sorghum* and *Saccharum* (Andropogoneae). *Annals of Botany* 2007;100:975–989.

4. Deng Y, Ning Y, Yang D *et al.* Molecular Basis of Disease Resistance and Perspectives on Breeding Strategies for Resistance Improvement in Crops. *Molecular Plant* 2020;13:1402–1419.

5. Radchenko EE, Abdullaev RA, Anisimova IN. Genetic Resources of Cereal Crops for Aphid Resistance. *Plants* 2022;11:1490.

6. Valliyodan B, Ye H, Song L *et al.* Genetic diversity and genomic strategies for improving drought and waterlogging tolerance in soybeans. *J Exp Bot.* 2016;68:1835–1849.

7. Razzaq A, Saleem F, Wani SH *et al.* De-novo Domestication for Improving Salt Tolerance in Crops. *Front Plant Sci.* 2021;12:681367.

8. Missanga JS, Venkataramana PB, Ndakidemi PA. Recent developments in *Lablab purpureus* genomics: A focus on drought stress tolerance and use of genomic resources to develop stress-resilient varieties. *Legume Science* 2021;3:e99.

9. Wambugu PW, Ndjiondjop M-N, Henry RJ. Role of genomics in promoting the utilization of plant genetic resources in genebanks. *Briefings in Functional Genomics* 2018;17:198–206.

10. Sharma S, Upadhyaya HD, Varshney RK *et al.* Pre-breeding for diversification of primary gene pool and genetic enhancement of grain legumes. *Front Plant Sci.* 2013;4:309.

11. Odong TL, Jansen J, Van Eeuwijk FA *et al.* Quality of core collections for effective utilisation of genetic resources review, discussion and interpretation. *Theor Appl Genet.* 2013;126:289–305.

12. Salgotra RK, Chauhan BS. Genetic Diversity, Conservation, and Utilization of Plant Genetic Resources. *Genes* 2023;14:174.

13. El Hanafi S, Jiang Y, Kehel Z *et al.* Genomic predictions to leverage phenotypic data across genebanks. *Front Plant Sci.* 2023;14:1227656.

14. Cazenave X, Petit B, Lateur M *et al.* Combining genetic resources and elite material populations to improve the accuracy of genomic prediction in apple. *G3 Genes|Genomes|Genetics* 2022;12:jkab420.

15. Sehgal D, Vikram P, Sansaloni CP *et al.* Exploring and Mobilizing the Gene Bank Biodiversity for Wheat Improvement. *PLoS ONE* 2015;10:e0132112.

16. Milner SG, Jost M, Taketa S *et al.* Genebank genomics highlights the diversity of a global barley collection. *Nat Genet.* 2019;51:319–326.

17. Oppermann M, Weise S, Dittmann C *et al.* GBIS: the information system of the German Genebank. *Database* 2015;2015,bav021.

18. Bundessortenamt. Richtlinien für die Durchführung von landwirtschaftlichen Wertprüfungen und Sortenversuchen. 2000. https://www.bundessortenamt.de/bsa/sorten/sortenzulassung/richtlinien-fuer-die-durchfuehrung-von-landwirtschaftlichen-wertpruefungen-und-sortenversuchen.

19. Patterson HD, Thompson R. Recovery of inter-block information when block sizes are unequal. *Biometrika.* 1971;58:545–554.

20. Anscombe FJ, Tukey JW. The Examination and Analysis of Residuals. *Technometrics* 1963;5:141–60.

21. Butler DG, Cullis BR, Gilmour AR *et al.* ASReml estimates variance components under a general linear. 2023. https://asreml.kb.vsni.co.uk/wp-content/uploads/sites/3/2018/07/ASReml-Package.pdf

22. Dvorak J, McGuire PE, Cassidy B. Apparent sources of the A genomes of wheats inferred from polymorphism in abundance and restriction fragment length of repeated nucleotide sequences. *Genome* 1988;30:680–689.

23. Jayakodi M, Lu Q, Pidon H *et al.* Adaptive diversification through structural variation in barley. 2024. doi:10.1101/2024.02.14.580266.

24. Martin M. Cutadapt removes adapter sequences from high-throughput sequencing reads. *EMBnet.journal* 2011;17:10.

25. Mascher M, Wicker T, Jenkins J *et al.* Long-read sequence assembly: a technical evaluation in barley. *The Plant Cell* 2021;33:1888–1906.

26. Li H. Minimap2: pairwise alignment for nucleotide sequences. *Bioinformatics* 2018;34:3094–3100.

27. Li H. A statistical framework for SNP calling, mutation discovery, association mapping and population genetical parameter estimation from sequencing data. *Bioinformatics* 2011;27:2987–2993.

28. Purcell S, Neale B, Todd-Brown K *et al.* PLINK: A Tool Set for Whole-Genome Association and Population-Based Linkage Analyses. *The American Journal of Human Genetics* 2007;81:559–575.

29. Browning BL, Zhou Y, Browning SR. A One-Penny Imputed Genome from Next-Generation Reference Panels. *The American Journal of Human Genetics* 2018;103:338–348.

30. Rogers JS. Measures of genetic similarity and genetic distance. *Studies in genetics VII* 1972:145–153.

31. Paradis E, Schliep K. ape 5.0: an environment for modern phylogenetics and evolutionary analyses in R. Schwartz R (ed.). *Bioinformatics* 2019;35:526–528.

32. Hill WG, Robertson A. Linkage Disequilibrium in Finite Populations. *Theoretical and Applied Genetics* 1968;38:226–231.

33. Zhang C, Dong SS, Xu JY *et al.* PopLDdecay: A fast and effective tool for linkage disequilibrium decay analysis based on variant call format files. *Bioinformatics* 2019;35:1786–1788.

34. Mantel N. The detection of disease clustering and a generalized regression approach. *Cancer Res* 1967;27:209–220.

35. Oksanen J, Simpson GL, Blanchet FG *et al.* vegan: Community Ecology Package. 2022.

36. Huang H. LinkET: everything is linkable. *R package version 0.0.7.4* 2021;3.

37. Yuan Z, Rembe M, Mascher M *et al.* Capitalizing genebank core collections for rare and novel disease resistance loci to enhance barley resilience. *Journal of Experimental Botany* 2024:erae283.

38. Myles S, Peiffer J, Brown PJ *et al.* Association Mapping: Critical Considerations Shift from Genotyping to Experimental Design. *Plant Cell* 2009;21:2194–2202.

39. Arend D, Junker A, Scholz U *et al.* PGP repository: a plant phenomics and genomics data publication infrastructure. *Database* 2016;2016:baw033.

40. Yuan Z, El Hanafi S, Reif J. Diseases resistance and agronomic traits of 853 plant genetic resources and 344 European elite genotypes in multi-environments. 2024. doi:10.5447/IPK/2024/7.

41. Li W, Cowley A, Uludag M *et al.* The EMBL-EBI bioinformatics web and programmatic tools framework. *Nucleic Acids Res* 2015;43:W580–W584.

supplementary table S1

Click here to access/download
Supplementary Material
Supplementary Table-Yuan.et.al-20240926.xlsx
