# Supplementary material for: High-quality phenotypic and genotypic dataset of barley genebank core collection to unlock untapped genetic diversity
Source: Gigascience. 2025 Feb 11;14:giae121. doi: 10.1093/gigascience/giae121 (PMC11811526; doi:10.1093/gigascience/giae121)
Supplement: giae121_Revision_1 [file giae121_revision_1.pdf]

GigaScience

High-quality phenotypic and genotypic dataset of barley genebank core-collection to unlock untapped genetic diversity

--Manuscript Draft--

Manuscript Number:	GIGA-D-24-00417R1
Full Title:	High-quality phenotypic and genotypic dataset of barley genebank core-collection to unlock untapped genetic diversity
Article Type:	Data Note
Funding Information:	Bundesministerium für Ernährung und Landwirtschaft (031B0884A) — Not applicable<br>Bundesministerium für Ernährung und Landwirtschaft (031B0190A) — Not applicable
Abstract:	Background: Genebanks around the globe serve as valuable repositories of genetic diversity, offering not only access to a broad spectrum of plant material but also critical resources for enhancing crop resilience, advancing scientific research, and supporting global food security. To this end, traditional genebanks are evolving into bio-digital resource centres where the integration of phenotypic and genotypic data for accessions can drive more informed decision-making, optimize resource allocation, and unlock new opportunities for plant breeding and research. However, the curation and availability of interoperable phenotypic and genotypic data for genebank accessions is still in its infancy and represents an obstacle to rapid scientific discoveries in this field. Therefore, effectively promoting FAIR, i.e. findable, accessible, interoperable, and reusable, access to these data is vital for maximizing the potential of genebanks and driving progress in agricultural innovation.<br>Findings: Here we provide whole genome sequencing data of 812 barley (Hordeum vulgare L.) plant genetic resources (PGRs) and 298 European elite materials released between 1949 and 2021, as well as the phenotypic data for four disease resistance traits and three agronomic traits. The robustness of the investigated traits and the interoperability of genomic and phenotypic data were assessed in the current publication, aiming to make this panel publicly available as a resource for future genetic research in barley.<br>Conclusions: The data showed broad phenotypic variability and high association mapping potential, offering a key resource for identifying genebank donors with untapped genes to advance barley breeding while safeguarding genetic diversity.
Corresponding Author:	Samira El Hanafi, PhD<br>Leibniz-Institut für Pflanzengenetik und Kulturpflanzenforschung Gatersleben: Leibniz-Institut fur Pflanzengenetik und Kulturpflanzenforschung (IPK)<br>Gatersleben, GERMANY
Corresponding Author Secondary Information:	
Corresponding Author's Institution:	Leibniz-Institut für Pflanzengenetik und Kulturpflanzenforschung Gatersleben: Leibniz-Institut fur Pflanzengenetik und Kulturpflanzenforschung (IPK)
Corresponding Author's Secondary Institution:	
First Author:	Zhihui Yuan
First Author Secondary Information:	
Order of Authors:	Zhihui Yuan
	Maximilian Rembe
	Martin Mascher
	Nils Stein
	Axel Himmelbach
	Murukarthick Jayakodi

Powered by Editorial Manager® and ProduXion Manager® from Aries Systems Corporation

	Andreas Börner
	Klaus Oldach
	Ahmed Jahoor
	Jens Due Jensen
	Julia Rudloff
	Viktoria-Elisabeth Dohrendorf
	Luisa Pauline Kuhfus
	Emmanuelle Dyrszka
	Matthieu Conte
	Frederik Hinz
	Salim Trouchaud
	Jochen C. Reif
	Samira El Hanafi
**Order of Authors Secondary Information:**	
**Response to Reviewers:**	Manuscript GIGA-D-24-00417<br>Response to reviews<br><br>Dear Dr. Nogoy,<br>Thank you for considering our manuscript for publication in GigaScience. We appreciate the time and effort that you and the reviewers have taken to provide feedback on our manuscript, and we are grateful for the insightful comments and constructive suggestions. We have carefully addressed the points raised and incorporated them into the revised manuscript. The changes are highlighted in yellow in the revised version for easy reference. We have also made minor edits throughout the manuscript to improve readability and clarity. The adjustments do not alter the content or conclusions of the paper, but enhance the overall given information. We hope that these revisions substantially strengthen our manuscript and believe that it now meets the standards for publication in GigaScience.<br>Reviewer1 Comments to the Author:<br>This data note presents a comprehensive study on the integration of genebank resources with precision phenotyping and genotyping to enhance barley breeding. The authors have effectively highlighted the significance of genetic diversity in barley and the role of genebanks in supporting agricultural innovation. The topic is highly relevant to current challenges in agriculture, particularly in the context of food security and climate change. Although the study carries significant strengths making it qualified for publication in GigaScience but needs some reconsiderations to resolve some concerns issues before going to formal acceptance for publication and are given as under. The study has the potential to make a significant contribution to the field of barley breeding and genomics. With revisions addressing the identified areas for improvement, this note could be a strong candidate for publication.<br>Authors’ response: Thank you for your thorough review and valuable feedback. We appreciate the time and effort you have invested in evaluating our work. We have addressed each of your comments and suggestions in detail below. Changes have been made and highlighted accordingly in the manuscript.<br>As an article of "Data Note", the title is overstated. The title should focus more on the data itself.<br>Authors’ response: Thank you for your feedback. We appreciate your insight regarding the title of the manuscript. Based on the nature of a "Data Note" article, we have changed our title to “High-quality phenotypic and genotypic dataset of barley genebank core-collection to unlock untapped genetic diversity”.<br>Summarize the main conclusions drawn from the results succinctly in the abstract.<br>Authors’ response: We appreciate your suggestion. A brief conclusion has been included in the abstract. Please refer to line 41-43.<br>Try to add more background information on barley breeding and its challenges particularly emphasize the relevance of the research in the current agricultural

Powered by Editorial Manager® and ProduXion Manager® from Aries Systems Corporation

landscape.
Authors' response: Thank you for your valuable feedback. We appreciate your suggestion to provide more background information on barley breeding and its challenges. We have expanded the introduction to include a short discussion of barley breeding, highlighting its significance in global importance. Specifically, we outlined the key challenges faced by breeders, such as adapting barley to changing climatic conditions, improving disease resistance, and enhancing yield stability (please refer to lines 53-55 and lines 57-60).
Additionally, we emphasized the relevance of our research within the context of the current agricultural landscape, given the increasing demand for leveraging the genetic diversity harbored within plant genetic resources. We also discuss how advances in genomics and phenotyping, such as tools explored in our study, can contribute to addressing these challenges and improving the efficiency of barley breeding programs. We believe these additions help contextualize our research and demonstrate its importance in advancing barley breeding to meet future agricultural demands. Thank you again for your insightful suggestion.
State the specific objectives of the study more explicitly and in a separate paragraph and briefly outline the structure of the manuscript at the end of this section.
Authors' response: Thank you for your constructive suggestion. We have revised the context to include a distinct part that explicitly states the specific objectives of the study. This paragraph now clearly outlines our aims. Please refer to lines 91-96.
It is suggested to summarize/describe the environmental conditions under which the trials were conducted.
Authors' response: Thank you for your valuable feedback. We have added a detailed summary of the environmental conditions under which the trials were conducted. This section now describes the average temperature across three years for each location. Please refer to lines 123-136.
Can you please provide justification for the choice of traits and methods used in the study and elaborate on the phenotyping methods, including any specific protocols followed.
Authors' response: Thank you for your insightful comments. We appreciate the opportunity to provide further justification for the choice of traits and methods used in this study.
The agronomic traits (heading date, plant height, and lodging) were selected for their importance in barley adaptability, yield potential, and harvestability, while disease traits address key breeding challenges for durable resistance. Phenotyping followed standardized protocols: heading date as days to 50% heading from January 1st for winter type and from the sowing date for the spring type, plant height measured at maturity, and lodging scored visually. The four disease traits [Puccinia hordei (PUC), Blumeria graminis hordei (BLU), Ramularia collo-cygni (RAM), and Rhychnosporium commune (RHY)] were included because these diseases are prevalent in the study's geographic region and are known to significantly affect crop health and yield. Disease resistance was assessed under field conditions to ensure robust and reproducible data, directly linking genebank materials to breeding applications.
We have integrated your comment in the revised manuscript to enhance transparency and to ensure that the methods used are well-justified. Please refer to line 140-148.
Provide some details on the alpha lattice design used in the trials and try to add a description of the statistical methods used for data analysis along with significance level of results obtained.
Authors' response: Thank you for your valuable feedback. We appreciate your suggestion to provide more details on the alpha lattice design and statistical methods used in the trials. A detailed description of the alpha lattice design employed in our trials is available in the reference [40]. For clarity, we have included a brief summary of the design in the revised manuscript, ensuring that key elements (please refer to lines 133-137). Regarding the statistical methods, we have included a detailed description of the analyses performed in the "Phenotypic data analyses" section, please refer to lines 149-168.
Clarify the criteria used for selecting SNPs for analysis besides providing more details on the imputation methods used for missing data.
Authors' response: We appreciate your attention to detail. To address this, we have added a sentence in the manuscript specifying the criteria for SNP selection. These criteria include minor allele frequency > 0.05 and missing rate < 0.1, and an r2 cutoff of 0.2 was set to prune markers.
Regarding the imputation methods, Beagle was chosen due to its reliability in phasing

Powered by Editorial Manager® and ProduXion Manager® from Aries Systems Corporation

and imputing genotypes, leveraging linkage disequilibrium to infer missing data accurately. The process involved phasing the genotypes and imputing missing data. Please refer to lines 186-188.

Given the limitations of Principal Coordinates Analysis (PCoA) in addressing population structure and ancestry issues, could you clarify the rationale behind choosing PCoA instead of utilizing methods like Admixture or STRUCTURE, which are specifically designed to provide insights into these aspects?

Authors' response: Thank you for your insightful question regarding the choice of Principal Coordinate Analysis (PCoA) over methods like Admixture or STRUCTURE for this study. To complement our analyses, we have add the admixture results into the manuscript. Please refer to lines 276-284.

PCoA reduces a multi-dimensional dataset to a much smaller number of dimensions that allows for visual exploration and compact quantitative summaries. With admixture or STRUCTURE proportion inference, individuals in a sample are modeled as having a proportion of their genome derived from each of several source populations. The goal is to infer the proportions of ancestry from each source population, and these proportions can be used to produce compact visual summaries that reveal the existence of population structure in a sample. Another qualitative difference is that PCoA produces consistent results as more dimensions are added, whereas admixture-based methods produce qualitatively different results with different numbers of genetic components (K). Although consistency may seem a desirable property, there can be benefits to the different perspectives obtained by using different numbers of factors. In our study, the optimal number of genetic components (K) was tested using admixture based on cross-validation function (--cv), please refer to lines 198-200.

This note mentions the integration of genotypic and phenotypic data but does not provide a clear framework or methodology for how this integration was/would be achieved or its significance. Include a section that explicitly describes the integration process of genotypic and phenotypic data. Discuss the challenges faced/would be faced during integration and how they could be addressed. Highlight the importance of this integration for future breeding programs.

Authors' response: Thank you for your insightful feedback. The quality check of the integration of genotypic and phenotypic data in our study was achieved by genome-wide prediction coupled with 100 times five-fold cross-validation. Overall, the genomic-phenotypic data interoperability was in general high (please refer to lines 288-307). Additionally, genome-wide association studies were used to identify genetic markers associated with the trait, providing actionable insights for breeding decisions (please refer to reference [14]).

A key challenge in this integration process was handling large-scale datasets with missing genotypic values. To address this, we employed phasing and imputation with Beagle to ensure high-quality genotype data, followed by filtering for minor allele frequency and missing rates to enhance reliability. As for the phenotypic data, standardized field trials and precise trait phenotyping minimized environmental noise. The integration of genotypic and phenotypic data is critical for future breeding programs, offering a framework to validate markers identified through GWAS and use them in marker-assisted selection to identify potential donors for traits of interest. Moreover, by linking genetic diversity with key agronomic traits, breeders can make more informed decisions by generate more accurate predictions about trait performance, improving the efficiency of variety development and accelerating breeding cycles. Please refer to lines 79-83.

Improve the narrative flow by logically grouping related findings together.

Authors' response: Thank you for your valuable feedback. We have carefully reviewed the structure of the manuscript and have made revisions to improve the narrative flow. Specifically, we've provided clearer transitions between sections to help the reader follow the flow of the most critical findings. We hope these changes enhance the clarity and readability of the manuscript.

Emphasize the most important findings in the text to guide the reader.

Authors' response: Thank you for your valuable feedback. Key results are now highlighted in the sub-title to guide the reader. We've ensured that the central conclusions are presented in a way that underscores their significance in the context of the study's objectives.

Briefly mention any limitations observed in the results. Acknowledge any limitations of the study and their potential impact on the findings.

Authors' response: Thank you for your insightful feedback. We have acknowledged the limitations of our study in the revised manuscript. One key limitation is the relatively low

Powered by Editorial Manager® and ProduXion Manager® from Aries Systems Corporation

heritability observed for RHY in spring population and RAM in the winter population. The low disease pressure resulted in limited phenotypic variation and reduced heritability, as the environmental conditions did not sufficiently differentiate the responses of the genotypes. This diminished the ability to detect genetic contributions to the two traits.

I think authors should consider to clearly articulate the practical implications of the findings for barley breeding or agricultural practices. If their findings fail to connect to real-world applications, the relevance of the study may be questioned.

Authors’ response: Thank you for bringing this to our attention. This integrated approach also enables breeders to identify genetic markers associated with desirable traits, accelerating the development of crops with improved yield, disease resistance, or other key attributes. By combining genotypic and phenotypic data, breeders can also make more accurate predictions about trait performance, ultimately improving the efficiency of selection processes. We believe our dataset will underscore the importance of this approach in advancing breeding programs. Please refer to line 97-103.

Briefly add how environmental conditions may have influenced phenotyping results.

Authors’ response: Thank you for this valuable suggestion. We have updated the section to include a brief explanation of how non-conductive environmental conditions may have influenced the phenotyping results. Specifically, we acknowledge that variations in temperature, humidity, and precipitation across the different climate zones could impact the expression of the trait under study. For example, stress from extreme temperatures or inconsistent rainfall may alter plant growth or other phenotypic traits, potentially affecting data consistency and interpretation. Please refer to line 233-237.

Briefly add potential biological mechanisms underlying the observed traits.

Authors’ response: Thank you for your feedback and for bringing this to our attention. We appreciate the importance of discussing potential biological mechanisms for additional context. However, the primary scope of this paper is centered on the description of the dataset. A detailed examination of biological mechanisms would extend beyond the intended focus and depth of our work. Keeping the manuscript concise and aligned with our original objectives is essential to its clarity.

Integrate key points from the findings to reinforce the conclusions drawn.

Authors’ response: Thank you for your valuable suggestion. We have revised the conclusion section to better integrate key points from the findings and reinforce the conclusions drawn from our study. Please refer to line 41-43.

Reviewer2 Comments to the Author:

The manuscript by Yuan et al describes the public release of phenotypic and genotypic data of a large set of barley landraces, genebank accessions, and elite materials. The authors provide a comprehensive dataset, whole genome sequencing data together with phenotypic data for disease and agronomic traits, that will be of interest for future genetic research in barley. The document is well written, highlighting the value of the data provided. I have some comments or suggestions to further improve the document, as follows:

Authors’ response: We sincerely thank you for your thoughtful review and valuable feedback on our manuscript. We appreciate your recognition of the importance of the issues addressed in our study and your acknowledgment of the manuscript's quality.

Line 64: "enormous efforts that have been made…" Delete 'that'

Authors’ response: Thank you for your input. We have made the change accordingly in the revised version. Please refer to line 64.

L93-95: The sentence refers to genome-wide association analyses but reference [13] deals with genomic prediction. It is not a GWAS study illustrating the value of the data to select donors with novel favorable alleles. Is it the most appropriate reference?

Authors’ response: We apologize for the confusion caused. We have replaced the reference accordingly. Please refer to line 83.

L99-100: Check the following sentence, something is missing - "With the developing public access resources to enable next generations of scientists spend less time on generating and curing data, …"

Authors’ response: Thank you for pointing this out. We have revised the sentence accordingly. Please refer to line 101-103 of the revised manuscript. The revised text reads as “With the development of publicly accessible resources, the next generations of scientists can focus more on research and innovation, reducing the burden of extensive phenotyping.”

L141-142: What proportion of data were identified as outliers? Were they removed from the dataset or imputed again?

	Authors' response: We appreciate your attention to the detail. We have included the information about the proportion of outliers into Supplementary table S2. All the outliers were removed from the dataset.<br>L206: 'environment accounts' or 'the environments account'<br>Authors' response: We apologize for the confusion caused. The revised text reads as "…environment (σ_e^2) accounts for the largest proportion of the total variation…" Please refer to line 230.
Additional Information:	
Question	Response
Are you submitting this manuscript to a special series or article collection?	No
**Experimental design and statistics**<br><br>Full details of the experimental design and statistical methods used should be given in the Methods section, as detailed in our Minimum Standards Reporting Checklist. Information essential to interpreting the data presented should be made available in the figure legends.<br><br>Have you included all the information requested in your manuscript?	Yes
**Resources**<br><br>A description of all resources used, including antibodies, cell lines, animals and software tools, with enough information to allow them to be uniquely identified, should be included in the Methods section. Authors are strongly encouraged to cite Research Resource Identifiers (RRIDs) for antibodies, model organisms and tools, where possible.<br><br>Have you included the information requested as detailed in our Minimum Standards Reporting Checklist?	Yes
**Availability of data and materials**<br><br>All datasets and code on which the conclusions of the paper rely must be either included in your submission or deposited in publicly available repositories	Yes

Powered by Editorial Manager® and ProduXion Manager® from Aries Systems Corporation

(where available and ethically appropriate), referencing such data using a unique identifier in the references and in the “Availability of Data and Materials” section of your manuscript.<br><br>Have you have met the above requirement as detailed in our Minimum Standards Reporting Checklist?	

Manuscript with DOI

Click here to access/download;Manuscript;manuscript-Yuan.et.al-20241203_rev1 with DOI.docx

High-quality phenotypic and genotypic dataset of barley genebank core-collection to unlock untapped genetic diversity

Zhihui Yuan[1], Maximilian Rembe[1,2], Martin Mascher[1,3], Nils Stein[1,4], Axel Himmelbach[1], Murukarthick Jayakodi[1], Andreas Börner[1], Klaus Oldach[5], Ahmed Jahoor[6], Jens Due Jensen[6], Julia Rudloff[7], Viktoria-Elisabeth Dohrendorf[8], Luisa Pauline Kuhfus[9], Emmanuelle Dyrszka[9], Matthieu Conte[9], Frederik Hinz[10], Salim Trouchaud[11], Jochen C. Reif[1], Samira El Hanafi[1]

[1]Leibniz Institute of Plant Genetics and Crop Plant Research (IPK) Gatersleben, Seeland, Germany

[2]KWS SAAT SE & Co. KGaA, Grimsehlstr. 31, 37574 Einbeck, Germany

[3]German Centre for Integrative Biodiversity Research (iDiv) Halle-Jena-Leipzig, Leipzig, Germany

[4]Crop Plant Genetics, Institute of Agricultural and Nutritional Sciences, Martin-Luther-University of Halle-Wittenberg, Halle (Saale), Germany

[5]KWS LOCHOW GmbH, Ferdinand-von-Lochow-Str. 5, 29303 Bergen, Germany

[6]Nordic Seed Germany GmbH, Kirchhorster Str. 16 31688 Nienstädt, Germany

[7]Limagrain GmbH, Salderstr. 4, 31226 Peine-Rosenthal, Germany

[8]Nordsaat Saatzucht GmbH, Zuchtstation Gudow, Hofweg 8, D-23899 Gudow, Germany

[9]Syngenta France SAS, 12 Chemin de l'hobit, B.P. 27, 31790, Saint-Sauveur, France

[10]Saatzucht Bauer GmbH & CO.KG, Landshuter Straße 3a, 93083 Obertraubling, Germany

[11]Secobra Saatzucht GmbH, Feldkirchen 3, 85368 Moosburg an der Isar, Germany

corresponding author: Samira El Hanafi (hanafi@ipk-gatersleben.de)

Abstract

Background: Genebanks around the globe serve as valuable repositories of genetic diversity, offering not only access to a broad spectrum of plant material but also critical resources for enhancing crop resilience, advancing scientific research, and supporting global food security. To this end, traditional genebanks are evolving into bio-digital resource centres where the integration of phenotypic and genotypic data for accessions can drive more informed decision-

making, optimize resource allocation, and unlock new opportunities for plant breeding and research. However, the curation and availability of interoperable phenotypic and genotypic data for genebank accessions is still in its infancy and represents an obstacle to rapid scientific discoveries in this field. Therefore, effectively promoting FAIR, i.e. findable, accessible, interoperable, and reusable, access to these data is vital for maximizing the potential of genebanks and driving progress in agricultural innovation.

Findings: Here we provide whole genome sequencing data of 812 barley (*Hordeum vulgare* L.) plant genetic resources (PGRs) and 298 European elite materials released between 1949 and 2021, as well as the phenotypic data for four disease resistance traits and three agronomic traits. The robustness of the investigated traits and the interoperability of genomic and phenotypic data were assessed in the current publication, aiming to make this panel publicly available as a resource for future genetic research in barley.

Conclusions: The data showed broad phenotypic variability and high association mapping potential, offering a key resource for identifying genebank donors with untapped genes to advance barley breeding while safeguarding genetic diversity.

Keywords

Barley; plant genetic resources; elite; whole genome resequencing; disease resistance; agronomic traits

Data Description

Context

Successful plant breeding programs rely on balanced efforts between short-term goals to develop competitive cultivars and the maintenance of a broad genetic pool to guarantee long-term progress. In practice, the development of new varieties has been predominantly derived by recycling existing elite lines, leading to important genetic improvement and the reduction in the genetic diversity of elite germplasm. This could impede the breeding of potential new varieties capable of addressing and responding to constraints related to climate change, agronomical threads, and meeting the escalating social demands [1]. To overcome these limitations,

leveraging genetic diversity harbored within plant genetic resources (PGR) has been frequently suggested [2]. PGRs provide a valuable reservoir of untapped genetic potential that can be utilized to develop varieties with improved yield [3] and end-use quality, and enhanced resistance to both biotic and abiotic stresses, such as diseases [4], pests [5], waterlogging [6], salinity [7], and drought [6,8].

As the most cost-effective *ex situ* conservation strategy, genebanks worldwide are committed to maintaining PGRs, which hold a diverse gene pool encompassing all the alleles of various genes, including those from wild species, landraces, and breeding stocks. However, although enormous efforts have been made to conserve germplasm [9], it is estimated that less than 1% of the resources preserved in genebanks have been used in crop improvement [10]. The great challenge for breeders and scientists lies in finding useful barley PGRs among entire genebank collections that are comprised of thousands of accessions with complex patterns of genetic diversity [11]. Therefore, core collections were proposed as a strategy to streamline operational processes and mitigate costs, thereby facilitating more precise and effective research and breeding initiatives. Over the past decades, this approach has become even more attractive thanks to recent technological advancements, which have markedly reduced the costs of genotyping, and led to dramatic improvements in read length, sequencing chemistry, instrumentation, and throughput [12]. As a result, generating large-scale sequencing and genotyping datasets for entire genebank collections is now feasible. This has greatly expanded the scope of genotyping efforts and underpinned the effective selection of core collections that maximize genetic diversity [13]. These advancements provide powerful tools to efficiently harness PGRs, enabling the identification of valuable and favorable genes. This has streamlined their incorporation into crop improvement efforts, ultimately speeding up the development of new and improved varieties. Coupled with extensive and high-quality phenotypic data, the systematic use of whole genome sequencing data could provide valuable insights into genetic diversity and potential breeding opportunities. Our recent findings using genome-wide association analyses highlighted the value of these data in selecting donors with potentially novel favorable genes [14].

Moreover, the strategic deployment of core collections becomes even more compelling when combined with modern elite material [15,16], which serves as a reference panel to define favorable alleles/genes that are absent in the elite panel. This integrated approach is essential for enhancing polygenic traits and, hence, achieving informed pre-breeding decisions. To put this into practice, we selected a barley core collection [17] from the German Federal *ex situ* Genebank for Agriculture and Horticultural Crops at the Leibniz Institute of Plant Genetics and Crop Plant Research (IPK) and combined it with a set of European elite material. This population was designed to: i) phenotype the whole population in multi-environmental trials for three agronomical traits: plant height (PLH), heading date (HD), lodging (LOD), and four disease traits: *Puccinia hordei* (PUC), *Blumeria graminis hordei* (BLU), *Ramularia collo-cygni* (RAM), and *Rhynchosporium commune* (RHY); ii) evaluate the interoperability quality for the phenotypic and genomic datasets using five-fold cross-validation; iii) conduct the mantel test to check the detection power in association mapping analyses.

The data presented here can be further extended with additional PGRs and/or elite materials. It can also be integrated with alternative strategies to improve the utilization of germplasm collections by selecting untapped PGR donors, such as the development of novel association mapping methods. This will enable breeders to make more accurate predictions of trait performance, thereby enhancing the efficiency of selection processes. Furthermore, with the development of publicly accessible resources, scientists will be able to focus more on research and innovation, while reducing the burden of extensive phenotyping. The insights derived from our data may significantly accelerate advancements in genomic research and breeding programs, driving improvement and fostering future collaboration and resource sharing.

Methods

Barley material and field trials

To capture a broad spectrum of geographic origins and wide genetic diversity, we selected 812 PGRs which include 288 spring type (PGR_Spring) and 524 winter type (PGR_Winter), originating from 57 countries spanning 5 continents. Based on their performance during seed regeneration, these PGRs were thoughtfully selected from a previously described barley core

1000 collection [17], as a representative subset of the entire 21,405 barley accessions available at the IPK genebank [18], based on their performance during seed regeneration. Additionally, we incorporated 298 elite lines, including ten local checks, which consist of 128 spring type (Elite_Spring) and 170 winter type (Elite_Winter). These elites were exclusively selected from the European registered varieties and were available through the seed market, showcasing the breeding process over time from 1949 to 2021. The study initially included 87 additional genotypes which were later excluded from certain analyses due to incomplete phenotypic or genotypic data. To maintain the integrity of the dataset and facilitate accurate adjustments for experimental design effects, we retained all relevant data, including instances of missing information.

Field trials were conducted over three consecutive years (2020, 2021, and 2022) across eight locations in Germany: KWS-L/Prosselsheim (49°51'15.6"N, 10°06'04.1"E; 10.9ºC average annual temperature; 565.3 mm average annual rainfall); Nordic Seed/Nienstädt (52°17'35.52"N, 9°08'57.156"E; 10.7ºC average annual temperature; 638.4 mm average annual rainfall); Saatzucht Bauer/Riekofen (48°54'55.98"N, 12°21'21.744"E; 9.9ºC average annual temperature; 690.3 mm average annual rainfall); Limagrain/Peine-Rosenthal (52°18'09.828"N, 10°10'28.488"E; 10.9ºC average annual temperature; 607.5 mm average annual rainfall); Nordsaat/Gudow (53°33'28.0"N, 10°47'50.5"E; 10.4ºC average annual temperature; 581.1 mm average annual rainfall); Syngenta/Bad Salzuflen (52°04'21.576"N, 8°41'55.86"E; 10.5ºC average annual temperature; 692.8 mm average annual rainfall); Secobra-LEM/Lemgo (52°00'41.6"N, 8°52'22.7"E; 10.7ºC average annual temperature; 714.3 mm average annual rainfall); Secobra-FK/Moosburg (48°28'46.8"N, 11°54'32.6"E; 10.7ºC average annual temperature; 743.1 mm average annual rainfall). The trials were sown following a generalized alpha lattice design, which organizes genotypes into incomplete blocks to minimize spatial variation. Two-row observation plots (1 m^2) with two replications were used, and ten checks were included across years and locations for consistency. Each unique combination of year and location was considered as a distinct environment.

Phenotyping

The whole population was phenotyped for three agronomy traits for their importance in barley adaptability, yield potential, and harvestability: heading date measured in days from January 1st for winter type and from the sowing date onward for the spring type; plant height measured from the soil surface to the tip of spike in cm (excluding awns); and lodging rated on a 1-9 scale (with a higher score indicating severe lodging). Additionally, four disease traits including *Puccinia hordei*, *Blumeria graminis hordei*, *Ramularia collo-cygni*, and *Rhychnosporium commune* were evaluated under natural infection conditions. The disease severities were scored using an ordinal scale from 1 (fully resistant) to 9 (fully susceptible) following the guidelines of the German Federal Plant Variety Office [19].

Phenotypic data analyses

A linear mixed model using restricted maximum likelihood (REML) method [20] was used for data analyses across environments for spring and winter barley separately. Phenotypic data was corrected for outliers following the method of Tukey and Anscombe [21]. The residuals were extracted then normalized to flag the outliers according to a predefined significance threshold of p-value < 0.01 (Supplementary Table S2). Variance components and best linear unbiased estimations (BLUEs) of each genotype were computed from the oulier-corrected data following model (1):

$$y_{ijkm} = \mu + E_m + g_i + g_i \times E_m + E_m{:}r_j : b_k + e_{ijkm}, \qquad (1)$$

where y_{ijkm} denoted the vector of phenotypic values for i^{th} genotype (g) tested in k^{th} bock (b) nested in j^{th} replication (r) in mth environment (E), μ was the common mean, and e denoted the error term of the model. We assumed that all random effects followed an independent normal distribution with different variance components. In the model (1), all terms except μ and g_i were considered random for deriving the BLUEs across environments, whereas all terms except μ were modelled as random to estimate variance component for deriving heritability following model (2):

$$H^2 = \frac{\sigma_g^2}{\sigma_g^2 + \frac{\sigma_{g\times E}^2}{n_E} + \frac{\sigma_e^2}{n_R}}, \qquad (2)$$

where σ_g^2 denoted the genotypic variance, $\sigma_{g\times E}^2$ denoted the interaction between genotype and environment, σ_e^2 denoted the residual variance, and $\overline{n_R}$ denoted the average number of replications per genotype, $\overline{n_E}$ denotes the average number of environments in which the genotypes were evaluated. ASReml-R [22] was employed for all mixed linear models that were applied in the phenotypic analysis.

Whole genome shotgun sequencing

Whole genome sequencing (WGS) of the 1,110 genotypes (812 PGRs and 298 elite lines) was performed at IPK Gatersleben. High molecular weight (HMW) DNA was extracted from the leaves (8g) of greenhouse-grown (21°C/18°C day/night temperature) 7-day-old seedlings following a previously established protocol [23]. The Illumina Nextera libraries were prepared and sequenced using the Illumina NovaSeq 6000 platform [24]. Raw sequencing reads were trimmed using cutadapt [version 3.3; 25] and aligned to MorexV3 reference genome [26] using Minimap2 [version 2.20; 27]. The resultant alignment records were sorted with Novosort (V3.09.01; http://www.novocraft.com). Finally, a total of 149,380,812 single-nucleotide polymorphisms (SNPs) for the 1,110 genotypes were initially outputted by BCFtools [version 1.9; 28].

Quality control for SNP data

The resulting raw genotypic data was used to extract the corresponding datasets of the four sub-groups. Only bi-allelic SNPs with a minor allele frequency > 0.05 and missing rate < 0.1 were retained by PLINK [version 1.9; 29] for each of the four sub-groups. These meticulous steps yielded datasets comprising 17,759,260 SNPs for Elite_Spring, 26,903,811 for Elite_Winter, 54,934,336 for PGR_Spring, and 46,434,685 for PGR_Winter.

The resulting filtered genotypic data were used as input to phase and impute missing values using Beagle [version 5.2; 30], leveraging linkage disequilibrium to infer missing data accurately. Subsequently, an r^2 cutoff of 0.2 was set to prune markers by PLINK (version 1.9) with a sliding window size of 50 kb, and a step size of 10 kb. The final number of SNPs available differed in the four sub-groups due to the aforementioned process: 710,855 of Elite_Spring, 945,074 of Elite_Winter, 2,321,327 of PGR_Spring, and 1,775,972 of PGR_Winter. For each

tested SNP, homozygous for the most frequent allele, heterozygous, and homozygous for the alternative allele were coded as 0, 1, and 2 by PLINK (version 1.9), respectively.

Population structure

Subsequently, the aforementioned post-quality-control markers were used to investigate the population structure within and across spring and winter barley accessions using principal coordinate analysis (PCoA) based on pairwise Rogers' distance [31]. PCoA was performed using the R package ape [version v5.7-1; 32]. Additionally, the population structure was tested using ADMIXTURE [version 1.3.0; 33]. The optimal number of population components was determined based on cross-validation function (--cv).

Moreover, linkage disequilibrium (LD) analyses of the four sub-groups was carried out separately by determining the pairwise squared allele-frequency correlations (r^2) between markers [34] and then combined to estimate LD decay across the entire genome. A decay curve was fitted for each sub-group using nonlinear regression of pairwise r^2 against the distance (Mb) between the markers. LD within a specific physical distance of 2 Mb was calculated and visualized using PopLDdecay [version 3.40; 34].

Genomic-phenotypic data interoperability

To evaluate the interoperability for the phenotypic and genomic datasets, we calculated the accuracy of the genomic best linear unbiased prediction (GBLUP) [35]. First, the mixed model equations for genomic prediction were computed using REML in the rrBLUP R package [v4.6.1; 36]. Prediction accuracies were then estimated through five-fold cross validation. In this process, both phenotypic and genomic datasets were randomly subdivided into five groups. The first four groups served together as the training set, whereas the fifth group corresponded to the prediction set. The random sampling was repeated 100 times, giving a total of 500 cross-validation runs. Genomic prediction ability was thereafter defined as the correlation between BLUEs across environments for a trait and the corresponding predicted values.

Mantel correlation

Following the imputation process, we used PLINK (version 1.9) to construct a genetic relationship matrix. To further explore the association between phenotypic variation and

population structure, the correlation between the genetic relationship matrix and the absolute trait differences (Euclidean distance matrix) in each sub-group was tested using a Mantel test [38] implemented in the R package vegan [v2.6-4; 36] , and visualized by linkET R package [v0.0.7.4; 37], 999 permutations were used to evaluate the significance of the test.

Data Validation and quality control

High heritability estimation highlights the robustness of the phenotypic data

The quality and reliability of the phenotypic data were rigorously assessed by estimating the heritability of the evaluated traits. After outlier correction, the heritability estimates for most traits were generally high, exceeding 0.5 (Fig. 1A). Notable exceptions included RHY in the spring population (h^2 = 0.05), and RAM (h^2 = 2E-06) in the winter population. Variance components analysis revealed that environment (σ_e^2) accounts for the largest proportion of the total variance, while genotype and genotype × environments interaction were less pronounced, with the exception of LOD and RHY in both spring and winter population, as well as PUC in winter population (Fig. 1B). This suggests that factors such as temperature fluctuations, varying levels of precipitation, and humidity across different climate zones may have influenced the observed phenotypic performance. These environmental conditions likely influenced growth patterns and trait expression, leading to larger phenotypic variability in traits with high heritability and restricted variability in traits with low heritability.

The resulting BLUEs showed normal distribution for most disease traits (Fig. 2). However, RHY showed left skew in both spring and winter population, while BLU and PUC displayed left skew in elite population for both spring and winter type. The left skew of RHY suggests low disease pressure across three years and, hence, resulting in a small proportion of susceptible genotypes. And the left skew of elite population of BLU and PUC suggests that PGRs tend to be more susceptible than the elite materials for the two diseases. For agronomic traits (Fig. 3), PGR population showed normal distribution, while elite lines showed normal distribution in HD and PLH only in winter population.

Furthermore, several significant correlations were observed between the evaluated traits (Fig. 4). For pairing of agronomic and disease traits, it was observed that HD was negatively

correlated with all the disease traits, except for BLU in winter barley population. Those observations suggest a strategic plant response given that delayed heading allows plants to evade disease infection through spatial or temporal adjustments. Moreover, LOD was positively correlated with all the disease traits, except for RAM in spring barley population. PLH was positively correlated with BLU and PUC while negatively correlated with RAM and RHY.

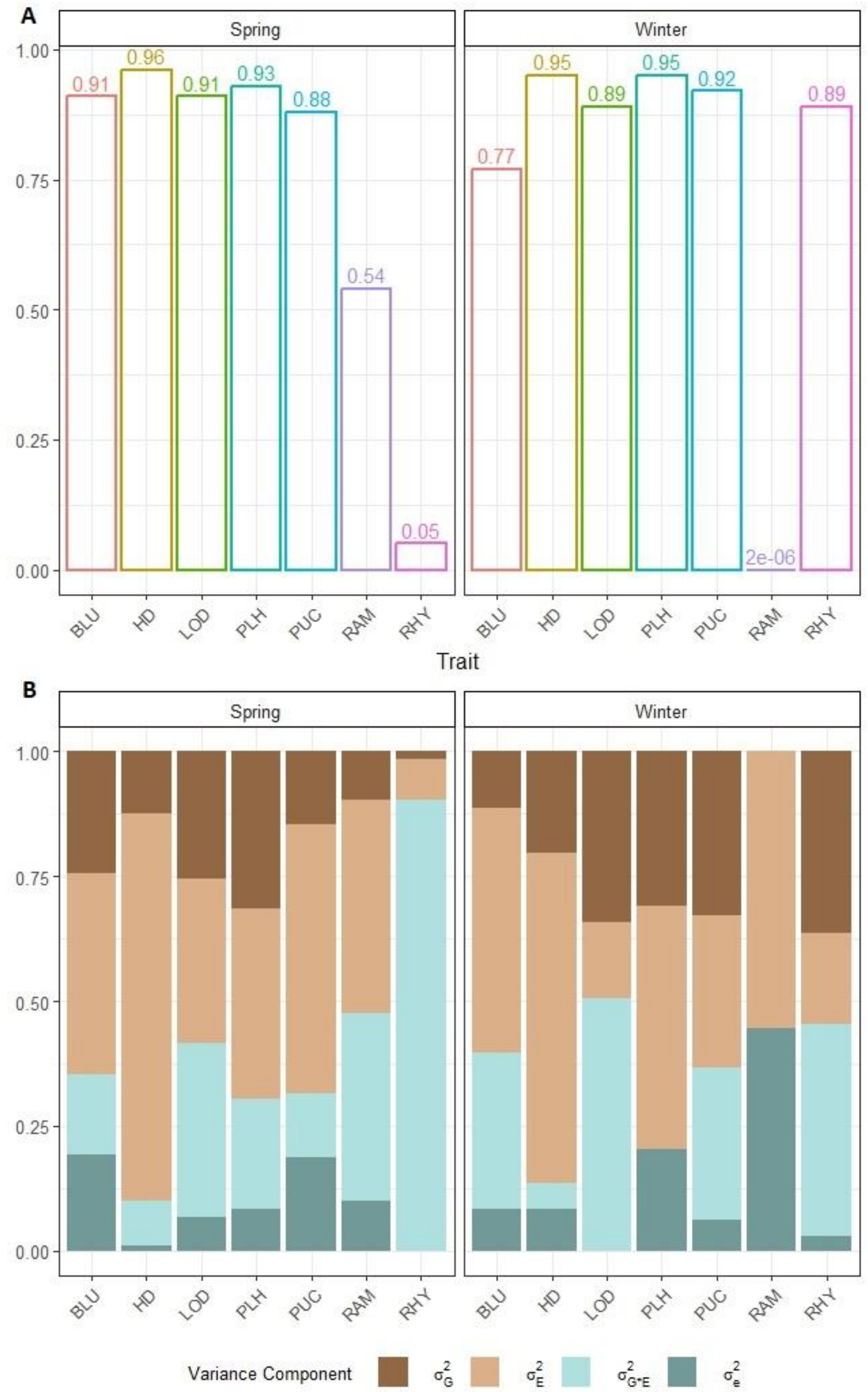

Figure 1: Heritability (A) and percentages of the different variance components (B) for the seven traits considered in this study. BLU: *Blumeria graminis hordei*; PUC: *Puccinia hordei*; RHY: *Rhynchosporium commune*; RAM: *Ramularia collo-cygni*; HD: heading date; PLH: plant height; LOD: lodging; σ^2_G: genotypic variance; σ^2_{G*E}: variance due to genotype by environment interaction; σ^2_E: variance due to environment; σ^2_e: residual.

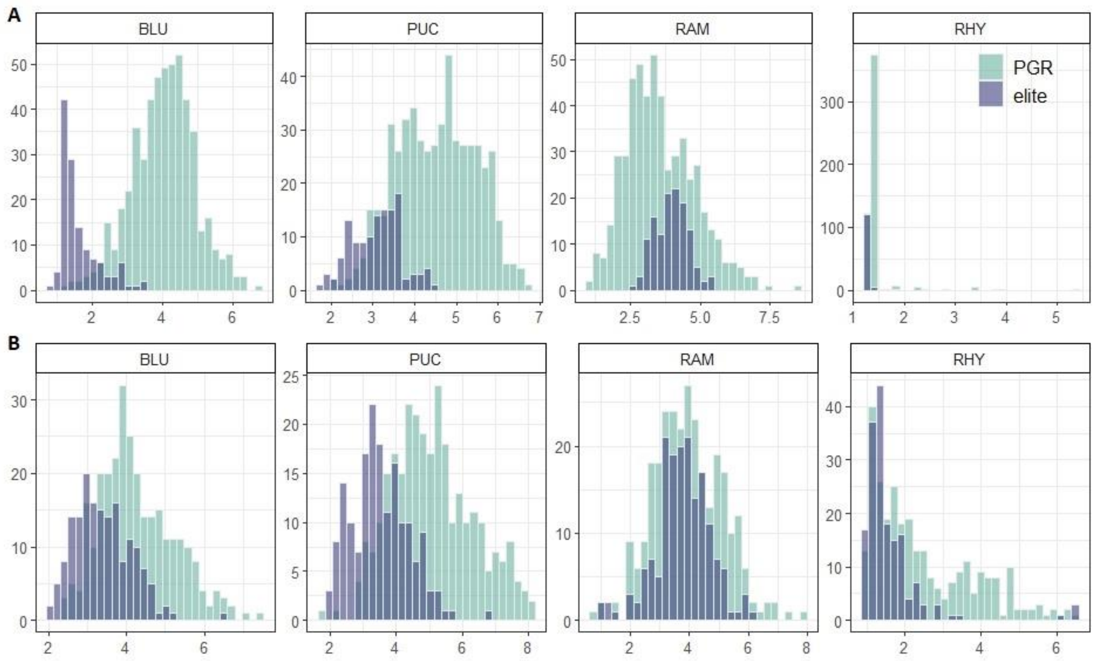

Figure 2: Histogram showing the phenotypic distribution for four diseases traits for spring (A) and winter (B) population. BLU: *Blumeria graminis hordei*; PUC: *Puccinia hordei*; RHY: *Rhynchosporium commune*; RAM: *Ramularia collo-cygni*.

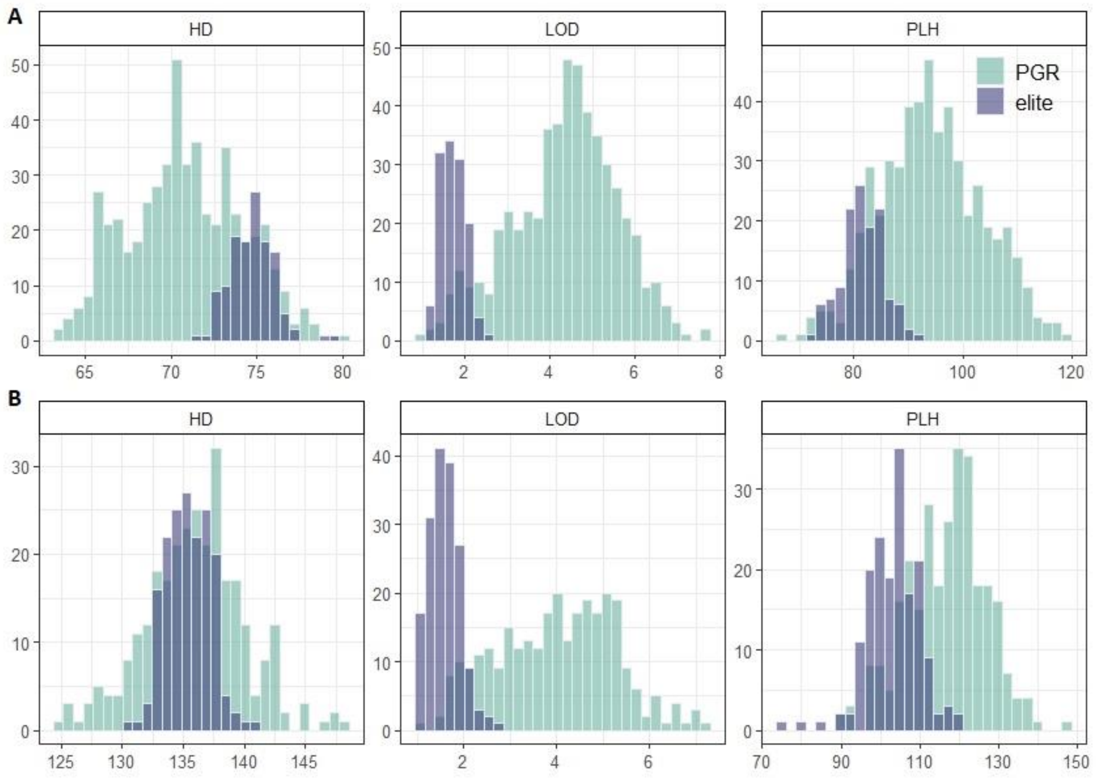

Figure 3: Histogram showing the phenotypic distribution for three agronomic traits for spring (A) and winter (B) population. HD: heading date; PLH: plant height; LOD: lodging.

Whole genome sequencing data showed high genetic diversity and high marker densities

Whole genome sequencing (WGS) data of the 1,110 genotypes showed an average coverage of 4.7x with a range spanning from 0.5x to 22.6x across all samples with mapping rate from 94% to 99%, providing a solid foundation for downstream genetic analyses and ensuring a comprehensive representation of the genomic information across the diverse set of genotypes.

Building on this comprehensive genomic dataset, we performed PCoA to assess the genetic diversity among the spring and winter barley population as reported in our companion study [14]. The first two coordinates explained together 11.66% and 11.25% of the spring and winter population, respectively. As anticipated, the inclusion of PGRs significantly broadened the genetic diversity compared to the elite materials. Notably, the elite spring population formed a tight, cohesive cluster indicating less genetic diversity, while the elite winter population exhibited a more dispersed pattern reflecting greater genetic variability.

To further complement the population structure analyses, the optimal number of genetic components (K = 3) was determined based on cross-validation results. The admixture analysis revealed distinct population structures within the spring and winter populations (Fig. 4), with individuals showing varying proportions of the three inferred components. These results highlight the contrasting levels of genetic diversity and population structure within each spring and winter barley genotypes.

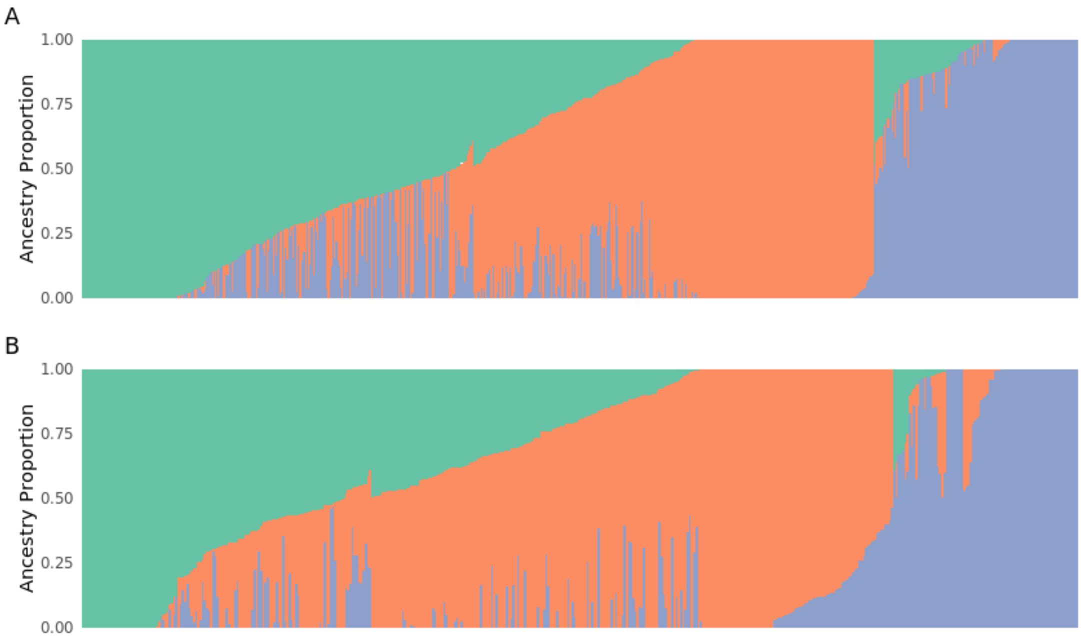

Figure 4: Admixture analysis of spring (A) and winter (B) populations with the K = 3 admixture model. Each individual is represented as a vertical bar with colour corresponding to the proportions of three ancestral components (K).

For the intra-chromosomal decay of LD (r^2), PGR was faster in both spring and winter population as compared to elite materials. The slower LD decay in elite population may be due to genetic bottlenecks and/or high selection pressures that produce specific linkage between alleles that control specific phenotypes.

High genomic prediction accuracies support the interoperability of genomic and phenotypic data

Systematic errors can occur during field trials, which will systematically disrupt the connectivity between genotype and phenotype data and, in turn, decrease the value of the data for subsequent integrated analyses. To assess potential data imbalances, we used the cross-validated accuracy of genomic prediction as a quality measure for genomic-phenotypic data interoperability.

Integrating phenotypic data with WGS data resulted in 652 spring and 458 winter barley genotypes. Overall, the genomic-phenotypic data interoperability was in general high (Fig. 5), with a maximum prediction accuracy observed for lodging in both spring and winter populations. Disease-resistant traits showed moderate to high prediction abilities, suggesting

that genomic data can be reliably used, thereby potentially accelerate breeding efforts for resistant varieties. In parallel, this robust result ensures reliable data quality, enabling comprehensive analyses to explore genotype-phenotype relationships, and lay a solid foundation for future studies aimed at finding marker-trait associations and understanding the genetic mechanisms underlying key traits in barley.

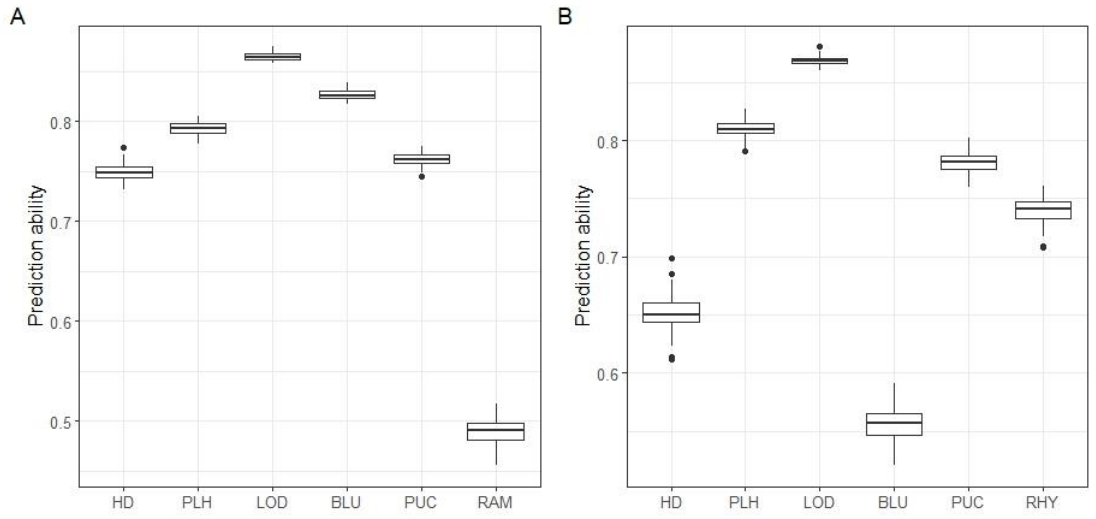

Figure 5: Five-fold cross-validation abilities of the genomic best linear unbiased prediction for heading date (HD; days); plant height (PLH; cm); lodging (LOD); *Blumeria graminis hordei* (BLU); *Puccinia hordei* (PUC); *Rhynchosporium commune* (RHY); *Ramularia collo-cygni* (RAM), obtained in the spring (A) and winter (B) populations.

Mantel test results indicate a high detection power in association mapping

Accurate mapping requires addressing the complexities inherent in genetic relatedness among individuals. In such way, especially when dealing with panels comprising both elite lines and PGRs, the intricate patterns of genetic relationship can pose significant challenges. Specially, when phenotype variation is influenced by genetic relatedness, it becomes crucial to differentiate between genuine associations and those resulting from shared genetic backgrounds. This complexity underscores the importance of robust methods, to effectively uncover meaningful correlations and enhance the reliability of association mapping. Therefore, by minimizing genotype-phenotype covariance, we can reduce the risk of spurious associations [41]. Mantel test is a widely used approach to examine the association between two matrices. The results revealed a moderate to low correlation between genetic distance and Euclidean

phenotypic distance matrix, indicating a lack of strong association between phenotypic variation and genome-wide genetic differences (Fig. 6; Mantel's r in spring barley ranged from -0.02 to 0.29, and from 0 to 0.32 in winter barley), which in turn expected to increase the detection power in association mapping.

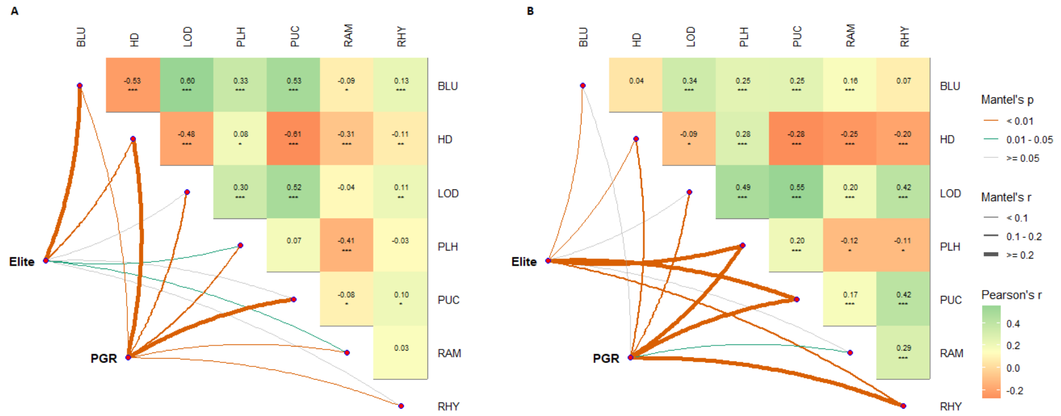

Figure 6: Pairwise correlations for the recorded traits, and the Mantel tests between tested traits vs. elite materials and plant genetic resources (PGR) for spring barley (A) and winter barley (B). The lines represent significant relationships, where the width of the line represents the Mantel r statistic value and the different colours of the lines represent different degrees of significance. The Pearson correlation coefficient between different traits is shown in the heat map matrix. BLU: *Blumeria graminis hordei*; PUC: *Puccinia hordei*; RHY: *Rhynchosporium commune*; RAM: *Ramularia collo-cygni*; HD: heading date; PLH: plant height; LOD: lodging; *** p < 0.001, ** p < 0.01, * p < 0.05.

Data Availability

Phenotypic records: The raw phenotypic data described here as well as the ready-to-use phenotypic values (BLUEs), and the R script to import and curate the raw phenotypic data to compute heritability and BLUEs are available in the e!DAL-PGP Repository [42] and can be directly accessed here [43].

Raw sequencing reads: FASTQ files containing raw reads for 1,110 genotypes were submitted by [24], and deposited at the European Nucleotide Archive [44] under BioProjects PRJEB53924 (Illumina resequencing data). Sequenced genotypes are findable through their 'SAMEA' IDs. The integrated Elite and PGR 'SAMEA' BioSample IDs connected with plant material

passports, passport data sources, SSD- and IPK genebank DOIs were listed in Supplementary Table S1.

SNP markers: variant calling results based on read mapping against the reference sequence of MorexV3 were stored as Variant Call Format (VCF). All the VCF files are located at the European Nucleotide Archive under the project number PRJEB80159.

The script for filtering VCF files, imputation, admixture process, mantel test, and cross validation is accessible at https://github.com/yzh1023/data-publication.git [45]. Other data further supporting this work are openly available in the *GigaScience* repository, GigaDB [46].

Additional files

Supplementary Table S1. List of 1,110 genotypes in this dataset.

Supplementary Table S2. The number and proportion of outliers identified for each trait.

Abbreviations

BLU: *Blumeria graminis hordei*; BLUE: best linear unbiased estimations; HD: heading date; IPK: Institute of Plant Genetics and Crop Plant Research; LD: linkage disequilibrium; LOD: lodging; PCoA: principal coordinate analysis; PGR: plant genetic resources; PLH: plant height; PUC: *Puccinia hordei*; RAM: *Ramularia collo-cygni*; RHY: *Rhynchosporium commune*; SNP: single-nucleotide polymorphisms; WGS: whole genome sequencing.

Author's Contributions

KO, AJ, JDJ, JR, VD, LPK, ED, MC, FH, and ST: cultivation and provision of phenotypic data of all spring and winter barleys over 3 years in one to two locations; ZY: genotypic data analyses and curation; MR and SEH: phenotypic data analyses; MM, MJ, AH, and NS: generated and processed the genomic data; NS, MM, AB, SEH, and JCR: edited and revised the manuscript; AB: developed the core 1000 population; JCR, NS, SEH, and ZY: designed the study; ZY and SEH: wrote the paper. All authors read and approved the final manuscript.

Funding

This research work is funded by German Ministry of Food and Agriculture under the project Structural genome variation, haplotype diversity and the barley pan-genome - Exploring structural genome diversity for barley breeding (SHAPE) phase 1 and 2 (BMBF FKZ 031B0190A; 031B0884A).

Competing Interests

The authors declare that they have no competing interests.

Acknowledgement

We are grateful for the technical assistance of Mary Ziems and Annette Marlow for providing seeds of plant material. Susanne König, and Ines Walde for technical assistance during sequencing data production, as well as Anne Fiebig, Daniel Arend and Matthias Lange for support with data management and submission to repositories.

References

1. Ellegren H, Galtier N. Determinants of genetic diversity. *Nat Rev Genet.* 2016;17:422–433.

2. Halewood M, Chiurugwi T, Sackville Hamilton R *et al.* Plant genetic resources for food and agriculture: opportunities and challenges emerging from the science and information technology revolution. *New Phytologist* 2018;217:1407–1419.

3. Dillon SL, Shapter FM, Henry RJ *et al.* Domestication to Crop Improvement: Genetic Resources for *Sorghum* and *Saccharum* (Andropogoneae). *Annals of Botany* 2007;100:975–989.

4. Deng Y, Ning Y, Yang D *et al.* Molecular Basis of Disease Resistance and Perspectives on Breeding Strategies for Resistance Improvement in Crops. *Molecular Plant* 2020;13:1402–1419.

5. Radchenko EE, Abdullaev RA, Anisimova IN. Genetic Resources of Cereal Crops for Aphid Resistance. *Plants* 2022;11:1490.

6. Valliyodan B, Ye H, Song L *et al.* Genetic diversity and genomic strategies for improving drought and waterlogging tolerance in soybeans. *J Exp Bot.* 2016;68:1835–1849.

7. Razzaq A, Saleem F, Wani SH *et al.* De-novo Domestication for Improving Salt Tolerance in Crops. *Front Plant Sci.* 2021;12:681367.

8. Missanga JS, Venkataramana PB, Ndakidemi PA. Recent developments in *Lablab purpureus* genomics: A focus on drought stress tolerance and use of genomic resources to develop stress-resilient varieties. *Legume Science* 2021;3:e99.

9. Wambugu PW, Ndjiondjop M-N, Henry RJ. Role of genomics in promoting the utilization of plant genetic resources in genebanks. *Briefings in Functional Genomics* 2018;17:198–206.

10. Sharma S, Upadhyaya HD, Varshney RK *et al.* Pre-breeding for diversification of primary gene pool and genetic enhancement of grain legumes. *Front Plant Sci* 2013;4:309.

11. Odong TL, Jansen J, van Eeuwijk FA *et al.* Quality of core collections for effective utilisation of genetic resources review, discussion and interpretation. *Theor Appl Genet.* 2013;126:289–305.

12. Salgotra RK, Chauhan BS. Genetic Diversity, Conservation, and Utilization of Plant Genetic Resources. *Genes* 2023;14:174.

13. El Hanafi S, Jiang Y, Kehel Z *et al.* Genomic predictions to leverage phenotypic data across genebanks. *Front Plant Sci.* 2023;14:1227656.

14. Yuan ZH, Rembe M, Mascher M *et al.* Capitalizing genebank core collections for rare and novel disease resistance loci to enhance barley resilience. *Journal of Experimental Botany* 2024:erae283.

15. Cazenave X, Petit B, Lateur M *et al.* Combining genetic resources and elite material populations to improve the accuracy of genomic prediction in apple. *G3 Genes|Genomes|Genetics* 2022;12:jkab420.

16. Sehgal D, Vikram P, Sansaloni CP *et al.* Exploring and Mobilizing the Gene Bank Biodiversity for Wheat Improvement. *PLoS ONE* 2015;10:e0132112.

17. Milner SG, Jost M, Taketa S *et al.* Genebank genomics highlights the diversity of a global barley collection. *Nat Genet.* 2019;51:319–326.

18. Oppermann M, Weise S, Dittmann C *et al.* GBIS: the information system of the German Genebank. *Database* 2015;2015:bav021.

19. Bundessortenamt. Richtlinien für die Durchführung von landwirtschaftlichen Wertprüfungen und Sortenversuchen. 2000.

20. Patterson HD, Thompson R. Recovery of inter-block information when block sizes are unequal. *Biometrika* 1971;58:545–554.

21. Anscombe FJ, Tukey JW. The Examination and Analysis of Residuals. *Technometrics* 1963;5:141–160.

22. Butler DG, Cullis BR, Gilmour AR *et al.* ASReml estimates variance components under a general linear. 2023.

23. Dvorak J, McGuire PE, Cassidy B. Apparent sources of the A genomes of wheats inferred from polymorphism in abundance and restriction fragment length of repeated nucleotide sequences. *Genome* 1988;30:680–689.

24. Jayakodi M, Lu Q, Pidon H *et al.* Structural variation in the pangenome of wild and domesticated barley. *Nature* 2024. https://doi.org/10.1038/s41586-024-08187-1.

25. Martin M. Cutadapt removes adapter sequences from high-throughput sequencing reads. *EMBnet.journal* 2011;17:10.

26. Mascher M, Wicker T, Jenkins J *et al.* Long-read sequence assembly: a technical evaluation in barley. *The Plant Cell* 2021;33:1888–1906.

27. Li H. Minimap2: pairwise alignment for nucleotide sequences. *Bioinformatics* 2018;34:3094–3100.

28. Li H. A statistical framework for SNP calling, mutation discovery, association mapping and population genetical parameter estimation from sequencing data. *Bioinformatics* 2011;27:2987–2993.

29. Purcell S, Neale B, Todd-Brown K *et al.* PLINK: A Tool Set for Whole-Genome Association and Population-Based Linkage Analyses. *The American Journal of Human Genetics* 2007;81:559–575.

30. Browning BL, Zhou Y, Browning SR. A One-Penny Imputed Genome from Next-Generation Reference Panels. *The American Journal of Human Genetics* 2018;103:338–348.

31. Rogers JS. Measures of genetic similarity and genetic distance. *Studies in genetics VII* 1972:145–153.

32. Paradis E, Schliep K. ape 5.0: an environment for modern phylogenetics and evolutionary analyses in R. Schwartz R (ed.). *Bioinformatics* 2019;35:526–528.

33. Alexander DH, Novembre J, Lange K. Fast model-based estimation of ancestry in unrelated individuals. *Genome Res.* 2009;19:1655–1664.

34. Hill WG, Robertson A. Linkage Disequilibrium in Finite Populations. *Theoretical and Applied Genetics* 1968;38:226–231.

35. Zhang C, Dong SS, Xu JY *et al.* PopLDdecay: A fast and effective tool for linkage disequilibrium decay analysis based on variant call format files. *Bioinformatics* 2019;35:1786–1788.

36. VanRaden PM. Efficient Methods to Compute Genomic Predictions. *Journal of Dairy Science* 2008;91:4414–4423.

37. Endelman JB. Ridge Regression and Other Kernels for Genomic Selection with R Package rrBLUP. *The Plant Genome* 2011;4:250–255.

38. Mantel N. The detection of disease clustering and a generalized regression approach. *Cancer Res.* 1967;27:209–220.

39. Oksanen J, Simpson GL, Blanchet FG *et al.* vegan: Community Ecology Package. 2022.

40. Huang H. LinkET: everything is linkable. *R package version 0.0.7.4.* 2021;3.

41. Myles S, Peiffer J, Brown PJ *et al.* Association Mapping: Critical Considerations Shift from Genotyping to Experimental Design. *Plant Cell* 2009;21:2194–2202.

42. Arend D, Junker A, Scholz U *et al.* PGP repository: a plant phenomics and genomics data publication infrastructure. *Database* 2016;2016:baw033.

43. Yuan ZH, El Hanafi S, Reif J. Diseases resistance and agronomic traits of 853 plant genetic resources and 344 European elite genotypes in multi-environments. 2024. doi: 10.5447/IPK/2024/7.

44. Li W, Cowley A, Uludag M *et al.* The EMBL-EBI bioinformatics web and programmatic tools framework. *Nucleic Acids Res.* 2015;43:W580–W584.

45. Scripts for 'High-quality phenotypic and genotypic dataset of barley genebank core-collection to unlock untapped genetic diversity'.2024. https://github.com/yzh1023/data-publication. Accessed on 19 December 2024.

46. Yuan Z; Rembe M; Mascher M; Stein N; Himmelbach A; Jayakodi M; Börner A; Oldach K; Jahoor A; Jensen JD; Rudloff J; Dohrendorf V; Kuhfus LP; Dyrszka E; Conte M; Hinz F; Trouchaud S; Reif JC; Hanafi SE: Supporting data for "High-quality phenotypic and genotypic dataset of barley genebank core-collection to unlock untapped genetic diversity" GigaScience Database. 2024. https://doi.org/10.5524/102638.

Manuscript with revision changes Click here to access/download;Manuscript;manuscript-revision_highlight-Yuan.et.al-20241202.docx

High-quality phenotypic and genotypic dataset of barley genebank core-collection to unlock untapped genetic diversity

Zhihui Yuan[1], Maximilian Rembe[1,2], Martin Mascher[1,3], Nils Stein[1,4], Axel Himmelbach[1], Murukarthick Jayakodi[1], Andreas Börner[1], Klaus Oldach[5], Ahmed Jahoor[6], Jens Due Jensen[6], Julia Rudloff[7], Viktoria-Elisabeth Dohrendorf[8], Luisa Pauline Kuhfus[9], Emmanuelle Dyrszka[9], Matthieu Conte[9], Frederik Hinz[10], Salim Trouchaud[11], Jochen C. Reif[1], Samira El Hanafi[1]

[1]Leibniz Institute of Plant Genetics and Crop Plant Research (IPK) Gatersleben, Seeland, Germany

[2]KWS SAAT SE & Co. KGaA, Grimsehlstr. 31, 37574 Einbeck, Germany

[3]German Centre for Integrative Biodiversity Research (iDiv) Halle-Jena-Leipzig, Leipzig, Germany

[4]Crop Plant Genetics, Institute of Agricultural and Nutritional Sciences, Martin-Luther-University of Halle-Wittenberg, Halle (Saale), Germany

[5]KWS LOCHOW GmbH, Ferdinand-von-Lochow-Str. 5, 29303 Bergen, Germany

[6]Nordic Seed Germany GmbH, Kirchhorster Str. 16 31688 Nienstädt, Germany

[7]Limagrain GmbH, Salderstr. 4, 31226 Peine-Rosenthal, Germany

[8]Nordsaat Saatzucht GmbH, Zuchtstation Gudow, Hofweg 8, D-23899 Gudow, Germany

[9]Syngenta France SAS, 12 Chemin de l'hobit, B.P. 27, 31790, Saint-Sauveur, France

[10]Saatzucht Bauer GmbH & CO.KG, Landshuter Straße 3a, 93083 Obertraubling, Germany

[11]Secobra Saatzucht GmbH, Feldkirchen 3, 85368 Moosburg an der Isar, Germany

corresponding author: Samira El Hanafi (hanafi@ipk-gatersleben.de)

Abstract

Background: Genebanks around the globe serve as valuable repositories of genetic diversity, offering not only access to a broad spectrum of plant material but also critical resources for enhancing crop resilience, advancing scientific research, and supporting global food security. To this end, traditional genebanks are evolving into bio-digital resource centres where the integration of phenotypic and genotypic data for accessions can drive more informed decision-

making, optimize resource allocation, and unlock new opportunities for plant breeding and research. However, the curation and availability of interoperable phenotypic and genotypic data for genebank accessions is still in its infancy and represents an obstacle to rapid scientific discoveries in this field. Therefore, effectively promoting FAIR, i.e. findable, accessible, interoperable, and reusable, access to these data is vital for maximizing the potential of genebanks and driving progress in agricultural innovation.

Findings: Here we provide whole genome sequencing data of 812 barley (*Hordeum vulgare* L.) plant genetic resources (PGRs) and 298 European elite materials released between 1949 and 2021, as well as the phenotypic data for four disease resistance traits and three agronomic traits. The robustness of the investigated traits and the interoperability of genomic and phenotypic data were assessed in the current publication, aiming to make this panel publicly available as a resource for future genetic research in barley.

Conclusions: The data showed broad phenotypic variability and high association mapping potential, offering a key resource for identifying genebank donors with untapped genes to advance barley breeding while safeguarding genetic diversity.

Keywords

Barley; plant genetic resources; elite; whole genome resequencing; disease resistance; agronomic traits

Data Description

Context

Successful plant breeding programs rely on balanced efforts between short-term goals to develop competitive cultivars and the maintenance of a broad genetic pool to guarantee long-term progress. In practice, the development of new varieties has been predominantly derived by recycling existing elite lines, leading to important genetic improvement and the reduction in the genetic diversity of elite germplasm. This could impede the breeding of potential new varieties capable of addressing and responding to constraints related to climate change, agronomical threads, and meeting the escalating social demands [1]. To overcome these limitations,

leveraging genetic diversity harbored within plant genetic resources (PGR) has been frequently suggested [2]. PGRs provide a valuable reservoir of untapped genetic potential that can be utilized to develop varieties with improved yield [3] and end-use quality, and enhanced resistance to both biotic and abiotic stresses, such as diseases [4], pests [5], waterlogging [6], salinity [7], and drought [6,8].

As the most cost-effective *ex situ* conservation strategy, genebanks worldwide are committed to maintaining PGRs, which hold a diverse gene pool encompassing all the alleles of various genes, including those from wild species, landraces, and breeding stocks. However, although enormous efforts have been made to conserve germplasm [9], it is estimated that less than 1% of the resources preserved in genebanks have been used in crop improvement [10]. The great challenge for breeders and scientists lies in finding useful barley PGRs among entire genebank collections that are comprised of thousands of accessions with complex patterns of genetic diversity [11]. Therefore, core collections were proposed as a strategy to streamline operational processes and mitigate costs, thereby facilitating more precise and effective research and breeding initiatives. Over the past decades, this approach has become even more attractive thanks to recent technological advancements, which have markedly reduced the costs of genotyping, and led to dramatic improvements in read length, sequencing chemistry, instrumentation, and throughput [12]. As a result, generating large-scale sequencing and genotyping datasets for entire genebank collections is now feasible. This has greatly expanded the scope of genotyping efforts and underpinned the effective selection of core collections that maximize genetic diversity [13]. These advancements provide powerful tools to efficiently harness PGRs, enabling the identification of valuable and favorable genes. This has streamlined their incorporation into crop improvement efforts, ultimately speeding up the development of new and improved varieties. Coupled with extensive and high-quality phenotypic data, the systematic use of whole genome sequencing data could provide valuable insights into genetic diversity and potential breeding opportunities. Our recent findings using genome-wide association analyses highlighted the value of these data in selecting donors with potentially novel favorable genes [14].

Moreover, the strategic deployment of core collections becomes even more compelling when combined with modern elite material [15,16], which serves as a reference panel to define favorable alleles/genes that are absent in the elite panel. This integrated approach is essential for enhancing polygenic traits and, hence, achieving informed pre-breeding decisions. To put this into practice, we selected a barley core collection [17] from the German Federal *ex situ* Genebank for Agriculture and Horticultural Crops at the Leibniz Institute of Plant Genetics and Crop Plant Research (IPK) and combined it with a set of European elite material. This population was designed to: i) phenotype the whole population in multi-environmental trials for three agronomical traits: plant height (PLH), heading date (HD), lodging (LOD), and four disease traits: *Puccinia hordei* (PUC), *Blumeria graminis hordei* (BLU), *Ramularia collo-cygni* (RAM), and *Rhynchosporium commune* (RHY); ii) evaluate the interoperability quality for the phenotypic and genomic datasets using five-fold cross-validation; iii) conduct the mantel test to check the detection power in association mapping analyses.

The data presented here can be further extended with additional PGRs and/or elite materials. It can also be integrated with alternative strategies to improve the utilization of germplasm collections by selecting untapped PGR donors, such as the development of novel association mapping methods. This will enable breeders to make more accurate predictions of trait performance, thereby enhancing the efficiency of selection processes. Furthermore, with the development of publicly accessible resources, scientists will be able to focus more on research and innovation, while reducing the burden of extensive phenotyping. The insights derived from our data may significantly accelerate advancements in genomic research and breeding programs, driving improvement and fostering future collaboration and resource sharing.

Methods

Barley material and field trials

To capture a broad spectrum of geographic origins and wide genetic diversity, we selected 812 PGRs which include 288 spring type (PGR_Spring) and 524 winter type (PGR_Winter), originating from 57 countries spanning 5 continents. Based on their performance during seed regeneration, these PGRs were thoughtfully selected from a previously described barley core

1000 collection [17], as a representative subset of the entire 21,405 barley accessions available at the IPK genebank [18], based on their performance during seed regeneration. Additionally, we incorporated 298 elite lines, including ten local checks, which consist of 128 spring type (Elite_Spring) and 170 winter type (Elite_Winter). These elites were exclusively selected from the European registered varieties and were available through the seed market, showcasing the breeding process over time from 1949 to 2021. The study initially included 87 additional genotypes which were later excluded from certain analyses due to incomplete phenotypic or genotypic data. To maintain the integrity of the dataset and facilitate accurate adjustments for experimental design effects, we retained all relevant data, including instances of missing information.

Field trials were conducted over three consecutive years (2020, 2021, and 2022) across eight locations in Germany: KWS-L/Prosselsheim (49°51'15.6"N, 10°06'04.1"E; 10.9ºC average annual temperature; 565.3 mm average annual rainfall); Nordic Seed/Nienstädt (52°17'35.52"N, 9°08'57.156"E; 10.7ºC average annual temperature; 638.4 mm average annual rainfall); Saatzucht Bauer/Riekofen (48°54'55.98"N, 12°21'21.744"E; 9.9ºC average annual temperature; 690.3 mm average annual rainfall); Limagrain/Peine-Rosenthal (52°18'09.828"N, 10°10'28.488"E; 10.9ºC average annual temperature; 607.5 mm average annual rainfall); Nordsaat/Gudow (53°33'28.0"N, 10°47'50.5"E; 10.4ºC average annual temperature; 581.1 mm average annual rainfall); Syngenta/Bad Salzuflen (52°04'21.576"N, 8°41'55.86"E; 10.5ºC average annual temperature; 692.8 mm average annual rainfall); Secobra-LEM/Lemgo (52°00'41.6"N, 8°52'22.7"E; 10.7ºC average annual temperature; 714.3 mm average annual rainfall); Secobra-FK/Moosburg (48°28'46.8"N, 11°54'32.6"E; 10.7ºC average annual temperature; 743.1 mm average annual rainfall). The trials were sown following a generalized alpha lattice design, which organizes genotypes into incomplete blocks to minimize spatial variation. Two-row observation plots (1 m^2) with two replications were used, and ten checks were included across years and locations for consistency. Each unique combination of year and location was considered as a distinct environment.

Phenotyping

The whole population was phenotyped for three agronomy traits for their importance in barley adaptability, yield potential, and harvestability: heading date measured in days from January 1st for winter type and from the sowing date onward for the spring type; plant height measured from the soil surface to the tip of spike in cm (excluding awns); and lodging rated on a 1-9 scale (with a higher score indicating severe lodging). Additionally, four disease traits including *Puccinia hordei*, *Blumeria graminis hordei*, *Ramularia collo-cygni*, and *Rhychnosporium commune* were evaluated under natural infection conditions. The disease severities were scored using an ordinal scale from 1 (fully resistant) to 9 (fully susceptible) following the guidelines of the German Federal Plant Variety Office [19].

Phenotypic data analyses

A linear mixed model using restricted maximum likelihood (REML) method [20] was used for data analyses across environments for spring and winter barley separately. Phenotypic data was corrected for outliers following the method of Tukey and Anscombe [21]. The residuals were extracted then normalized to flag the outliers according to a predefined significance threshold of p-value < 0.01 (Supplementary Table S2). Variance components and best linear unbiased estimations (BLUEs) of each genotype were computed from the oulier-corrected data following model (1):

$$y_{ijkm} = \mu + E_m + g_i + g_i \times E_m + E_m{:}r_j : b_k + e_{ijkm}, \qquad (1)$$

where y_{ijkm} denoted the vector of phenotypic values for i^{th} genotype (g) tested in k^{th} bock (b) nested in j^{th} replication (r) in mth environment (E), μ was the common mean, and e denoted the error term of the model. We assumed that all random effects followed an independent normal distribution with different variance components. In the model (1), all terms except μ and g_i were considered random for deriving the BLUEs across environments, whereas all terms except μ were modelled as random to estimate variance component for deriving heritability following model (2):

$$H^2 = \frac{\sigma_g^2}{\sigma_g^2 + \frac{\sigma_{g\times E}^2}{n_E} + \frac{\sigma_e^2}{n_R}}, \qquad (2)$$

where σ_g^2 denoted the genotypic variance, $\sigma_{g\times E}^2$ denoted the interaction between genotype and environment, σ_e^2 denoted the residual variance, and $\overline{n_R}$ denoted the average number of replications per genotype, $\overline{n_E}$ denotes the average number of environments in which the genotypes were evaluated. ASReml-R [22] was employed for all mixed linear models that were applied in the phenotypic analysis.

Whole genome shotgun sequencing

Whole genome sequencing (WGS) of the 1,110 genotypes (812 PGRs and 298 elite lines) was performed at IPK Gatersleben. High molecular weight (HMW) DNA was extracted from the leaves (8g) of greenhouse-grown (21°C/18°C day/night temperature) 7-day-old seedlings following a previously established protocol [23]. The Illumina Nextera libraries were prepared and sequenced using the Illumina NovaSeq 6000 platform [24]. Raw sequencing reads were trimmed using cutadapt [version 3.3; 25] and aligned to MorexV3 reference genome [26] using Minimap2 [version 2.20; 27]. The resultant alignment records were sorted with Novosort (V3.09.01; http://www.novocraft.com). Finally, a total of 149,380,812 single-nucleotide polymorphisms (SNPs) for the 1,110 genotypes were initially outputted by BCFtools [version 1.9; 28].

Quality control for SNP data

The resulting raw genotypic data was used to extract the corresponding datasets of the four sub-groups. Only bi-allelic SNPs with a minor allele frequency > 0.05 and missing rate < 0.1 were retained by PLINK [version 1.9; 29] for each of the four sub-groups. These meticulous steps yielded datasets comprising 17,759,260 SNPs for Elite_Spring, 26,903,811 for Elite_Winter, 54,934,336 for PGR_Spring, and 46,434,685 for PGR_Winter.

The resulting filtered genotypic data were used as input to phase and impute missing values using Beagle [version 5.2; 30], leveraging linkage disequilibrium to infer missing data accurately. Subsequently, an r^2 cutoff of 0.2 was set to prune markers by PLINK (version 1.9) with a sliding window size of 50 kb, and a step size of 10 kb. The final number of SNPs available differed in the four sub-groups due to the aforementioned process: 710,855 of Elite_Spring, 945,074 of Elite_Winter, 2,321,327 of PGR_Spring, and 1,775,972 of PGR_Winter. For each

tested SNP, homozygous for the most frequent allele, heterozygous, and homozygous for the alternative allele were coded as 0, 1, and 2 by PLINK (version 1.9), respectively.

Population structure

Subsequently, the aforementioned post-quality-control markers were used to investigate the population structure within and across spring and winter barley accessions using principal coordinate analysis (PCoA) based on pairwise Rogers' distance [31]. PCoA was performed using the R package ape [version v5.7-1; 32]. Additionally, the population structure was tested using ADMIXTURE [version 1.3.0; 33]. The optimal number of population components was determined based on cross-validation function (--cv).

Moreover, linkage disequilibrium (LD) analyses of the four sub-groups was carried out separately by determining the pairwise squared allele-frequency correlations (r^2) between markers [34] and then combined to estimate LD decay across the entire genome. A decay curve was fitted for each sub-group using nonlinear regression of pairwise r^2 against the distance (Mb) between the markers. LD within a specific physical distance of 2 Mb was calculated and visualized using PopLDdecay [version 3.40; 34].

Genomic-phenotypic data interoperability

To evaluate the interoperability for the phenotypic and genomic datasets, we calculated the accuracy of the genomic best linear unbiased prediction (GBLUP) [35]. First, the mixed model equations for genomic prediction were computed using REML in the rrBLUP R package [v4.6.1; 36]. Prediction accuracies were then estimated through five-fold cross validation. In this process, both phenotypic and genomic datasets were randomly subdivided into five groups. The first four groups served together as the training set, whereas the fifth group corresponded to the prediction set. The random sampling was repeated 100 times, giving a total of 500 cross-validation runs. Genomic prediction ability was thereafter defined as the correlation between BLUEs across environments for a trait and the corresponding predicted values.

Mantel correlation

Following the imputation process, we used PLINK (version 1.9) to construct a genetic relationship matrix. To further explore the association between phenotypic variation and

population structure, the correlation between the genetic relationship matrix and the absolute trait differences (Euclidean distance matrix) in each sub-group was tested using a Mantel test [38] implemented in the R package vegan [v2.6-4; 36] , and visualized by linkET R package [v0.0.7.4; 37], 999 permutations were used to evaluate the significance of the test.

Data Validation and quality control

High heritability estimation highlights the robustness of the phenotypic data

The quality and reliability of the phenotypic data were rigorously assessed by estimating the heritability of the evaluated traits. After outlier correction, the heritability estimates for most traits were generally high, exceeding 0.5 (Fig. 1A). Notable exceptions included RHY in the spring population (h^2 = 0.05), and RAM (h^2 = 2E-06) in the winter population. Variance components analysis revealed that environment (σ_e^2) accounts for the largest proportion of the total variance, while genotype and genotype × environments interaction were less pronounced, with the exception of LOD and RHY in both spring and winter population, as well as PUC in winter population (Fig. 1B). This suggests that factors such as temperature fluctuations, varying levels of precipitation, and humidity across different climate zones may have influenced the observed phenotypic performance. These environmental conditions likely influenced growth patterns and trait expression, leading to larger phenotypic variability in traits with high heritability and restricted variability in traits with low heritability.

The resulting BLUEs showed normal distribution for most disease traits (Fig. 2). However, RHY showed left skew in both spring and winter population, while BLU and PUC displayed left skew in elite population for both spring and winter type. The left skew of RHY suggests low disease pressure across three years and, hence, resulting in a small proportion of susceptible genotypes. And the left skew of elite population of BLU and PUC suggests that PGRs tend to be more susceptible than the elite materials for the two diseases. For agronomic traits (Fig. 3), PGR population showed normal distribution, while elite lines showed normal distribution in HD and PLH only in winter population.

Furthermore, several significant correlations were observed between the evaluated traits (Fig. 4). For pairing of agronomic and disease traits, it was observed that HD was negatively

correlated with all the disease traits, except for BLU in winter barley population. Those observations suggest a strategic plant response given that delayed heading allows plants to evade disease infection through spatial or temporal adjustments. Moreover, LOD was positively correlated with all the disease traits, except for RAM in spring barley population. PLH was positively correlated with BLU and PUC while negatively correlated with RAM and RHY.

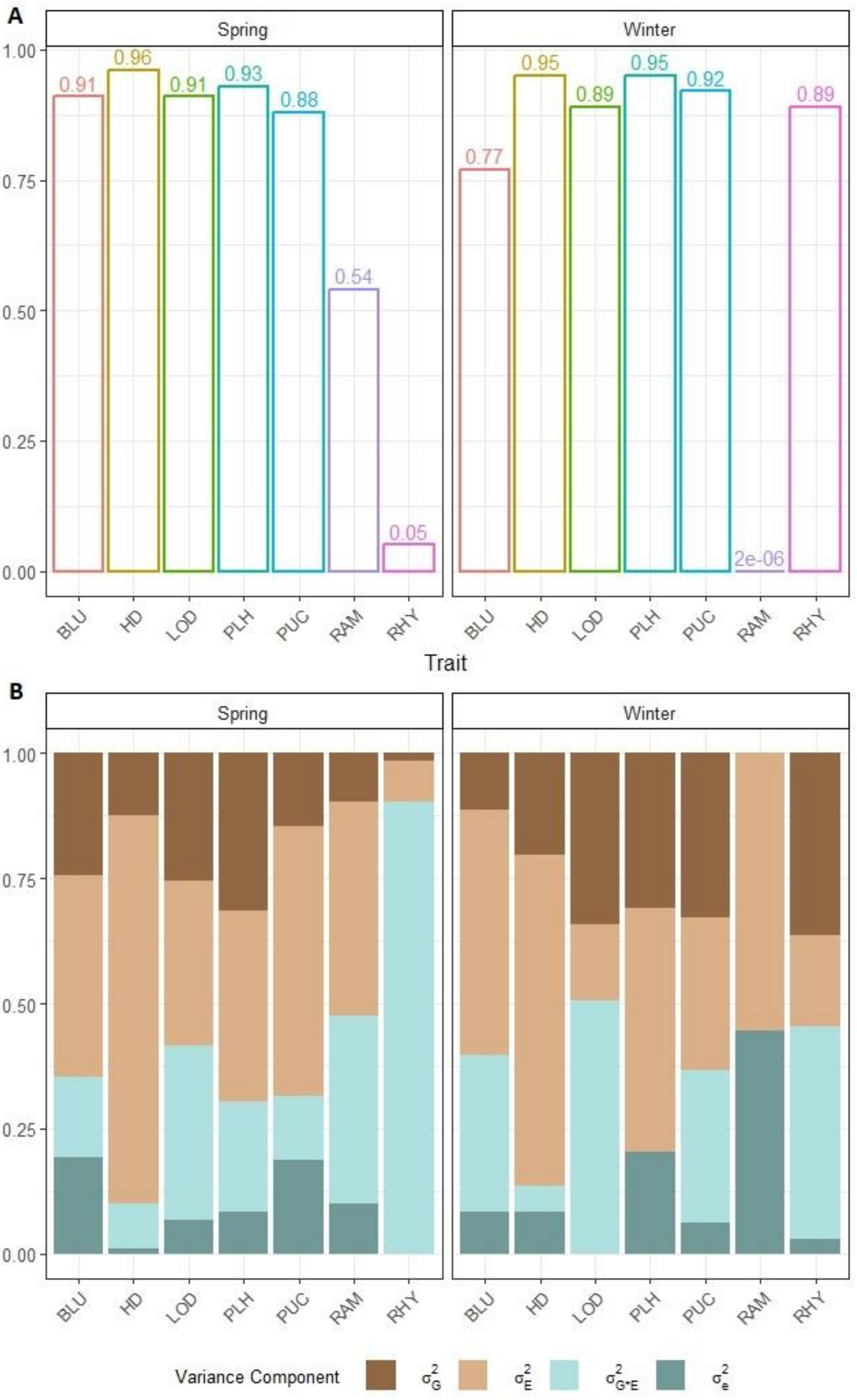

Figure 1: Heritability (A) and percentages of the different variance components (B) for the seven traits considered in this study. BLU: *Blumeria graminis hordei*; PUC: *Puccinia hordei*; RHY: *Rhynchosporium commune*; RAM: *Ramularia collo-cygni*; HD: heading date; PLH: plant height; LOD: lodging; σ^2_G: genotypic variance; σ^2_{G*E}: variance due to genotype by environment interaction; σ^2_E: variance due to environment; σ^2_e: residual.

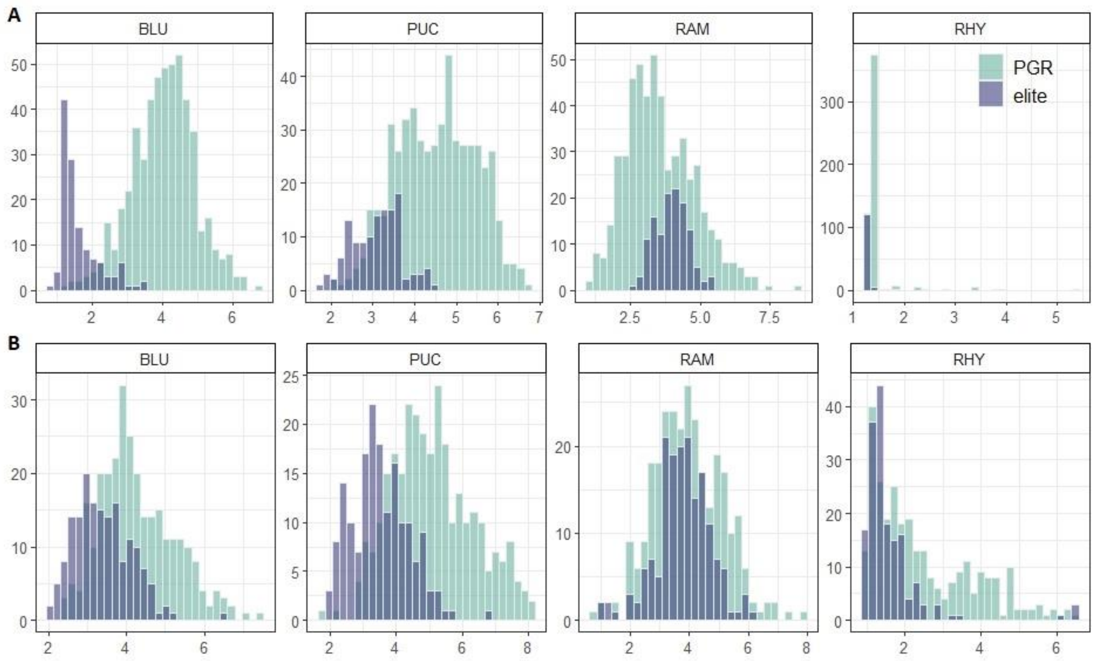

Figure 2: Histogram showing the phenotypic distribution for four diseases traits for spring (A) and winter (B) population. BLU: *Blumeria graminis hordei*; PUC: *Puccinia hordei*; RHY: *Rhynchosporium commune*; RAM: *Ramularia collo-cygni*.

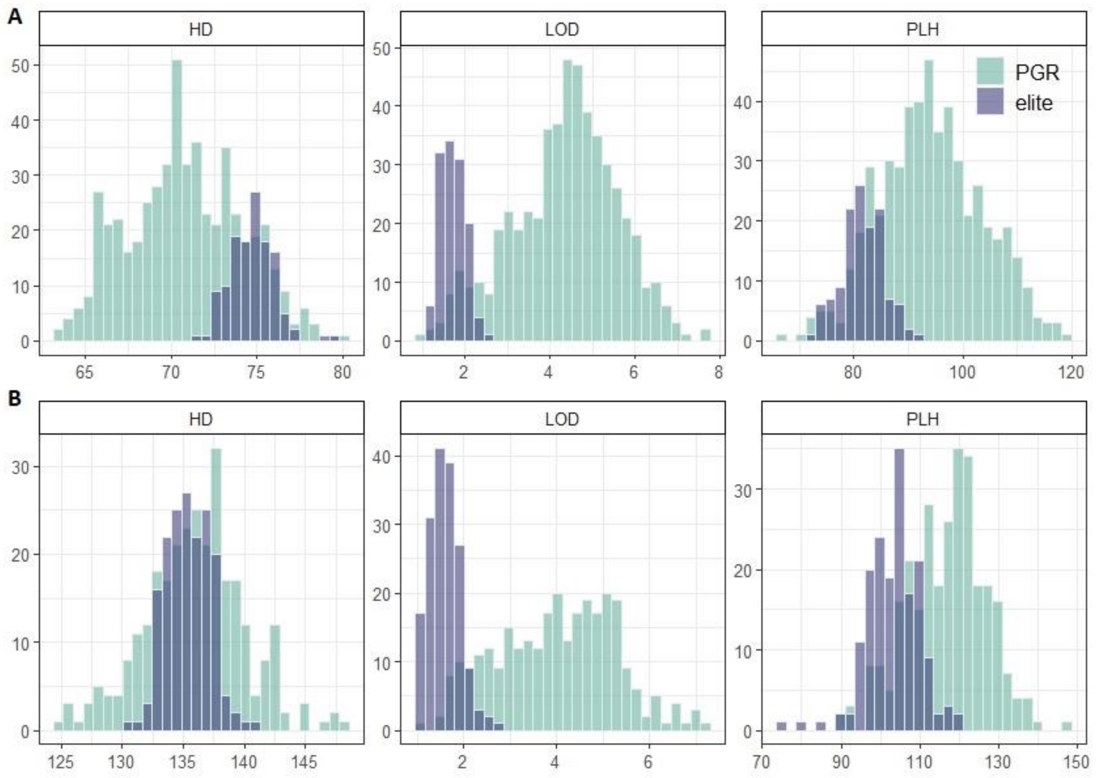

Figure 3: Histogram showing the phenotypic distribution for three agronomic traits for spring (A) and winter (B) population. HD: heading date; PLH: plant height; LOD: lodging.

Whole genome sequencing data showed high genetic diversity and high marker densities

Whole genome sequencing (WGS) data of the 1,110 genotypes showed an average coverage of 4.7x with a range spanning from 0.5x to 22.6x across all samples with mapping rate from 94% to 99%, providing a solid foundation for downstream genetic analyses and ensuring a comprehensive representation of the genomic information across the diverse set of genotypes. Building on this comprehensive genomic dataset, we performed PCoA to assess the genetic diversity among the spring and winter barley population as reported in our companion study [14]. The first two coordinates explained together 11.66% and 11.25% of the spring and winter population, respectively. As anticipated, the inclusion of PGRs significantly broadened the genetic diversity compared to the elite materials. Notably, the elite spring population formed a tight, cohesive cluster indicating less genetic diversity, while the elite winter population exhibited a more dispersed pattern reflecting greater genetic variability.

To further complement the population structure analyses, the optimal number of genetic components (K = 3) was determined based on cross-validation results. The admixture analysis revealed distinct population structures within the spring and winter populations (Fig. 4), with individuals showing varying proportions of the three inferred components. These results highlight the contrasting levels of genetic diversity and population structure within each spring and winter barley genotypes.

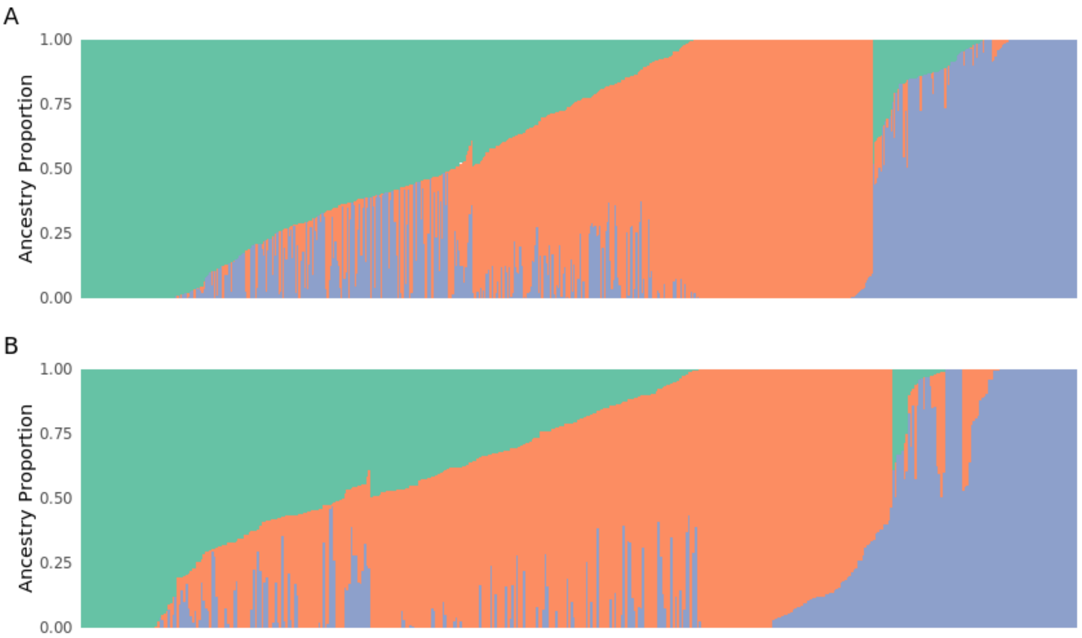

Figure 4: Admixture analysis of spring (A) and winter (B) populations with the K = 3 admixture model. Each individual is represented as a vertical bar with colour corresponding to the proportions of three ancestral components (K).

For the intra-chromosomal decay of LD (r^2), PGR was faster in both spring and winter population as compared to elite materials. The slower LD decay in elite population may be due to genetic bottlenecks and/or high selection pressures that produce specific linkage between alleles that control specific phenotypes.

High genomic prediction accuracies support the interoperability of genomic and phenotypic data

Systematic errors can occur during field trials, which will systematically disrupt the connectivity between genotype and phenotype data and, in turn, decrease the value of the data for subsequent integrated analyses. To assess potential data imbalances, we used the cross-validated accuracy of genomic prediction as a quality measure for genomic-phenotypic data interoperability.

Integrating phenotypic data with WGS data resulted in 652 spring and 458 winter barley genotypes. Overall, the genomic-phenotypic data interoperability was in general high (Fig. 5), with a maximum prediction accuracy observed for lodging in both spring and winter populations. Disease-resistant traits showed moderate to high prediction abilities, suggesting

that genomic data can be reliably used, thereby potentially accelerate breeding efforts for resistant varieties. In parallel, this robust result ensures reliable data quality, enabling comprehensive analyses to explore genotype-phenotype relationships, and lay a solid foundation for future studies aimed at finding marker-trait associations and understanding the genetic mechanisms underlying key traits in barley.

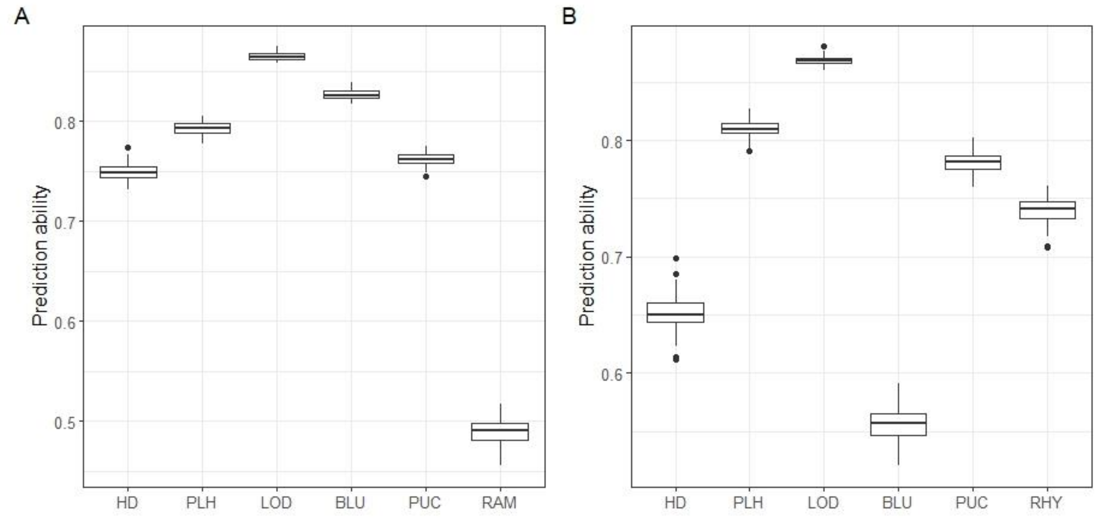

Figure 5: Five-fold cross-validation abilities of the genomic best linear unbiased prediction for heading date (HD; days); plant height (PLH; cm); lodging (LOD); *Blumeria graminis hordei* (BLU); *Puccinia hordei* (PUC); *Rhynchosporium commune* (RHY); *Ramularia collo-cygni* (RAM), obtained in the spring (A) and winter (B) populations.

Mantel test results indicate a high detection power in association mapping

Accurate mapping requires addressing the complexities inherent in genetic relatedness among individuals. In such way, especially when dealing with panels comprising both elite lines and PGRs, the intricate patterns of genetic relationship can pose significant challenges. Specially, when phenotype variation is influenced by genetic relatedness, it becomes crucial to differentiate between genuine associations and those resulting from shared genetic backgrounds. This complexity underscores the importance of robust methods, to effectively uncover meaningful correlations and enhance the reliability of association mapping. Therefore, by minimizing genotype-phenotype covariance, we can reduce the risk of spurious associations [41]. Mantel test is a widely used approach to examine the association between two matrices. The results revealed a moderate to low correlation between genetic distance and Euclidean

phenotypic distance matrix, indicating a lack of strong association between phenotypic variation and genome-wide genetic differences (Fig. 6; Mantel's r in spring barley ranged from -0.02 to 0.29, and from 0 to 0.32 in winter barley), which in turn expected to increase the detection power in association mapping.

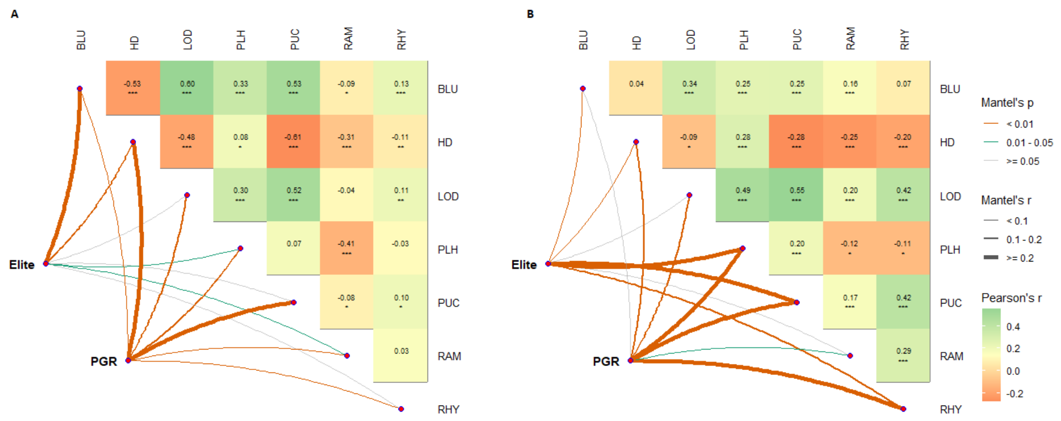

Figure 6: Pairwise correlations for the recorded traits, and the Mantel tests between tested traits vs. elite materials and plant genetic resources (PGR) for spring barley (A) and winter barley (B). The lines represent significant relationships, where the width of the line represents the Mantel r statistic value and the different colours of the lines represent different degrees of significance. The Pearson correlation coefficient between different traits is shown in the heat map matrix. BLU: *Blumeria graminis hordei*; PUC: *Puccinia hordei*; RHY: *Rhynchosporium commune*; RAM: *Ramularia collo-cygni*; HD: heading date; PLH: plant height; LOD: lodging; *** p < 0.001, ** p < 0.01, * p < 0.05.

Data Availability

Phenotypic records: The raw phenotypic data described here as well as the ready-to-use phenotypic values (BLUEs), and the R script to import and curate the raw phenotypic data to compute heritability and BLUEs are available in the e!DAL-PGP Repository [42] and can be directly accessed here [43].

Raw sequencing reads: FASTQ files containing raw reads for 1,110 genotypes were submitted by [24], and deposited at the European Nucleotide Archive [44] under BioProjects PRJEB53924 (Illumina resequencing data). Sequenced genotypes are findable through their 'SAMEA' IDs. The integrated Elite and PGR 'SAMEA' BioSample IDs connected with plant material

passports, passport data sources, SSD- and IPK genebank DOIs were listed in Supplementary Table S1.

SNP markers: variant calling results based on read mapping against the reference sequence of MorexV3 were stored as Variant Call Format (VCF). All the VCF files are located at the European Nucleotide Archive under the project number PRJEB80159.

The script for filtering VCF files, imputation, admixture process, mantel test, and cross validation is accessible at https://github.com/yzh1023/data-publication.git.

Additional files

Supplementary Table S1. List of 1,110 genotypes in this dataset.

Supplementary Table S2. The number and proportion of outliers identified for each trait.

Abbreviations

BLU: *Blumeria graminis hordei*; BLUE: best linear unbiased estimations; HD: heading date; IPK: Institute of Plant Genetics and Crop Plant Research; LD: linkage disequilibrium; LOD: lodging; PCoA: principal coordinate analysis; PGR: plant genetic resources; PLH: plant height; PUC: *Puccinia hordei*; RAM: *Ramularia collo-cygni*; RHY: *Rhynchosporium commune*; SNP: single-nucleotide polymorphisms; WGS: whole genome sequencing.

Author's Contributions

KO, AJ, JDJ, JR, VD, LPK, ED, MC, FH, and ST: cultivation and provision of phenotypic data of all spring and winter barleys over 3 years in one to two locations; ZY: genotypic data analyses and curation; MR and SEH: phenotypic data analyses; MM, MJ, AH, and NS: generated and processed the genomic data; NS, MM, AB, SEH, and JCR: edited and revised the manuscript; AB: developed the core 1000 population; JCR, NS, SEH, and ZY: designed the study; ZY and SEH: wrote the paper. All authors read and approved the final manuscript.

Funding

This research work is funded by German Ministry of Food and Agriculture under the project Structural genome variation, haplotype diversity and the barley pan-genome - Exploring

structural genome diversity for barley breeding (SHAPE) phase 1 and 2 (BMBF FKZ 031B0190A; 031B0884A).

Competing Interests

No conflict of interest declared.

Acknowledgement

We are grateful for the technical assistance of Mary Ziems and Annette Marlow for providing seeds of plant material. Susanne König, and Ines Walde for technical assistance during sequencing data production, as well as Anne Fiebig, Daniel Arend and Matthias Lange for support with data management and submission to repositories.

References

1. Ellegren H, Galtier N. Determinants of genetic diversity. *Nat Rev Genet.* 2016;17:422–433.

2. Halewood M, Chiurugwi T, Sackville Hamilton R *et al.* Plant genetic resources for food and agriculture: opportunities and challenges emerging from the science and information technology revolution. *New Phytologist* 2018;217:1407–1419.

3. Dillon SL, Shapter FM, Henry RJ *et al.* Domestication to Crop Improvement: Genetic Resources for *Sorghum* and *Saccharum* (Andropogoneae). *Annals of Botany* 2007;100:975–989.

4. Deng Y, Ning Y, Yang D *et al.* Molecular Basis of Disease Resistance and Perspectives on Breeding Strategies for Resistance Improvement in Crops. *Molecular Plant* 2020;13:1402–1419.

5. Radchenko EE, Abdullaev RA, Anisimova IN. Genetic Resources of Cereal Crops for Aphid Resistance. *Plants* 2022;11:1490.

6. Valliyodan B, Ye H, Song L *et al.* Genetic diversity and genomic strategies for improving drought and waterlogging tolerance in soybeans. *J Exp Bot.* 2016;68:1835–1849.

7. Razzaq A, Saleem F, Wani SH *et al.* De-novo Domestication for Improving Salt Tolerance in Crops. *Front Plant Sci.* 2021;12:681367.

8. Missanga JS, Venkataramana PB, Ndakidemi PA. Recent developments in *Lablab purpureus* genomics: A focus on drought stress tolerance and use of genomic resources to develop stress-resilient varieties. *Legume Science* 2021;3:e99.

9. Wambugu PW, Ndjiondjop M-N, Henry RJ. Role of genomics in promoting the utilization of plant genetic resources in genebanks. *Briefings in Functional Genomics* 2018;17:198–206.

10. Sharma S, Upadhyaya HD, Varshney RK *et al.* Pre-breeding for diversification of primary gene pool and genetic enhancement of grain legumes. *Front Plant Sci* 2013;4:309.

11. Odong TL, Jansen J, van Eeuwijk FA *et al.* Quality of core collections for effective utilisation of genetic resources review, discussion and interpretation. *Theor Appl Genet.* 2013;126:289–305.

12. Salgotra RK, Chauhan BS. Genetic Diversity, Conservation, and Utilization of Plant Genetic Resources. *Genes* 2023;14:174.

13. El Hanafi S, Jiang Y, Kehel Z *et al.* Genomic predictions to leverage phenotypic data across genebanks. *Front Plant Sci.* 2023;14:1227656.

14. Yuan ZH, Rembe M, Mascher M *et al.* Capitalizing genebank core collections for rare and novel disease resistance loci to enhance barley resilience. *Journal of Experimental Botany* 2024:erae283.

15. Cazenave X, Petit B, Lateur M *et al.* Combining genetic resources and elite material populations to improve the accuracy of genomic prediction in apple. *G3 Genes|Genomes|Genetics* 2022;12:jkab420.

16. Sehgal D, Vikram P, Sansaloni CP *et al.* Exploring and Mobilizing the Gene Bank Biodiversity for Wheat Improvement. *PLoS ONE* 2015;10:e0132112.

17. Milner SG, Jost M, Taketa S *et al.* Genebank genomics highlights the diversity of a global barley collection. *Nat Genet.* 2019;51:319–326.

18. Oppermann M, Weise S, Dittmann C *et al.* GBIS: the information system of the German Genebank. *Database* 2015;2015:bav021.

19. Bundessortenamt. Richtlinien für die Durchführung von landwirtschaftlichen Wertprüfungen und Sortenversuchen. 2000.

20. Patterson HD, Thompson R. Recovery of inter-block information when block sizes are unequal. *Biometrika* 1971;58:545–554.

21. Anscombe FJ, Tukey JW. The Examination and Analysis of Residuals. *Technometrics* 1963;5:141–160.

22. Butler DG, Cullis BR, Gilmour AR *et al.* ASReml estimates variance components under a general linear. 2023.

23. Dvorak J, McGuire PE, Cassidy B. Apparent sources of the A genomes of wheats inferred from polymorphism in abundance and restriction fragment length of repeated nucleotide sequences. *Genome* 1988;30:680–689.

24. Jayakodi M, Lu Q, Pidon H *et al.* Structural variation in the pangenome of wild and domesticated barley. *Nature* 2024. https://doi.org/10.1038/s41586-024-08187-1.

25. Martin M. Cutadapt removes adapter sequences from high-throughput sequencing reads. *EMBnet.journal* 2011;17:10.

26. Mascher M, Wicker T, Jenkins J *et al.* Long-read sequence assembly: a technical evaluation in barley. *The Plant Cell* 2021;33:1888–1906.

27. Li H. Minimap2: pairwise alignment for nucleotide sequences. *Bioinformatics* 2018;34:3094–3100.

28. Li H. A statistical framework for SNP calling, mutation discovery, association mapping and population genetical parameter estimation from sequencing data. *Bioinformatics* 2011;27:2987–2993.

29. Purcell S, Neale B, Todd-Brown K *et al.* PLINK: A Tool Set for Whole-Genome Association and Population-Based Linkage Analyses. *The American Journal of Human Genetics* 2007;81:559–575.

30. Browning BL, Zhou Y, Browning SR. A One-Penny Imputed Genome from Next-Generation Reference Panels. *The American Journal of Human Genetics* 2018;103:338–348.

31. Rogers JS. Measures of genetic similarity and genetic distance. *Studies in genetics VII* 1972:145–153.

32. Paradis E, Schliep K. ape 5.0: an environment for modern phylogenetics and evolutionary analyses in R. Schwartz R (ed.). *Bioinformatics* 2019;35:526–528.

33. Alexander DH, Novembre J, Lange K. Fast model-based estimation of ancestry in unrelated individuals. *Genome Res.* 2009;19:1655–1664.

34. Hill WG, Robertson A. Linkage Disequilibrium in Finite Populations. *Theoretical and Applied Genetics* 1968;38:226–231.

35. Zhang C, Dong SS, Xu JY *et al.* PopLDdecay: A fast and effective tool for linkage disequilibrium decay analysis based on variant call format files. *Bioinformatics* 2019;35:1786–1788.

36. VanRaden PM. Efficient Methods to Compute Genomic Predictions. *Journal of Dairy Science* 2008;91:4414–4423.

37. Endelman JB. Ridge Regression and Other Kernels for Genomic Selection with R Package rrBLUP. *The Plant Genome* 2011;4:250–255.

38. Mantel N. The detection of disease clustering and a generalized regression approach. *Cancer Res.* 1967;27:209–220.

39. Oksanen J, Simpson GL, Blanchet FG *et al.* vegan: Community Ecology Package. 2022.

40. Huang H. LinkET: everything is linkable. *R package version 0.0.7.4.* 2021;3.

41. Myles S, Peiffer J, Brown PJ *et al.* Association Mapping: Critical Considerations Shift from Genotyping to Experimental Design. *Plant Cell* 2009;21:2194–2202.

42. Arend D, Junker A, Scholz U *et al.* PGP repository: a plant phenomics and genomics data publication infrastructure. *Database* 2016;2016:baw033.

43. Yuan ZH, El Hanafi S, Reif J. Diseases resistance and agronomic traits of 853 plant genetic resources and 344 European elite genotypes in multi-environments. 2024. doi: 10.5447/IPK/2024/7.

44. Li W, Cowley A, Uludag M *et al.* The EMBL-EBI bioinformatics web and programmatic tools framework. *Nucleic Acids Res.* 2015;43:W580–W584.

Manuscript response to reviewers Click here to access/download;Manuscript;response_to_reviewers.docx

Manuscript GIGA-D-24-00417

Response to reviews

Dear Dr. Nogoy,

Thank you for considering our manuscript for publication in *GigaScience*. We appreciate the time and effort that you and the reviewers have taken to provide feedback on our manuscript, and we are grateful for the insightful comments and constructive suggestions. We have carefully addressed the points raised and incorporated them into the revised manuscript. The changes are highlighted in yellow in the revised version for easy reference. We have also made minor edits throughout the manuscript to improve readability and clarity. The adjustments do not alter the content or conclusions of the paper, but enhance the overall given information. We hope that these revisions substantially strengthen our manuscript and believe that it now meets the standards for publication in *GigaScience*.

Reviewer1 Comments to the Author:

This data note presents a comprehensive study on the integration of genebank resources with precision phenotyping and genotyping to enhance barley breeding. The authors have effectively highlighted the significance of genetic diversity in barley and the role of genebanks in supporting agricultural innovation. The topic is highly relevant to current challenges in agriculture, particularly in the context of food security and climate change. Although the study carries significant strengths making it qualified for publication in GigaScience but needs some reconsiderations to resolve some concerns issues before going to formal acceptance for publication and are given as under. The study has the potential to make a significant contribution to the field of barley breeding and genomics. With revisions addressing the identified areas for improvement, this note could be a strong candidate for publication.

Authors' response: Thank you for your thorough review and valuable feedback. We appreciate the time and effort you have invested in evaluating our work. We have addressed each of your comments and suggestions in detail below. Changes have been made and highlighted accordingly in the manuscript.

As an article of "Data Note", the title is overstated. The title should focus more on the data itself.

Authors' response: Thank you for your feedback. We appreciate your insight regarding the title of the manuscript. Based on the nature of a "Data Note" article, we have changed our title to "High-quality phenotypic and genotypic dataset of barley genebank core-collection to unlock untapped genetic diversity".

Summarize the main conclusions drawn from the results succinctly in the abstract.

Authors' response: We appreciate your suggestion. A brief conclusion has been included in the abstract. Please refer to line 41-43.

Try to add more background information on barley breeding and its challenges particularly emphasize the relevance of the research in the current agricultural landscape.

Authors' response: Thank you for your valuable feedback. We appreciate your suggestion to provide more background information on barley breeding and its challenges. We have expanded the introduction to include a short discussion of barley breeding, highlighting its significance in global importance. Specifically, we outlined the key challenges faced by breeders, such as adapting barley to changing climatic conditions, improving disease resistance, and enhancing yield stability (please refer to lines 53-55 and lines 57-60).

Additionally, we emphasized the relevance of our research within the context of the current agricultural landscape, given the increasing demand for leveraging the genetic diversity harbored within plant genetic resources. We also discuss how advances in genomics and phenotyping, such as tools explored in our study, can contribute to addressing these challenges and improving the efficiency of barley breeding programs.

We believe these additions help contextualize our research and demonstrate its importance in advancing barley breeding to meet future agricultural demands. Thank you again for your insightful suggestion.

State the specific objectives of the study more explicitly and in a separate paragraph and briefly outline the structure of the manuscript at the end of this section.

Authors' response: Thank you for your constructive suggestion. We have revised the context to include a distinct part that explicitly states the specific objectives of the study. This paragraph now clearly outlines our aims. Please refer to lines 91-96.

It is suggested to summarize/describe the environmental conditions under which the trials were conducted.

Authors' response: Thank you for your valuable feedback. We have added a detailed summary of the environmental conditions under which the trials were conducted. This section now describes the average temperature across three years for each location. Please refer to lines 123-136.

Can you please provide justification for the choice of traits and methods used in the study and elaborate on the phenotyping methods, including any specific protocols followed.

Authors' response: Thank you for your insightful comments. We appreciate the opportunity to provide further justification for the choice of traits and methods used in this study.

The agronomic traits (heading date, plant height, and lodging) were selected for their importance in barley adaptability, yield potential, and harvestability, while disease traits address key breeding challenges for durable resistance. Phenotyping followed standardized protocols: heading date as days to 50% heading from January 1st for winter type and from the sowing date for the spring type, plant height measured at maturity, and lodging scored visually. The four disease traits [*Puccinia hordei* (PUC), *Blumeria graminis hordei* (BLU), *Ramularia collo-cygni* (RAM), and *Rhychnosporium commune* (RHY)] were included because these diseases are prevalent in the study's geographic region and are known to significantly affect crop health and yield. Disease resistance was assessed under field conditions to ensure robust and reproducible data, directly linking genebank materials to breeding applications.

We have integrated your comment in the revised manuscript to enhance transparency and to ensure that the methods used are well-justified. Please refer to line 140-148.

Provide some details on the alpha lattice design used in the trials and try to add a description of the statistical methods used for data analysis along with significance level of results obtained.

Authors' response: Thank you for your valuable feedback. We appreciate your suggestion to provide more details on the alpha lattice design and statistical methods used in the trials. A detailed description of the alpha lattice design employed in our trials is available in the reference [40]. For clarity, we have included a brief summary of the design in the revised manuscript, ensuring that key elements (please refer to lines 133-137). Regarding the statistical methods, we have included a detailed description of the analyses performed in the "Phenotypic data analyses" section, please refer to lines 149-168.

Clarify the criteria used for selecting SNPs for analysis besides providing more details on the imputation methods used for missing data.

Authors' response: We appreciate your attention to detail. To address this, we have added a sentence in the manuscript specifying the criteria for SNP selection. These criteria include minor allele frequency > 0.05 and missing rate < 0.1, and an r^2 cutoff of 0.2 was set to prune markers.

Regarding the imputation methods, Beagle was chosen due to its reliability in phasing and imputing genotypes, leveraging linkage disequilibrium to infer missing data accurately. The process involved phasing the genotypes and imputing missing data. Please refer to lines 186-188.

Given the limitations of Principal Coordinates Analysis (PCoA) in addressing population structure and ancestry issues, could you clarify the rationale behind choosing PCoA instead of utilizing methods like Admixture or STRUCTURE, which are specifically designed to provide insights into these aspects?

Authors' response: Thank you for your insightful question regarding the choice of Principal Coordinate Analysis (PCoA) over methods like Admixture or STRUCTURE for this study. To complement our analyses, we have add the admixture results into the manuscript. Please refer to lines 276-284.

PCoA reduces a multi-dimensional dataset to a much smaller number of dimensions that allows for visual exploration and compact quantitative summaries. With admixture or STRUCTURE proportion inference, individuals in a sample are modeled as having a proportion of their genome derived from each of several source populations. The goal is to infer the proportions of ancestry from each source population, and these proportions can be used to produce compact visual summaries that reveal the existence of population structure in a sample. Another qualitative difference is that PCoA produces consistent results as more dimensions are added, whereas admixture-based methods produce qualitatively different results with different numbers of genetic components (K). Although consistency may seem a desirable property, there can be benefits to the different perspectives obtained by using different numbers of factors. In our study, the optimal number of genetic components (K) was tested using admixture based on cross-validation function (--cv), please refer to lines 198-200.

This note mentions the integration of genotypic and phenotypic data but does not provide a clear framework or methodology for how this integration was/would be achieved or its significance. Include a section that explicitly describes the integration process of genotypic and phenotypic data. Discuss the challenges faced/would be faced during integration and how they could be addressed. Highlight the importance of this integration for future breeding programs.

Authors' response: Thank you for your insightful feedback. The quality check of the integration of genotypic and phenotypic data in our study was achieved by genome-wide prediction coupled with 100 times five-fold cross-validation. Overall, the genomic-phenotypic data interoperability was in general high (please refer to lines 288-307). Additionally, genome-wide association studies were used to identify genetic markers associated with the trait, providing actionable insights for breeding decisions (please refer to reference [14]).

A key challenge in this integration process was handling large-scale datasets with missing genotypic values. To address this, we employed phasing and imputation with Beagle to ensure high-quality genotype data, followed by filtering for minor allele frequency and missing rates to enhance reliability. As for the phenotypic data, standardized field trials and precise trait phenotyping minimized environmental noise.

The integration of genotypic and phenotypic data is critical for future breeding programs, offering a framework to validate markers identified through GWAS and use them in marker-assisted selection to identify potential donors for traits of interest. Moreover, by linking genetic diversity with key agronomic traits, breeders can make more informed decisions by generate more accurate predictions about trait performance, improving the efficiency of variety development and accelerating breeding cycles. Please refer to lines 79-83.

Improve the narrative flow by logically grouping related findings together.

Authors' response: Thank you for your valuable feedback. We have carefully reviewed the structure of the manuscript and have made revisions to improve the narrative flow. Specifically, we've provided clearer transitions between sections to help the reader follow the flow of the most critical findings. We hope these changes enhance the clarity and readability of the manuscript.

Emphasize the most important findings in the text to guide the reader.

Authors' response: Thank you for your valuable feedback. Key results are now highlighted in the sub-title to guide the reader. We've ensured that the central conclusions are presented in a way that underscores their significance in the context of the study's objectives.

Briefly mention any limitations observed in the results. Acknowledge any limitations of the study and their potential impact on the findings.

Authors' response: Thank you for your insightful feedback. We have acknowledged the limitations of our study in the revised manuscript. One key limitation is the relatively low heritability observed for RHY in spring population and RAM in the winter population. The low disease pressure resulted in limited phenotypic variation and reduced heritability, as the environmental conditions did not sufficiently differentiate the responses of the genotypes. This diminished the ability to detect genetic contributions to the two traits.

I think authors should consider to clearly articulate the practical implications of the findings for barley breeding or agricultural practices. If their findings fail to connect to real-world applications, the relevance of the study may be questioned.

Authors' response: Thank you for bringing this to our attention. This integrated approach also enables breeders to identify genetic markers associated with desirable traits, accelerating the development of crops with improved yield, disease resistance, or other key attributes. By combining genotypic and phenotypic data, breeders can also make more accurate predictions about trait performance, ultimately improving the efficiency of selection processes. We believe our dataset will underscore the importance of this approach in advancing breeding programs. Please refer to line 97-103.

Briefly add how environmental conditions may have influenced phenotyping results.

Authors' response: Thank you for this valuable suggestion. We have updated the section to include a brief explanation of how non-conductive environmental conditions may have influenced the phenotyping results. Specifically, we acknowledge that variations in temperature, humidity, and precipitation across the different climate zones could impact the expression of the trait under study. For example, stress from extreme temperatures or inconsistent rainfall may alter plant growth or other phenotypic traits, potentially affecting data consistency and interpretation. Please refer to line 233-237.

Briefly add potential biological mechanisms underlying the observed traits.

Authors' response: Thank you for your feedback and for bringing this to our attention. We appreciate the importance of discussing potential biological mechanisms for additional context. However, the primary scope of this paper is centered on the description of the dataset. A detailed examination of biological mechanisms would extend beyond the intended focus and depth of our work. Keeping the manuscript concise and aligned with our original objectives is essential to its clarity.

Integrate key points from the findings to reinforce the conclusions drawn.

Authors' response: Thank you for your valuable suggestion. We have revised the conclusion section to better integrate key points from the findings and reinforce the conclusions drawn from our study. Please refer to line 41-43.

Reviewer2 Comments to the Author:

The manuscript by Yuan et al describes the public release of phenotypic and genotypic data of a large set of barley landraces, genebank accessions, and elite materials. The authors provide a comprehensive dataset, whole genome sequencing data together with phenotypic data for disease and agronomic traits, that will be of interest for future genetic research in barley. The document is well written, highlighting the value of the data provided. I have some comments or suggestions to further improve the document, as follows:

Authors' response: We sincerely thank you for your thoughtful review and valuable feedback on our manuscript. We appreciate your recognition of the importance of the issues addressed in our study and your acknowledgment of the manuscript's quality.

Line 64: "enormous efforts that have been made…" Delete 'that'

Authors' response: Thank you for your input. We have made the change accordingly in the revised version. Please refer to line 64.

L93-95: The sentence refers to genome-wide association analyses but reference [13] deals with genomic prediction. It is not a GWAS study illustrating the value of the data to select donors with novel favorable alleles. Is it the most appropriate reference?

Authors' response: We apologize for the confusion caused. We have replaced the reference accordingly. Please refer to line 83.

L99-100: Check the following sentence, something is missing - "With the developing public access resources to enable next generations of scientists spend less time on generating and curing data, …"

Authors' response: Thank you for pointing this out. We have revised the sentence accordingly. Please refer to line 101-103 of the revised manuscript. The revised text reads as "With the development of publicly accessible resources, the next generations of scientists can focus more on research and innovation, reducing the burden of extensive phenotyping."

L141-142: What proportion of data were identified as outliers? Were they removed from the dataset or imputed again?

Authors' response: We appreciate your attention to the detail. We have included the information about the proportion of outliers into Supplementary table S2. All the outliers were removed from the dataset.

L206: 'environment accounts' or 'the environments account'

Authors' response: We apologize for the confusion caused. The revised text reads as "…environment (σ_e^2) accounts for the largest proportion of the total variation…" Please refer to line 230.

Click here to access/download
Supplementary Material
Supplementary Table-Yuan.et.al-20241203.xlsx
