# Supplementary material for: High-quality phenotypic and genotypic dataset of barley genebank core collection to unlock untapped genetic diversity
Source: Gigascience. 2025 Feb 11;14:giae121. doi: 10.1093/gigascience/giae121 (PMC11811526; doi:10.1093/gigascience/giae121)
Supplement: giae121_Response_to_Reviewer_Comments_Original_Submission [file giae121_response_to_reviewer_comments_original_submission.pdf]

Author's Response To Reviewer Comments

Manuscript GIGA-D-24-00417
Response to reviews

Dear Dr. Nogoy,
Thank you for considering our manuscript for publication in GigaScience. We appreciate the time and effort that you and the reviewers have taken to provide feedback on our manuscript, and we are grateful for the insightful comments and constructive suggestions. We have carefully addressed the points raised and incorporated them into the revised manuscript. The changes are highlighted in yellow in the revised version for easy reference. We have also made minor edits throughout the manuscript to improve readability and clarity. The adjustments do not alter the content or conclusions of the paper, but enhance the overall given information. We hope that these revisions substantially strengthen our manuscript and believe that it now meets the standards for publication in GigaScience.
Reviewer1 Comments to the Author:
This data note presents a comprehensive study on the integration of genebank resources with precision phenotyping and genotyping to enhance barley breeding. The authors have effectively highlighted the significance of genetic diversity in barley and the role of genebanks in supporting agricultural innovation. The topic is highly relevant to current challenges in agriculture, particularly in the context of food security and climate change. Although the study carries significant strengths making it qualified for publication in GigaScience but needs some reconsiderations to resolve some concerns issues before going to formal acceptance for publication and are given as under. The study has the potential to make a significant contribution to the field of barley breeding and genomics. With revisions addressing the identified areas for improvement, this note could be a strong candidate for publication.
Authors' response: Thank you for your thorough review and valuable feedback. We appreciate the time and effort you have invested in evaluating our work. We have addressed each of your comments and suggestions in detail below. Changes have been made and highlighted accordingly in the manuscript.
As an article of "Data Note", the title is overstated. The title should focus more on the data itself.
Authors' response: Thank you for your feedback. We appreciate your insight regarding the title of the manuscript. Based on the nature of a "Data Note" article, we have changed our title to "High-quality phenotypic and genotypic dataset of barley genebank core-collection to unlock untapped genetic diversity".
Summarize the main conclusions drawn from the results succinctly in the abstract.
Authors' response: We appreciate your suggestion. A brief conclusion has been included in the abstract. Please refer to line 41-43.
Try to add more background information on barley breeding and its challenges particularly emphasize the relevance of the research in the current agricultural landscape.
Authors' response: Thank you for your valuable feedback. We appreciate your suggestion to provide more background information on barley breeding and its challenges. We have expanded the introduction to include a short discussion of barley breeding, highlighting its significance in global importance. Specifically, we outlined the key challenges faced by breeders, such as adapting barley to changing climatic conditions, improving disease resistance, and enhancing yield stability (please refer to lines 53-55 and lines 57-60). Additionally, we emphasized the relevance of our research within the context of the current agricultural landscape, given the increasing demand for leveraging the genetic diversity harbored within plant genetic resources. We also discuss how advances in genomics and phenotyping, such as tools explored in our study, can contribute to addressing these challenges and improving the efficiency of barley breeding programs.
We believe these additions help contextualize our research and demonstrate its importance in advancing barley breeding to meet future agricultural demands. Thank you again for your insightful suggestion.
State the specific objectives of the study more explicitly and in a separate paragraph and briefly outline the structure of the manuscript at the end of this section.
Authors' response: Thank you for your constructive suggestion. We have revised the context to include a distinct part that explicitly states the specific objectives of the study. This paragraph now clearly outlines our aims. Please refer to lines 91-96.
It is suggested to summarize/describe the environmental conditions under which the trials were conducted.
Authors' response: Thank you for your valuable feedback. We have added a detailed summary of the

environmental conditions under which the trials were conducted. This section now describes the average temperature across three years for each location. Please refer to lines 123-136.
Can you please provide justification for the choice of traits and methods used in the study and elaborate on the phenotyping methods, including any specific protocols followed.
Authors' response: Thank you for your insightful comments. We appreciate the opportunity to provide further justification for the choice of traits and methods used in this study.
The agronomic traits (heading date, plant height, and lodging) were selected for their importance in barley adaptability, yield potential, and harvestability, while disease traits address key breeding challenges for durable resistance. Phenotyping followed standardized protocols: heading date as days to 50% heading from January 1st for winter type and from the sowing date for the spring type, plant height measured at maturity, and lodging scored visually. The four disease traits [Puccinia hordei (PUC), Blumeria graminis hordei (BLU), Ramularia collo-cygni (RAM), and Rhychnosporium commune (RHY)] were included because these diseases are prevalent in the study's geographic region and are known to significantly affect crop health and yield. Disease resistance was assessed under field conditions to ensure robust and reproducible data, directly linking genebank materials to breeding applications.
We have integrated your comment in the revised manuscript to enhance transparency and to ensure that the methods used are well-justified. Please refer to line 140-148.
Provide some details on the alpha lattice design used in the trials and try to add a description of the statistical methods used for data analysis along with significance level of results obtained.
Authors' response: Thank you for your valuable feedback. We appreciate your suggestion to provide more details on the alpha lattice design and statistical methods used in the trials. A detailed description of the alpha lattice design employed in our trials is available in the reference [40]. For clarity, we have included a brief summary of the design in the revised manuscript, ensuring that key elements (please refer to lines 133-137). Regarding the statistical methods, we have included a detailed description of the analyses performed in the "Phenotypic data analyses" section, please refer to lines 149-168.
Clarify the criteria used for selecting SNPs for analysis besides providing more details on the imputation methods used for missing data.
Authors' response: We appreciate your attention to detail. To address this, we have added a sentence in the manuscript specifying the criteria for SNP selection. These criteria include minor allele frequency > 0.05 and missing rate < 0.1, and an r2 cutoff of 0.2 was set to prune markers.
Regarding the imputation methods, Beagle was chosen due to its reliability in phasing and imputing genotypes, leveraging linkage disequilibrium to infer missing data accurately. The process involved phasing the genotypes and imputing missing data. Please refer to lines 186-188.
Given the limitations of Principal Coordinates Analysis (PCoA) in addressing population structure and ancestry issues, could you clarify the rationale behind choosing PCoA instead of utilizing methods like Admixture or STRUCTURE, which are specifically designed to provide insights into these aspects?
Authors' response: Thank you for your insightful question regarding the choice of Principal Coordinate Analysis (PCoA) over methods like Admixture or STRUCTURE for this study. To complement our analyses, we have add the admixture results into the manuscript. Please refer to lines 276-284.
PCoA reduces a multi-dimensional dataset to a much smaller number of dimensions that allows for visual exploration and compact quantitative summaries. With admixture or STRUCTURE proportion inference, individuals in a sample are modeled as having a proportion of their genome derived from each of several source populations. The goal is to infer the proportions of ancestry from each source population, and these proportions can be used to produce compact visual summaries that reveal the existence of population structure in a sample. Another qualitative difference is that PCoA produces consistent results as more dimensions are added, whereas admixture-based methods produce qualitatively different results with different numbers of genetic components (K). Although consistency may seem a desirable property, there can be benefits to the different perspectives obtained by using different numbers of factors. In our study, the optimal number of genetic components (K) was tested using admixture based on cross-validation function (--cv), please refer to lines 198-200.
This note mentions the integration of genotypic and phenotypic data but does not provide a clear framework or methodology for how this integration was/would be achieved or its significance. Include a section that explicitly describes the integration process of genotypic and phenotypic data. Discuss the challenges faced/would be faced during integration and how they could be addressed. Highlight the importance of this integration for future breeding programs.
Authors' response: Thank you for your insightful feedback. The quality check of the integration of genotypic and phenotypic data in our study was achieved by genome-wide prediction coupled with 100 times five-fold

cross-validation. Overall, the genomic-phenotypic data interoperability was in general high (please refer to lines 288-307). Additionally, genome-wide association studies were used to identify genetic markers associated with the trait, providing actionable insights for breeding decisions (please refer to reference [14]).

A key challenge in this integration process was handling large-scale datasets with missing genotypic values. To address this, we employed phasing and imputation with Beagle to ensure high-quality genotype data, followed by filtering for minor allele frequency and missing rates to enhance reliability. As for the phenotypic data, standardized field trials and precise trait phenotyping minimized environmental noise.

The integration of genotypic and phenotypic data is critical for future breeding programs, offering a framework to validate markers identified through GWAS and use them in marker-assisted selection to identify potential donors for traits of interest. Moreover, by linking genetic diversity with key agronomic traits, breeders can make more informed decisions by generate more accurate predictions about trait performance, improving the efficiency of variety development and accelerating breeding cycles. Please refer to lines 79-83.

Improve the narrative flow by logically grouping related findings together.

Authors’ response: Thank you for your valuable feedback. We have carefully reviewed the structure of the manuscript and have made revisions to improve the narrative flow. Specifically, we’ve provided clearer transitions between sections to help the reader follow the flow of the most critical findings. We hope these changes enhance the clarity and readability of the manuscript.

Emphasize the most important findings in the text to guide the reader.

Authors’ response: Thank you for your valuable feedback. Key results are now highlighted in the sub-title to guide the reader. We’ve ensured that the central conclusions are presented in a way that underscores their significance in the context of the study’s objectives.

Briefly mention any limitations observed in the results. Acknowledge any limitations of the study and their potential impact on the findings.

Authors’ response: Thank you for your insightful feedback. We have acknowledged the limitations of our study in the revised manuscript. One key limitation is the relatively low heritability observed for RHY in spring population and RAM in the winter population. The low disease pressure resulted in limited phenotypic variation and reduced heritability, as the environmental conditions did not sufficiently differentiate the responses of the genotypes. This diminished the ability to detect genetic contributions to the two traits.

I think authors should consider to clearly articulate the practical implications of the findings for barley breeding or agricultural practices. If their findings fail to connect to real-world applications, the relevance of the study may be questioned.

Authors’ response: Thank you for bringing this to our attention. This integrated approach also enables breeders to identify genetic markers associated with desirable traits, accelerating the development of crops with improved yield, disease resistance, or other key attributes. By combining genotypic and phenotypic data, breeders can also make more accurate predictions about trait performance, ultimately improving the efficiency of selection processes. We believe our dataset will underscore the importance of this approach in advancing breeding programs. Please refer to line 97-103.

Briefly add how environmental conditions may have influenced phenotyping results.

Authors’ response: Thank you for this valuable suggestion. We have updated the section to include a brief explanation of how non-conductive environmental conditions may have influenced the phenotyping results. Specifically, we acknowledge that variations in temperature, humidity, and precipitation across the different climate zones could impact the expression of the trait under study. For example, stress from extreme temperatures or inconsistent rainfall may alter plant growth or other phenotypic traits, potentially affecting data consistency and interpretation. Please refer to line 233-237.

Briefly add potential biological mechanisms underlying the observed traits.

Authors’ response: Thank you for your feedback and for bringing this to our attention. We appreciate the importance of discussing potential biological mechanisms for additional context. However, the primary scope of this paper is centered on the description of the dataset. A detailed examination of biological mechanisms would extend beyond the intended focus and depth of our work. Keeping the manuscript concise and aligned with our original objectives is essential to its clarity.

Integrate key points from the findings to reinforce the conclusions drawn.

Authors’ response: Thank you for your valuable suggestion. We have revised the conclusion section to better integrate key points from the findings and reinforce the conclusions drawn from our study. Please refer to line 41-43.

Reviewer2 Comments to the Author:
The manuscript by Yuan et al describes the public release of phenotypic and genotypic data of a large set of barley landraces, genebank accessions, and elite materials. The authors provide a comprehensive dataset, whole genome sequencing data together with phenotypic data for disease and agronomic traits, that will be of interest for future genetic research in barley. The document is well written, highlighting the value of the data provided. I have some comments or suggestions to further improve the document, as follows:
Authors' response: We sincerely thank you for your thoughtful review and valuable feedback on our manuscript. We appreciate your recognition of the importance of the issues addressed in our study and your acknowledgment of the manuscript's quality.
Line 64: "enormous efforts that have been made…" Delete 'that'
Authors' response: Thank you for your input. We have made the change accordingly in the revised version. Please refer to line 64.
L93-95: The sentence refers to genome-wide association analyses but reference [13] deals with genomic prediction. It is not a GWAS study illustrating the value of the data to select donors with novel favorable alleles. Is it the most appropriate reference?
Authors' response: We apologize for the confusion caused. We have replaced the reference accordingly. Please refer to line 83.
L99-100: Check the following sentence, something is missing - "With the developing public access resources to enable next generations of scientists spend less time on generating and curing data, …"
Authors' response: Thank you for pointing this out. We have revised the sentence accordingly. Please refer to line 101-103 of the revised manuscript. The revised text reads as "With the development of publicly accessible resources, the next generations of scientists can focus more on research and innovation, reducing the burden of extensive phenotyping."
L141-142: What proportion of data were identified as outliers? Were they removed from the dataset or imputed again?
Authors' response: We appreciate your attention to the detail. We have included the information about the proportion of outliers into Supplementary table S2. All the outliers were removed from the dataset.
L206: 'environment accounts' or 'the environments account'
Authors' response: We apologize for the confusion caused. The revised text reads as "…environment ($\sigma\_e\^2$) accounts for the largest proportion of the total variation…" Please refer to line 230.
