# Supplementary material for: High-quality phenotypic and genotypic dataset of barley genebank core collection to unlock untapped genetic diversity
Source: Gigascience. 2025 Feb 11;14:giae121. doi: 10.1093/gigascience/giae121 (PMC11811526; doi:10.1093/gigascience/giae121)
Supplement: giae121_Reviewer_1_Report_Original_Submission — Bo Song -- 11/3/2024 [file giae121_reviewer_1_report_original_submission.pdf]

Reviewer Report

Title: High-quality phenotypic and genotypic dataset of barley genebank core-collection to unlock untapped genetic diversity

Version: Original Submission **Date:** 11/3/2024

Reviewer name: Bo Song

Reviewer Comments to Author:

This data note presents a comprehensive study on the integration of genebank resources with precision phenotyping and genotyping to enhance barley breeding. The authors have effectively highlighted the significance of genetic diversity in barley and the role of genebanks in supporting agricultural innovation. The topic is highly relevant to current challenges in agriculture, particularly in the context of food security and climate change. Although the study carries significant strengths making it qualified for publication in GigaScience but needs some reconsiderations to resolve some concerns issues before going to formal acceptance for publication and are given as under:* As an article of "Data Note", the title is overstated. The title should focus more on the data itself.* Summarize the main conclusions drawn from the results succinctly in the abstract.* Try to add more background information on barley breeding and its challenges particularly emphasize the relevance of the research in the current agricultural landscape.* State the specific objectives of the study more explicitly and in a separate paragraph and briefly outline the structure of the manuscript at the end of this section.* It is suggested to summarize/describe the environmental conditions under which the trials were conducted.* Can you please provide justification for the choice of traits and methods used in the study and elaborate on the phenotyping methods, including any specific protocols followed.* Provide some details on the alpha lattice design used in the trials and try to add a description of the statistical methods used for data analysis along with significance level of results obtained.* Clarify the criteria used for selecting SNPs for analysis besides providing more details on the imputation methods used for missing data.* Given the limitations of Principal Coordinates Analysis (PCoA) in addressing population structure and ancestry issues, could you clarify the rationale behind choosing PCoA instead of utilizing methods like Admixture or STRUCTURE, which are specifically designed to provide insights into these aspects?* This note mentions the integration of genotypic and phenotypic data but does not provide a clear framework or methodology for how this integration was/would be achieved or its significance. Include a section that explicitly describes the integration process of genotypic and phenotypic data. Discuss the challenges faced/would be faced during integration and how they could be addressed. Highlight the importance of this integration for future breeding programs.* Improve the narrative flow by logically grouping related findings together.* Emphasize the most important findings in the text to guide the reader.* Briefly mention any limitations observed in the results.* Acknowledge any limitations of the study and their potential impact on the findings.* I think authors should consider to clearly articulate the practical implications of the findings for barley breeding or agricultural practices. If their findings fail to connect to real-world applications, the relevance of the study may be questioned.* Briefly add how environmental conditions may have influenced phenotyping results.* Briefly add potential biological

mechanisms underlying the observed traits.* Integrate key points from the findings to reinforce the conclusions drawn.The study has the potential to make a significant contribution to the field of barley breeding and genomics. With revisions addressing the identified areas for improvement, this note could be a strong candidate for publication.

Level of Interest

Please indicate how interesting you found the manuscript: Choose an item.

Quality of Written English

Please indicate the quality of language in the manuscript: Choose an item.

Declaration of Competing Interests

Please complete a declaration of competing interests, considering the following questions:

- Have you in the past five years received reimbursements, fees, funding, or salary from an organisation that may in any way gain or lose financially from the publication of this manuscript, either now or in the future?
- Do you hold any stocks or shares in an organisation that may in any way gain or lose financially from the publication of this manuscript, either now or in the future?
- Do you hold or are you currently applying for any patents relating to the content of the manuscript?
- Have you received reimbursements, fees, funding, or salary from an organization that holds or has applied for patents relating to the content of the manuscript?
- Do you have any other financial competing interests?
- Do you have any non-financial competing interests in relation to this paper?

If you can answer no to all of the above, write 'I declare that I have no competing interests' below. If your reply is yes to any, please give details below.

I declare that I have no competing interests.

I agree to the open peer review policy of the journal. I understand that my name will be included on my report to the authors and, if the manuscript is accepted for publication, my named report including any attachments I upload will be posted on the website along with the authors' responses. I agree for my report to be made available under an Open Access Creative Commons CC-BY license (http://creativecommons.org/licenses/by/4.0/). I understand that any comments which I do not wish to be included in my named report can be included as confidential comments to the editors, which will not be published.

Choose an item.

To further support our reviewers, we have joined with Publons, where you can gain additional credit to further highlight your hard work (see: https://publons.com/journal/530/gigascience). On publication of this paper, your review will be automatically added to Publons, you can then choose whether or not to claim your Publons credit. I understand this statement.

Yes Choose an item.
