# Supplementary material for: High-quality phenotypic and genotypic dataset of barley genebank core collection to unlock untapped genetic diversity
Source: Gigascience. 2025 Feb 11;14:giae121. doi: 10.1093/gigascience/giae121 (PMC11811526; doi:10.1093/gigascience/giae121)
Supplement: giae121_Reviewer_2_Report_Original_Submission — Ana Casas -- 11/12/2024 [file giae121_reviewer_2_report_original_submission.pdf]

Reviewer Report

Title: High-quality phenotypic and genotypic dataset of barley genebank core-collection to unlock untapped genetic diversity

Version: Original Submission **Date:** 11/12/2024

Reviewer name: Ana Casas

Reviewer Comments to Author:

The manuscript by Yuan et al describes the public release of phenotypic and genotypic data of a large set of barley landraces, genebank accessions, and elite materials. The authors provide a comprehensive dataset, whole genome sequencing data together with phenotypic data for disease and agronomic traits, that will be of interest for future genetic research in barley. The document is well written, highlighting the value of the data provided.I have some comments or suggestions to further improve the document, as follows:Line 64: "enormous efforts that have been made…" Delete 'that'L93-95: The sentence refers to genome-wide association analyses but reference [13] deals with genomic prediction. It is not a GWAS study illustrating the value of the data to select donors with novel favorable alleles. Is it the most appropriate reference?L99-100: Check the following sentence, something is missing - "With the developing public access resources to enable next generations of scientists spend less time on generating and curing data, …"L141-142: What proportion of data were identified as outliers? Were they removed from the dataset or imputed again?L206: 'environment accounts' or 'the environments account'

Level of Interest

Please indicate how interesting you found the manuscript: Choose an item.

Quality of Written English

Please indicate the quality of language in the manuscript: Choose an item.

Declaration of Competing Interests

Please complete a declaration of competing interests, considering the following questions:

- Have you in the past five years received reimbursements, fees, funding, or salary from an organisation that may in any way gain or lose financially from the publication of this manuscript, either now or in the future?
- Do you hold any stocks or shares in an organisation that may in any way gain or lose financially from the publication of this manuscript, either now or in the future?
- Do you hold or are you currently applying for any patents relating to the content of the manuscript?

- Have you received reimbursements, fees, funding, or salary from an organization that holds or has applied for patents relating to the content of the manuscript?
- Do you have any other financial competing interests?
- Do you have any non-financial competing interests in relation to this paper?

If you can answer no to all of the above, write 'I declare that I have no competing interests' below. If your reply is yes to any, please give details below.

I declare that I have no competing interests

I agree to the open peer review policy of the journal. I understand that my name will be included on my report to the authors and, if the manuscript is accepted for publication, my named report including any attachments I upload will be posted on the website along with the authors' responses. I agree for my report to be made available under an Open Access Creative Commons CC-BY license (http://creativecommons.org/licenses/by/4.0/). I understand that any comments which I do not wish to be included in my named report can be included as confidential comments to the editors, which will not be published.

Choose an item.

To further support our reviewers, we have joined with Publons, where you can gain additional credit to further highlight your hard work (see: https://publons.com/journal/530/gigascience). On publication of this paper, your review will be automatically added to Publons, you can then choose whether or not to claim your Publons credit. I understand this statement.

Yes Choose an item.
